# Boosting Water Oxidation through In Situ Electroconversion of Manganese Gallide: An Intermetallic Precursor Approach

**DOI:** 10.1002/anie.201909904

**Published:** 2019-10-15

**Authors:** Prashanth W. Menezes, Carsten Walter, Jan Niklas Hausmann, Rodrigo Beltrán‐Suito, Christopher Schlesiger, Sebastian Praetz, Valeriy Yu. Verchenko, Andrei V. Shevelkov, Matthias Driess

**Affiliations:** ^1^ Department of Chemistry: Metalorganics and Inorganic Materials Technische Universität Berlin Straße des 17 Juni 135, Sekr. C2 10623 Berlin Germany; ^2^ Institute of Optics and Atomic Physics Technische Universität Berlin Hardenbergstraße 36 10623 Berlin Germany; ^3^ Department of Chemistry Lomonosov Moscow State University 119991 Moscow Russia

**Keywords:** electrocorrosion, MnO_*x*_ minerals, oxygen evolution reaction, renewable energy, water-splitting electrocatalysts

## Abstract

For the first time, the manganese gallide (MnGa_4_) served as an intermetallic precursor, which upon in situ electroconversion in alkaline media produced high‐performance and long‐term‐stable MnO_x_‐based electrocatalysts for water oxidation. Unexpectedly, its electrocorrosion (with the concomitant loss of Ga) leads simultaneously to three crystalline types of MnO_*x*_ minerals with distinct structures and induced defects: birnessite δ‐MnO_2_, feitknechtite β‐MnOOH, and hausmannite α‐Mn_3_O_4_. The abundance and intrinsic stabilization of Mn^III^/Mn^IV^ active sites in the three MnO_*x*_ phases explains the superior efficiency and durability of the system for electrocatalytic water oxidation. After electrophoretic deposition of the MnGa_4_ precursor on conductive nickel foam (NF), a low overpotential of 291 mV, comparable to that of precious‐metal‐based catalysts, could be achieved at a current density of 10 mA cm^−2^ with a durability of more than five days.

## Graphical abstract

**Partnership in action**: Electrocorrosion and concomitant leaching of gallium from the structurally compelling intermetallic manganese gallide MnGa_4_ lead to a highly active electrocatalyst system for water oxidation in alkaline media. Under the electrochemical reaction conditions, three distinct crystalline and highly active MnO_x_ phases result simultaneously with considerably low overpotentials and durability of over five days.

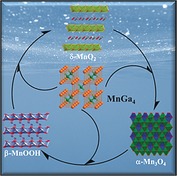

Electrochemical water splitting through the hydrogen evolution reaction (HER) and oxygen evolution reaction (OER) has been regarded as a promising technology that is renewable, sustainable, and eco‐friendly.^1^ Although highly efficient HER electrodes exist, the OER is the bottleneck in water splitting.^2^ The complex OER process involves multiple proton‐coupled electron‐transfer steps with high‐energy intermediates and is both thermodynamically and kinetically demanding.^3^ Currently, ruthenium‐ and iridium‐based catalysts have shown the best activity for OER; however, the high cost, low natural abundance, and limited long‐term stability restrict their practical application on a large scale.^4^ Therefore tremendous effort has been devoted to develop alternative highly efficient and durable electrocatalysts based on low‐cost earth‐abundant elements.^5^

In nature, photosynthetic water oxidation is mediated by a flexible Mn_4_CaO_5_ cubane‐like cluster in the photosystem II.^6^ Scientist have been inspired by this process in nature and have investigated artificial Mn‐based catalysts^7^ owing to the low cost, high natural abundance, low toxicity, and rich redox chemistry of manganese. Over the years, numerous crystalline and amorphous manganese oxides have been probed for acidic, neutral, or alkaline water oxidation.^8^ Most recently, many experimental and theoretical investigations have been dedicated to unraveling the active sites of manganese oxides.7b, 9 It has been shown that the presence of Mn^III^ is the decisive factor for the promotion of OER, where Mn^III^ in the t_2g_^3^ e_g_^1^ high‐spin configuration leads to Jahn–Teller distortion, providing longer Mn−O bonds with the necessary flexibility to facilitate O−O bond formation.^10^ Importantly, it was concluded that at pH<9, rapid consumption of Mn^III^ occurs through the disproportionation reaction (2 Mn^III^→Mn^II^+Mn^IV^) that results in large overpotentials. However, the comproportionation of Mn^II^ and Mn^IV^ to form Mn^III^ transpires at pH≥9, thus lowering overpotentials and enhancing OER.7a, 10b Consequently, notable efforts have also been undertaken to stabilize and facilitate the generation of Mn^III^ at the surface of catalysts and to understand the catalytic activity of different Mn‐based species in light of their crystal phases, chemical composition, polymorphism, morphology, and microscopic structures (defects).^11^ Most of the manganese oxide based (MnO_*x*_) water oxidation catalysts are usually prepared by precipitation, hydrothermal synthesis, solid‐state reactions, and electrodeposition; the resulting materials have either low catalytic activity (overpotentials >400 mV) or limited stability (< few hours). Thus, it is very challenging and attractive to gain synthetic access to reliably active MnO_*x*_ materials other than by starting from common manganese oxides; these new materials could display promising catalytic activities and provide profound insights on the required MnO_*x*_ structures for OER.^12^ We have discovered that intermetallic manganese phases could serve as a new class of precursor materials for the production of MnO_*x*_ catalysts with superior performance and durability in electrocatalytic OER.

Intermetallic compounds possess unique chemical, physical, and electronic properties as well as distinct atomic structures.^13^ The low resistivity and higher adsorption properties of intermetallics compared to oxides, predestinates them as suitable electrocatalysis, in particularly for HER, where the extent of atomic ordering and the relative concentration of both metals drastically influence the overall efficiency;^13^, ^14^ however, their activity for OER is rather unexplored. Here we report that manganese gallide (MnGa_4_) is a new precursor material for electrocatalytic OER; MnGa_4_ is a d–sp bonded Hume–Rothery intermetallic compound with strong directional (covalent) bonds and has attracted great interest in physics due to its metallic behavior and antiferromagnetic ordering.^15^ It turned out that MnGa_4_ undergoes in situ electroconversion in alkaline media to form different MnO_*x*_ mineral types with distinct structures and induced defects which boost OER.

Silvery gray crystalline samples of intermetallic MnGa_4_ are accessible by a high‐temperature solid‐state technique (see the Supporting Information). The phase purity of MnGa_4_ was confirmed by powder X‐ray diffraction (PXRD) analysis, which displayed sharp reflections corresponding to the theoretical pattern of the single crystal (Figure S1 in the Supporting Information).^15^ The overall structure of MnGa_4_ can be viewed as a defect CsCl structure where three‐fourths of the Cs atoms are eliminated to form corner‐linked cubes (MnGa_8/2_) as shown in Figure 1 a. The morphology of MnGa_4_ was evaluated by scanning electron microscopy (SEM) which exhibited irregularly shaped particles with varying sizes (Figures S2 and S3). To determine the element distribution in MnGa_4_ particles, energy‐dispersive X‐ray (EDX) mapping analysis was conducted using SEM which confirmed the homogenous distribution of Mn and Ga elements with an atomic ratio close to 1:4 (Figure 1 c–e; Figures S4–S8; Table S1). The selected‐area electron diffraction pattern (SAED) combined with transmission electron microscopy (TEM) images disclosed highly crystalline features of MnGa_4_. The lattice spacing of the particles was resolved by high‐resolution (HR) TEM to be around 0.39 and 0.2 nm corresponding to the (011) and (022) crystallographic planes of MnGa_4_ (Figure 1 b). The SAED pattern displayed intense diffractions spots that could be assigned to the crystallographic planes (112), (022), and (222) at 0.23, 0.20, and 0.16 nm, and are consistent with the PXRD pattern (inset in Figure 1 b; Figures S9 and S10). The presence of Mn and Ga was confirmed by EDX spectrum, while the composition of the material was confirmed by inductively coupled plasma atomic emission spectroscopy (ICP‐AES; Figure S11; Table S2). The Fourier transform infrared spectra (FTIR) exhibited vibrations for Mn‐Ga (Figure S12). The electronic structure of the MnGa_4_ material was further probed by X‐ray absorption spectroscopy (XAS). The Mn and Ga *K*‐edge X‐ray absorption near‐edge structure (XANES) serves as a qualitative spectroscopic fingerprint for the 1s‐to‐4p transitions to assist in the identification of Mn and Ga species present in MnGa_4_ (Figure S13). The shape of the Mn *K*‐edge XANES, measured with respect to various manganese references, overlaps strongly with that of metallic Mn, suggesting that most of the Mn possesses metallic character.^16^ Similarly, the Ga *K*‐edge XANES spectrum closely resembled that of Ga metal as reported previously.^17^

**Figure 1:**
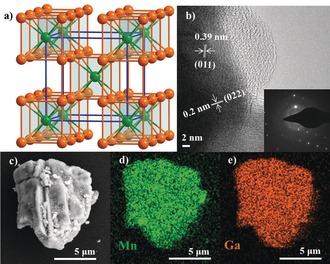
a) Crystal structure (Mn: green; Ga: orange), b) HR‐TEM image displaying well‐resolved lattice fringes with a spacing of 0.39 and 0.20 nm indicative of (011) and (022) planes with SAED pattern in the inset, and c–e) EDX elemental mapping of intermetallic MnGa_4_.

The surface chemical composition, bonding, and oxidation states of MnGa_4_ were unveiled by X‐ray photoelectron spectroscopy (XPS; Figures S14 and S15). The Mn 2p_3/2_ and Mn 2p_1/2_ spectra exhibited sharp peaks at the binding energies of 642.3 eV and 654.2 eV, which are close to the oxidation state of Mn^IV^ (MnO_2_).^18^ The oxidation state of Mn in MnGa_4_ was further deduced from the Mn 3p spectra, which typically measure ≈47.5 eV for Mn^II^, ≈48.5 eV for Mn^III,^ and ≈50 eV for Mn^IV^. The resulting binding energy value of 50.2 eV corresponds to Mn^IV^.^19^ Interestingly, the higher oxidation states of Mn in comparison to XANES suggest the unavoidable air oxidation at the surface (XPS is surface sensitive) of the particles, which is typical for intermetallics.^14^ In Ga 2p, the binding energy of 1116.4 eV attained for Ga 2p_3/2_ is very similar to that of elemental Ga (1116.4 eV) and the second peak observed at the binding energy of 1118.2 eV could be corroborated with Ga bonded to an oxo species due to surface passivation.^20^ As intermetallic MnGa_4_ maintains metallic character, Mn metal (cubic, *I*‐43*m*, No. 217) was directly chosen as a reference to deduce a clear advantage of as‐synthesized phase and characterized thoroughly (Figures S16–S22).

The electrocatalytic activity of the MnGa_4_ precatalyst towards the OER was investigated in 1 m aqueous KOH using cyclic voltammetry (CV). MnGa_4_ was first deposited on high‐surface‐area, conductive, and open‐pore 3D nickel foam (NF) by electrophoretic deposition and the resulting coated NF was used directly as the working electrode. Representative CV curves in Figure 2 a show the geometric current density plotted against applied potential (vs. reversible hydrogen electrode (RHE)) for intermetallic MnGa_4_ relative to metallic Mn. Moreover, the electrocatalytic activity of the bare NF (and with EPD protocol) was also incorporated as a blank control (Figure S23). Surprisingly, MnGa_4_/NF displayed excellent catalytic OER activity, reaching a current density of 10 mA cm^−2^ and 100 mA cm^−2^ at overpotentials of 291 and 402 mV, whereas the metallic Mn was inferior, with overpotentials 425 and 560 mV, respectively, at the same current densities (see Figure S24 for mass‐normalized activity). The NF has poor OER activity. When cycled between 1.15 and 1.45 V (vs. RHE), a reversible redox couple was obtained for both MnGa_4_/NF and Mn/NF corresponding to the oxidation of low‐valent manganese species to their higher valences (Figure S25).12a Tafel plots evaluated the OER catalytic kinetics, and a Tafel slope of 98 mV dec^−1^ was recorded for MnGa_4_/NF, which is smaller than that of Mn/NF (109 mV dec^−1^), suggesting a more favorable OER rate at the MnGa_4_/NF electrode (Figure S26). The electrochemically active surface areas (ECSAs) were estimated from the electrochemical double‐layer capacitance (*C*_dl_), and the obtained *C*_dl_ values for MnGa_4_/NF and Mn/NF were 4.58 and 2.63 mF cm^−2^, respectively (Figure S27).^21^ From the *C*_dl_ values and the specific capacitance of the material (*C*_s_) per unit area, the ECSA was calculated to bes 2.7 cm^2^ for MnGa_4_/NF and 1.54 cm^2^ for Mn/NF, demonstrating the accessibility to a higher density of active sites in MnGa_4_ favoring efficient adsorption and transfer of reactants to improve the electrochemical reaction.^21^ Furthermore, to evaluate the electrode kinetics under OER, which provides detailed information on the interfacial reactions and behavior of the catalysts, electrochemical impedance spectroscopy (EIS) was performed.^21^ (Figure 2 b; Figure S28). The substantially reduced charge transfer resistance achieved for MnGa_4_/NF in comparison to Mn/NF suggests rapid charge‐transfer kinetics between catalyst and electrolyte during the OER process.

**Figure 2:**
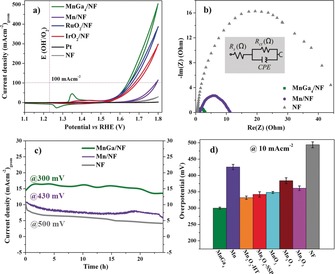
a) OER catalytic performances, b) EIS responses, c) long‐term stability curves of intermetallic MnGa_4_, metallic Mn, and bare NF. The activity comparison of MnGa_4_ with various synthetic manganese oxides (mass loading of 2 mg cm^−2^) is presented in (d).

One of the drawbacks of Mn‐based catalysts is their decrease in activity within a few hours of operation. To address this, the chronoamperometric stability (CA) of MnGa_4_/NF and Mn/NF was investigated. In Figure 2 c shows a more stable curve for MnGa_4_/NF, whereas a decrease in current density was observed for Mn/NF when measured up to 24 h. In fact, the ECSA of MnGa_4_/NF and Mn/NF calculated after CA was three times higher than that of the as‐prepared materials which were also normalized showing a better intrinsic activity for MnGa_4_/NF (Figures S29 and S30). Motivated by this, we prolonged the CA measurements of MnGa_4_/NF for over 5 days and demonstrated long‐term durability at relatively low overpotentials (Figure S31). Besides, the amount of experimentally measured O_2_ matched very well with theoretically calculated values, indicating a Faradaic efficiency of nearly 100 % (Supporting Information). Additionally, the bare NF subjected to CA stability tests produced only low activity, which also rules out the possible influence of NF in OER.

The OER activities of MnGa_4_/NF and Mn/NF were further compared to benchmark noble‐metal‐based catalysts (RuO_2_, IrO_2_, and Pt) (Figure 2 a). Interestingly, the attained OER overpotentials of MnGa_4_/NF (293 mV and 420 mV for 10 mA cm^−2^ and 100 mA cm^−2^, respectively) were also slightly superior to benchmark RuO_2_/NF (310 mV for 10 mA cm^−2^) and clearly better than IrO_2_/NF (445 mV for 10 mA cm^−2^). Moreover, known highly active manganese oxides were prepared and deposited on NF (Figure S32). Remarkably, the MnGa_4_ precatalyst on NF outperformed other MnO_*x*_ materials underlining an advantage of the intermetallic structure. Also, the electrocatalytic OER performance of MnGa_4_/NF was directly compared to that of other literature‐known promising Mn‐based materials and transition‐metal‐based catalysts on NF, and MnGa_4_/NF stands out (Tables S6 and S7).^22^ Furthermore, the films deposited on FTO and carbon cloth displayed a very similar trend to those on NF (Figures S33 and S34).

To gain in‐depth insight into the origin of the excellent electrocatalytic activity, we characterized intermetallic MnGa_4_ after OER stability tests (OER CA) together with elemental Mn. Interestingly, the PXRD pattern of MnGa_4_ after OER CA displayed the presence of three distinct crystalline MnO_*x*_ phases: birnessite δ‐MnO_2_, feitknechtite β‐MnOOH, and hausmannite α‐Mn_3_O_4_ along with the MnGa_4_ (Figure S35). The SEM images of MnGa_4_ displayed severe morphological changes. The very porous nature of the particles hints at an in situ electroconversion of the as‐synthesized phase under alkaline OER conditions (Figure S36). This was further substantiated by the EDX mapping, where Mn and O were homogeneously distributed within the particles, and Ga atoms mostly disappeared from the structure (Figure S37; Table S2). The distribution of the elements obtained by EDX mapping showed more than 90 % loss of Ga under OER within 24 h to form the crystalline MnO_*x*_ phases, which is consistent with the result deduced from the ICP‐AES analysis. This implies that the electrocorrosion process probably starts at the surface of the particle and penetrates deep inside forming disordered and defect‐rich MnO_*x*_ during prolonged electrolysis. Like SEM, the TEM images also confirmed a severe loss of Ga from the particles transforming MnGa_4_ completely into a hollow porous structure (Figure 3 a, Figures S38 and S39). A closer look at the edge of the nanostructure in HRTEM suggested a lattice spacing of 0.7 nm, which can be assigned to the (001) plane of birnessite δ‐MnO_2_. Furthermore, the distance of 0.26 nm could be ascribed to (301) planes of feitknechtite β‐MnOOH or (311) planes of hausmannite α‐Mn_3_O_4_ structures, which is in accordance with the PXRD. This observation is quite different from other Mn‐based materials where an amorphous shell is usually formed on a crystalline core.^12^ The FTIR spectrum after OER exhibited bands corresponding to surface hydroxylation, Mn−OH as well as Mn−O, further confirming the derived conclusions (Figure S40). The Mn *K*‐edge XANES spectrum of MnGa_4_ after OER was measured with several manganese standards and used as a basis for comparison (Figure 3 c; Figure S41). Edge positions and the shape of the spectrum near the edge suggested the Mn has an oxidation state intermediate between Mn^III^ and Mn^IV^.^16^ The Ga *K*‐edge XANES spectra (Figure 3 d) indicated the oxidation of metallic Ga to Ga_2_O_3_.^17^ The Mn 2p and Mn 3p XPS spectrum did not deviate much after OER compared to the as‐prepared MnGa_4_, indicating the oxidation state of Mn at the surface was close to Mn^IV^ (Figure 3 b; Figures S42 and S43).^18^, ^19^ In the case of Ga 2p, the peaks corresponding to Ga were absent, confirming the massive loss of Ga from the surface of MnGa_4_ under in situ electrocatalytic OER. The O 1s spectrum was deconvoluted into three peaks corresponding to the formation of Mn oxide, hydroxylated MnOH/‐OOH sites, and adsorbed water onto the surface.11b, 23 The transformation of MnGa_4_ during OER was also investigated at various potentials and after CV cycling (Figures S44, S45–S47, Table S2). Similarly, the presence of Ni incorporated from NF into the active MnO_x_ structure was excluded, although a minimal influence of Ni in OER activity cannot be ruled out completely (Figures S48–S54). Besides, the suspension of MnGa_4_ in 1 m KOH for 24 h confirmed that the precatalysts were transformed by electroconversion and not by chemical etching (Figure S55; Table S5). A similar transformation also resulted in elemental Mn under OER conditions, and the detailed characterizations and results have been described in Figures S56–S63.

**Figure 3:**
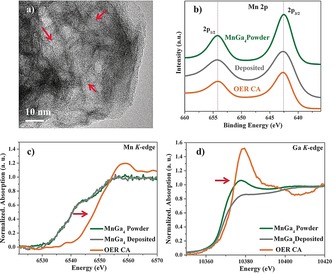
a) HR‐TEM image of MnGa_4_ after OER showing porous structure. The Mn 2p XPS spectra (b) and Mn/Ga *K*‐edge XANES (c–d) spectra of as‐synthesized, as‐deposited, and post OER films of MnGa_4_ (red arrow indicates oxidation).

Lately, several Mn oxides such as crystalline MnO, MnO_2_ (α, β, ν, δ), Mn_3_O_4_, MnOOH (ν), Mn_2_O_3_, Mn_5_O_8_, and amorphous MnO_*x*_ phases have been synthesized and illustrated as active structures for OER.6a, 7a, 7e Further, the presence of higher amounts of di‐μ‐oxo bridges within the Mn oxide, the simultaneous presence of Mn^III^ and Mn^IV^, and the stabilization of Mn^III^ are regarded the crucial factors for the evolution of active Mn‐oxide based catalysts.7d, 8c, 8d, 10b, 24 A significant effort has been expended to understand the active sites of the birnessite δ‐MnO_2_ structure where Mn^III^ within the Mn^IV^O_2_ layers has been considered as vital for water oxidation.9b, 25 They form a defective structure and enhance the adsorption of the OH intermediate in the OER.^25^ Alternatively, ν‐Mn^III^OOH has gained a lot of interest as it exhibits better performance than other MnO_*x*_ materials; however, layered β‐Mn^III^OOH has never been interpreted as the active structure for the water oxidation.24b Besides, the presence of a hausmannite‐like intermediate (Mn_3_O_4_) to enhance the OER has been already uncovered.8b Apparently, MnGa_4_ is a superior precursor evolving simultaneously three active disordered MnO_*x*_ mineral phases in alkaline media during electroconversion: δ‐MnO_2_ with Mn^III^/Mn^IV^, β‐MnOOH with Mn^III^, and α‐Mn_3_O_4_ with Mn^II^/Mn^III^ (Figure S64). Interestingly, β‐MnOOH is less stable and a reaction intermediate between the spinel‐to‐layer solid‐phase transition pathway.^26^ Therefore, it is expected that under OER conditions, α‐Mn_3_O_4_ is formed first and then transformed slowly into thermodynamically stable δ‐MnO_2_ via the β‐MnOOH intermediate (Scheme S1). Nevertheless, the combination of all three phases with profoundly exposed Mn^III^ boost the water oxidation and stability of MnGa_4_ enormously.

Based on the above compelling evidence, the higher activity and durability has been ascribed to (i) the structural flexibility of MnGa_4_ to undergo in situ electroconversion, (ii) formation of disordered and defect‐rich MnO_x_ phases of δ‐MnO_2_, β‐MnOOH, and α‐Mn_3_O_4_ with abundant Mn^III^ sites with an increased degree of Jahn–Teller distortion, (iii) effective stabilization of Mn^III^ in the active crystalline phases to facilitate O_2_ formation, (iv) a large electrochemically active surface with a higher density of active sites, and (v) fast electron transport from the catalyst surface and the electrode. Methodologically most important, this study highlights the suitability of well‐defined intermetallic precursors for the design of high‐performance catalysts with complex interface structure, bonding characteristics, and electronic properties, which is vital to increase the efficiency and long‐term stability of electrocatalysts.

## Conflict of interest

The authors declare no conflict of interest.

## Supporting information

As a service to our authors and readers, this journal provides supporting information supplied by the authors. Such materials are peer reviewed and may be re‐organized for online delivery, but are not copy‐edited or typeset. Technical support issues arising from supporting information (other than missing files) should be addressed to the authors.

Supplementary
