# Supplementary material for: Boosting Water Oxidation through In Situ Electroconversion of Manganese Gallide: An Intermetallic Precursor Approach
Source: Angew Chem Int Ed Engl. 2019 Oct 15;58(46):16569–74. doi: 10.1002/anie.201909904 (PMC6899514; doi:10.1002/anie.201909904)
Supplement: Supplementary file 1 — Supplementary [file ANIE-58-16569-s001.pdf]

Angewandte Chemie
Eine Zeitschrift der Gesellschaft Deutscher Chemiker

Supporting Information

Boosting Water Oxidation through In Situ Electroconversion of Manganese Gallide: An Intermetallic Precursor Approach

Prashanth W. Menezes[+], Carsten Walter[+], Jan Niklas Hausmann, Rodrigo Beltrán-Suito, Christopher Schlesiger, Sebastian Praetz, Valeriy Yu. Verchenko, Andrei V. Shevelkov, and Matthias Driess**

anie_201909904_sm_miscellaneous_information.pdf

Table of Contents

Contents	Page number
1. Materials	2
2. Instrumentations	2-3
3. X-ray photoelectron spectroscopy (XPS)	3
4. X-ray absorption spectroscopy (XAS)	3
5. Synthesis of the catalysts	3-4
6. Electrophoretic deposition on NF and FTO	4
7. Electrochemical measurements	4-5
8. PXRD of $MnGa_4$	6
9. SEM, EDX mapping and TEM images of as-prepared $MnGa_4$	7-16
10. FTIR analysis of as-prepared $MnGa_4$ and Mn reference	17
11. XANES analysis of as-prepared $MnGa_4$ and various Mn-based references	18
12. XPS analysis of as-prepared $MnGa_4$	19-20
13. PXRD of Mn reference	21
14. SEM, EDX mapping and TEM images of Mn reference	21-24
15. XPS analysis of Mn reference	25
16. OER activity study of $MnGa_4$ and Mn reference	26-33
17. PXRD analysis of $MnGa_4$ after electrochemistry	34
18. SEM, EDX mapping and TEM images of $MnGa_4$ after electrochemistry	35-38
19. FTIR analysis of $MnGa_4$ after electrochemistry	39
20. XANES analysis after OER CA	40
21. XPS analysis of $MnGa_4$ after electrochemistry	41-42
22. PXRD of $MnGa_4$ at various applied potentials	43
23. SEM, EDX mapping and TEM images of $MnGa_4$ after OER CV	44-46
24. Characterizations of $MnGa_4$ deposited on NF and after OER CA	47-52
25. Stability analysis of $MnGa_4$ in 1 M KOH	53
26. PXRD, SEM, EDX mapping, TEM images and FTIR analysis of Mn reference after electrochemistry	54-59
27. XPS analysis of Mn reference after electrochemistry	60
28. Structural information of $MnGa_4$ after electrochemistry	61
29. Transformation pathway of $MnGa_4$ to active OER catalysts	62
30. Comparison of OER activity with known materials of $MnGa_4$ and Mn references	63-64
31. References	65-66

Materials

Manganese (99.999%) and gallium (5N, pieces, 99.999%) metal and 1 M aqueous KOH and other reagents used in the synthetic procedures were obtained from Sigma Aldrich. The commercial RuO_2 (99%) and IrO_2 (99%), as well as selected manganese oxides (Mn, Mn_3O_4, Mn_2O_3, MnO_2), were purchased from Alfa Aesar. Nickel foam (NF), fluorine-doped tin oxide (FTO, resistivity 8−12 Ω/sq) and carbon cloth (CC) were obtained from Recemat BV, Sigma Aldrich and Cetech, respectively.

Instrumentations

Powder X-ray diffraction (PXRD) patterns were measured on a Bruker AXS D8 advanced automatic diffractometer equipped with a position-sensitive detector (PSD) and curved germanium (111) primary monochromator using Cu Kα radiation (λ = 1.5418 Å).

The inductively coupled plasma atomic emission spectroscopy (ICP-AES) was conducted on a Thermo Jarrell Ash Trace Scan analyzer. The materials were digested in aqua regia HCl: HNO_3 3:1 v/v (nitric acid, SUPRA-Qualität ROTIPURAN® Supra 69% and hydrochloric acid, SUPRA-Qualität ROTIPURAN® Supra 30%) and the average of three reproducible independent experiments has been presented (the electrolytes after electrochemistry were used directly). The digestion volume (2.5 mL) was diluted with Milli-Q water up to 15 mL. Calibration curves were prepared for both cobalt and phosphorus with concentrations between 1 mgL^{-1} and 100 mgL^{-1} from standard solutions (1000 mgL^{-1} Single-Element ICP-Standard Solution ROTI®STAR).

Fourier-transform infrared spectroscopy (FTIR) was studied using a BIORAD FTS 6000 FTIR spectrometer under attenuated total reflection (ATR) conditions. The data were recorded in the range of 500–4000 cm^{-1} with an average of 32 scans at 4 cm^{-1} resolution.

To gather information on the morphology, and the surface characteristics, the scanning electron microscopy (SEM) was conducted on an LEO DSM 982 microscope integrated with EDX (EDAX, Apollo XPP). Data handling and analyses were achieved with the software package EDAX.

The microstructure investigations of the materials were revealed by transmission electron microscopy (TEM) which was investigated on an FEI Tecnai G2 20 S-TWIN transmission electron microscope (FEI Company, Eindhoven, Netherlands) and JEOL 2100 electron microscope equipped with a LaB_6 source at 200 kV acceleration voltage. For the investigation of the films after electrocatalysis, the films were scraped from the electrode substrate and transferred onto a carbon-coated copper grid. EDX analyses were achieved with an EDAX r-TEM SUTW detector (Si (Li) detector), and the images were recorded with a GATAN MS794 P CCD camera. The SEM and TEM experiments were conducted partially at the Zentrum für Elektronenmikroskopie (ZELMI) of the TU Berlin.

Gas chromatography was used to calculate the Faradaic efficiency (FE) of oxygen evolution reaction (OER) that was performed in a closed (gas-tight) electrochemical cell. An Agilent 7890A gas chromatograph (GC) was used to determine the oxygen content in the headspace of the electrochemical cell. The GC was furnished with a carboxen-1000 column and a thermal conductivity detector (TCD). The carrier gas was argon (Ar).

A Signature Pro4 System measured the resistivity with Keithley 2400 source-measure unit (SP4-40045TBY) using a four-point probe resistivity technique. The spacing between tungsten carbide tips was 1.016 mm with a radius of 0.245 mm, and a spring pressure was 45 grams. The materials were electrophoretically deposited on electrodes to estimate the specific resistivity of each synthesized material, and the average results are presented.

X-ray photoelectron spectroscopy (XPS)

The XPS measurements were carried out on a Kratos Axis Ultra X-ray photoelectron spectrometer (Kratos Analytical Ltd., Manchester, U.K.) using an Al Kα monochromatic radiation source (1486.7 eV) with 90° takeoff angle (normal to analyzer). The vacuum pressure in the analyzing chamber was kept at 2×10^{-9} Torr. The XPS spectra were collected for C 1s, O 1s, Mn 2p, Mn 3p and Ga 3p levels with pass energy 20 eV and step 0.1 eV. The binding energies were calibrated relative to the C 1s peak energy position as 285.0 eV. Data analyses were carried out using Casa XPS (Casa Software Ltd.) and the Vision data processing program (Kratos Analytical Ltd.).

X-ray absorption spectroscopy (XAS)

Mn and Ga K-edge X-ray absorption near edge structure (XANES) spectra were collected using a von Hamos laboratory XAFS spectrometer,[1] which operates in transmission mode. As the source, an air-cooled microfocus X-ray tube with a power of 30 W, a spot size of 60 μm and molybdenum as the anode material was used. A cylindrically bent highly annealed pyrolytic graphite (HAPG) crystal with a size of 5 × 5 cm^2 and a bending radius of 15 cm was applied as a dispersive element and detection of the X-rays was accomplished using an indirectly detecting X-ray CCD camera of the type Andor Newton A-DY940P-FO-CSI with a spatial resolution of 50 μm and a size of 1 × 0.25 $inch^2$. With this setup, a spectral resolving power of E/ΔE = 2000 can be achieved in an energy range from 4 keV up to 12 keV. The ground powder samples of as-prepared $MnGa_4$ and Mn, as well as other reference materials MnO, MnO_2, and Mn_2O_3, have been applied on scotch tape and stacked to adopt the adequate thickness for the transmission mode measurements.[2] Also, the as-deposited films of $MnGa_4$ were also measured to have a fair comparison to that of ground powder. Similarly, the $MnGa_4$ films after the electrochemical OER experiments were measured, and the shifts in energy concerning as-prepared materials and reference materials were noted. The absorption spectra were normalized and analyzed using the XAS evaluation software ATHENA.47.[3]

Synthesis of the catalysts

Synthesis of intermetallic $MnGa_4$

The polycrystalline sample of $MnGa_4$ was prepared by annealing the stoichiometric mixture of Mn and Ga in an evacuated quartz ampule. The synthetic conditions were chosen based on the reported phase diagram.[4] The ampule was heated in a programmable furnace to 900 ˚C, annealed at this temperature for four days to ensure homogeneity of the mixture, cooled at the rate of 20 ˚C/h to 380 ˚C and annealed at 380 ˚C for ten days. Then, the material was thoroughly ground and annealed at 380 ˚C for another ten days to produce pure $MnGa_4$ phase.

Synthesis of Mn_2O_3-HT[5]

0.5 g of bis(cyclopentadienyl)manganese(II) was weighed in a glove box and carefully transferred under protective atmosphere into argon flushed tube furnace. The furnace was then flushed with argon for 2 h and then switched to oxygen atmosphere during annealing. The temperature was increased with 6 ˚C/min to 700 °C and maintained at that temperature for 12 h followed by cooling down naturally to room temperature. The product consisted of small particles and dark grey colored.

Synthesis of Mn_2O_3-SSP[6]

The preparation of Mn_2O_3-SSP was carried out in two steps. In the first step, micro-emulsions containing cetyltrimethylammonium bromide (CTAB, 2.0 g) as a surfactant, 1-hexanol (20 mL) as co-surfactant and hexane (35 ml) as the lipophilic phase were prepared and were mixed separately with an aqueous solution of 0.1 M manganese acetate and ammonium oxalate. Both micro-emulsions were mixed slowly and stirred overnight. The white precipitate then obtained was centrifuged and washed with 1:1 mixture of chloroform and methanol (200 mL) and subsequently dried at 60 ˚C for 12 hours. In the next step, the manganese oxalate precursor was heated in dry synthetic air (20% O_2, 80% N_2) at 400 ˚C for 8 hours (2 ˚C/min) to form monophasic Mn_2O_3.

Electrophoretic deposition (EPD) on NF, FTO, and CC

The investigated materials were deposited on both, NF, FTO and CC, electrophoretically, by applying a potential difference of 10 V in a mixture of iodine and acetone on a 1 × 1 cm^2 area. The detailed mechanism involving electrophoretic deposition has been described elsewhere.[7] The electric charge on the catalyst in acetone is insufficient for EPD as very small amounts of free ions exist in acetone, and therefore, large potentials are required for EPD.[7] When iodine is used as the dispersant, it can react with acetone through the keto-enol tautomerism to produce protons as per the following equation.

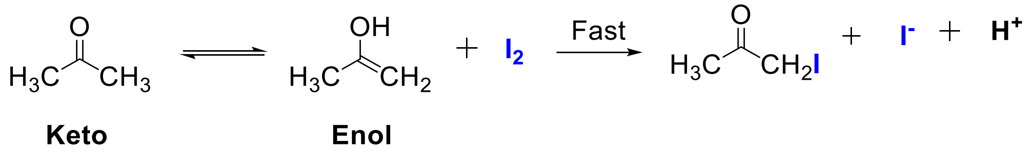

Thus formed protons are adsorbed on the surface of the suspended particles by making them positively charged. The applied electric field induces the positively charged particles to migrate towards and deposit on the cathode.[8] In order to have a better deposition on the electrode substrates, the large $MnGa_4$ crystals were grounded for 15 min to reduce that reduced the particle size. Typically, 30 mg of the catalyst powder was suspended in 10 ml acetone, and 3 mg of iodine was then added. This solution was agitated in an ultrasonic bath for 30 min. Before EPD, the empty electrodes were weighed using an analytical balance, and the weights were noted. The EPD was conducted at various potentials different time intervals, and the thin uniform films were only achieved by applying a potential at -10 V for 2 minutes with stirring the solution continuously at room temperature. After each EPD, the increase in weight of the electrodes was monitored carefully. The catalyst loading on each NF, CC, and FTO was ~2,

~1.1 and ~0.7 $mgcm^{-2}$, respectively. The mass loading was reproducible within the margins of an experimental error (±0.05 mg).

Electrochemical measurements

A typical electrocatalytic run was carried out in a standard three-electrode (working, counter and reference) electrochemical cell in 1 M aqueous KOH with a potentiostat (SP-200, BioLogic Science Instruments) controlled by the EC-Lab v10.20 software package. The electrodes (NF/FTO/CC) with samples deposited served as the working electrodes, Pt wire (0.5 mm diameter × 230 mm length; A-002234, BioLogic) as a counter and Hg/HgO as the reference electrode (CH Instruments, Inc.). Cyclic voltammetry (CV) and linear sweep voltammetry (LSV) were carried out with an applied iR compensation of 85%. The potentials presented in this work were referenced to the reversible hydrogen electrode (RHE) through calibration, and in 1 M aqueous KOH, E(RHE) = E(Hg/HgO) + 0.098 V + (0.059 × pH) V. The chronoamperometric measurements were performed in 1 M aqueous KOH at selected constant potentials with respect to RHE. The Tafel slope was calculated according to Tafel equation $\eta = b\log j + a$, where η is overpotential (V), j is the current density (mA cm^{-2}), and b is the Tafel slope (mV dec^{-1}).[9]

The electrochemically active surface area (ECSA) of both $MnGa_4$ and Mn was obtained by determining their double layer capacitances (C_{dl}) from the CV (cycled between 0.925 and 1.025 V vs. RHE) at a potential range, where no apparent faradaic process occurred.[10]The anodic charging currents measured at 0.975 V vs. RHE were plotted as a function of the scan rate and from the slope, and the double layer capacitance C_{dl} was attained.[8] The ECSA of the catalysts were then calculated using the equation

$$ECSA = C_{dl} / C_s$$

where C_s can be defined as the specific capacitance of the material per unit area under identical electrolyte conditions and a specific capacitance C_s of 1.7 mF cm^{-2} was utilized for NF substrate in 1 M aqueous KOH solution which is based on the literature reported values.[7d,11]

One of the important measurements, the electrochemical impedance spectroscopy (EIS) were recorded at 1.5 V vs. RHE to obtain the Nyquist plots.[12] The amplitude of the sinusoidal wave was examined in a frequency range of 100 kHz to 1 mHz. All impedance spectra were fitted using an equivalent RC circuit model. The charge transfer resistance (R_{ct}) was then obtained from the diameter of the semicircle in the Nyquist plots.

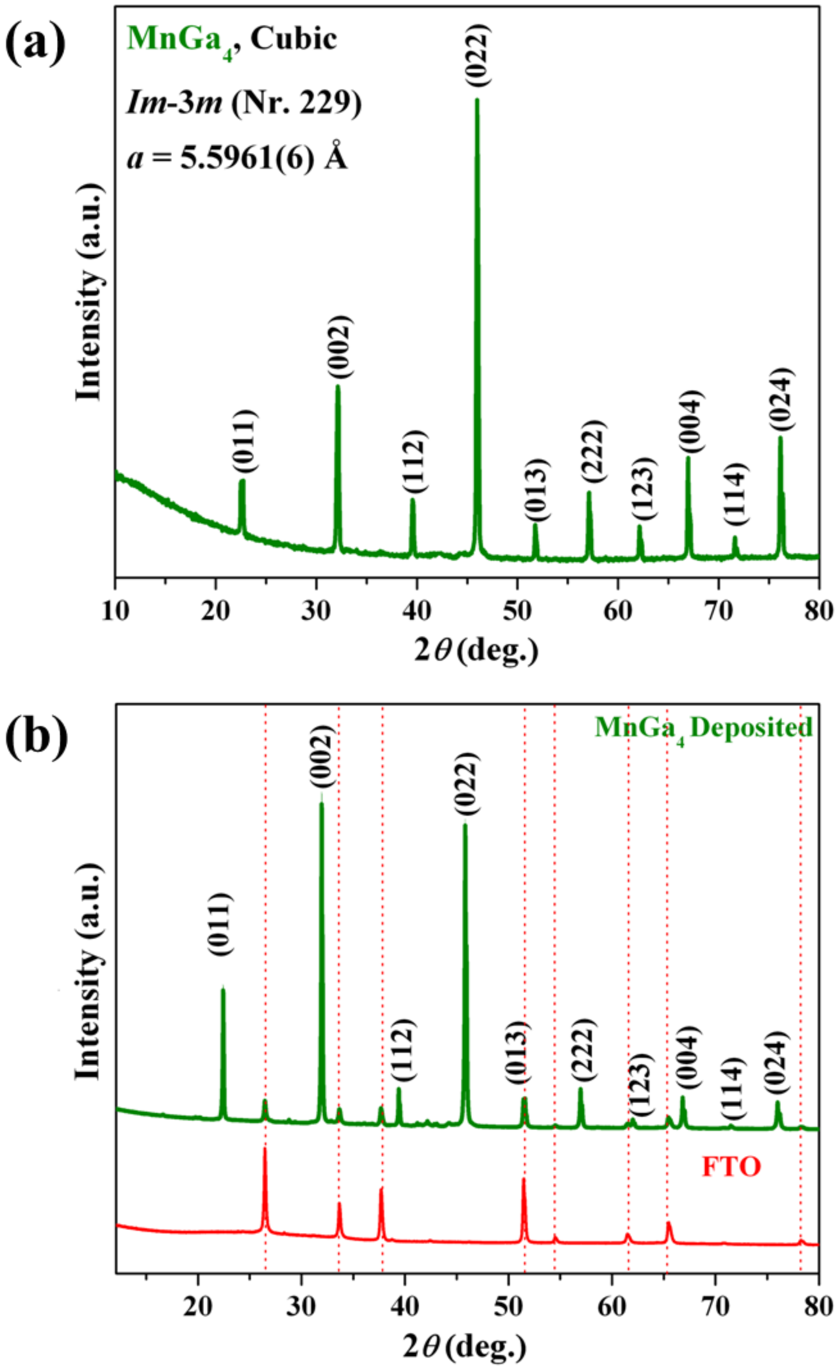

Figure S1. The PXRD pattern of (a) as-synthesized and (b) electrophoretically deposited intermetallic $MnGa_4$ film. The powder pattern of $MnGa_4$ matches well with reported cubic $MnGa_4$ (*Im*-3*m*, Nr. 229) with lattice parameter *a* = 5.5961(6) Å, *V* = 175.06(2) Å^3 and *Z* = 2.[4,13] The phase confirmation and elemental composition were further identified by HRTEM, EDX, ICP-AES, and XPS studies. The FTO powder pattern is shown in red.

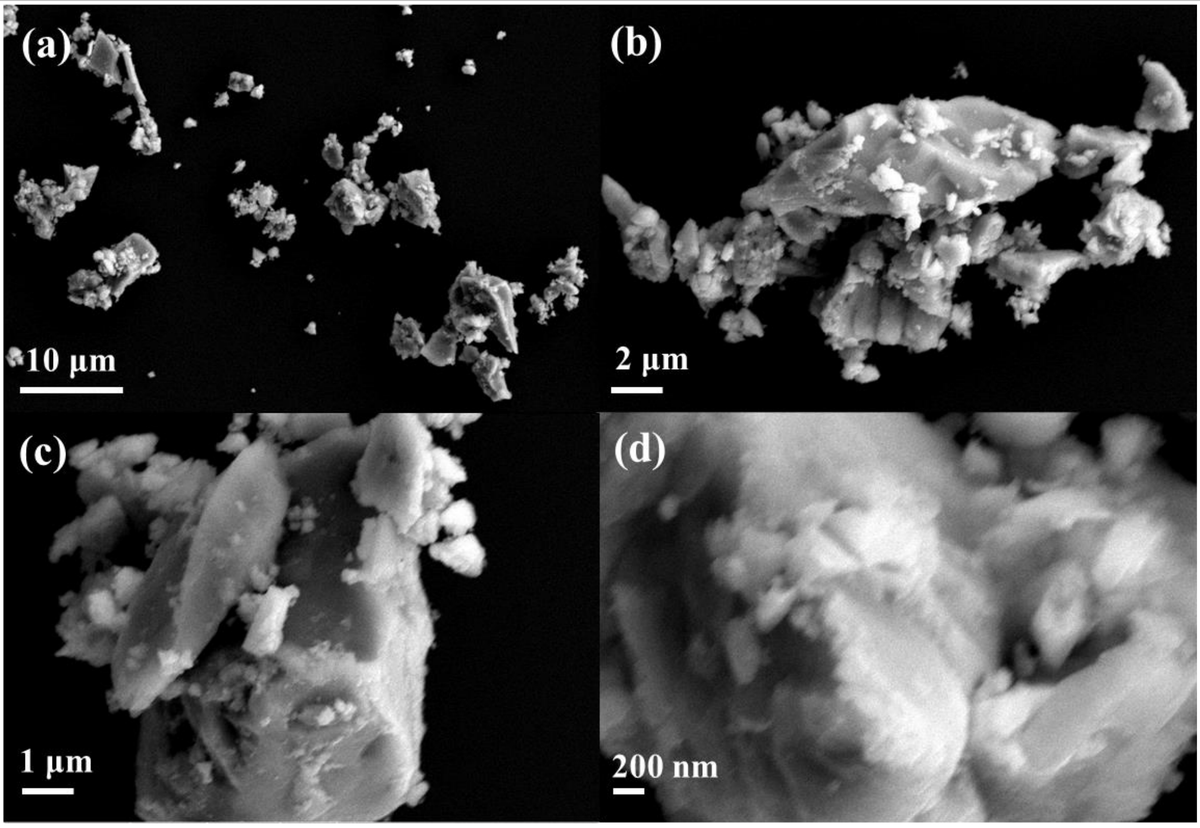

Figure S2. SEM images (a-d) of $MnGa_4$ with different magnifications. The morphology of $MnGa_4$ showed irregularly shaped particles with varying size.

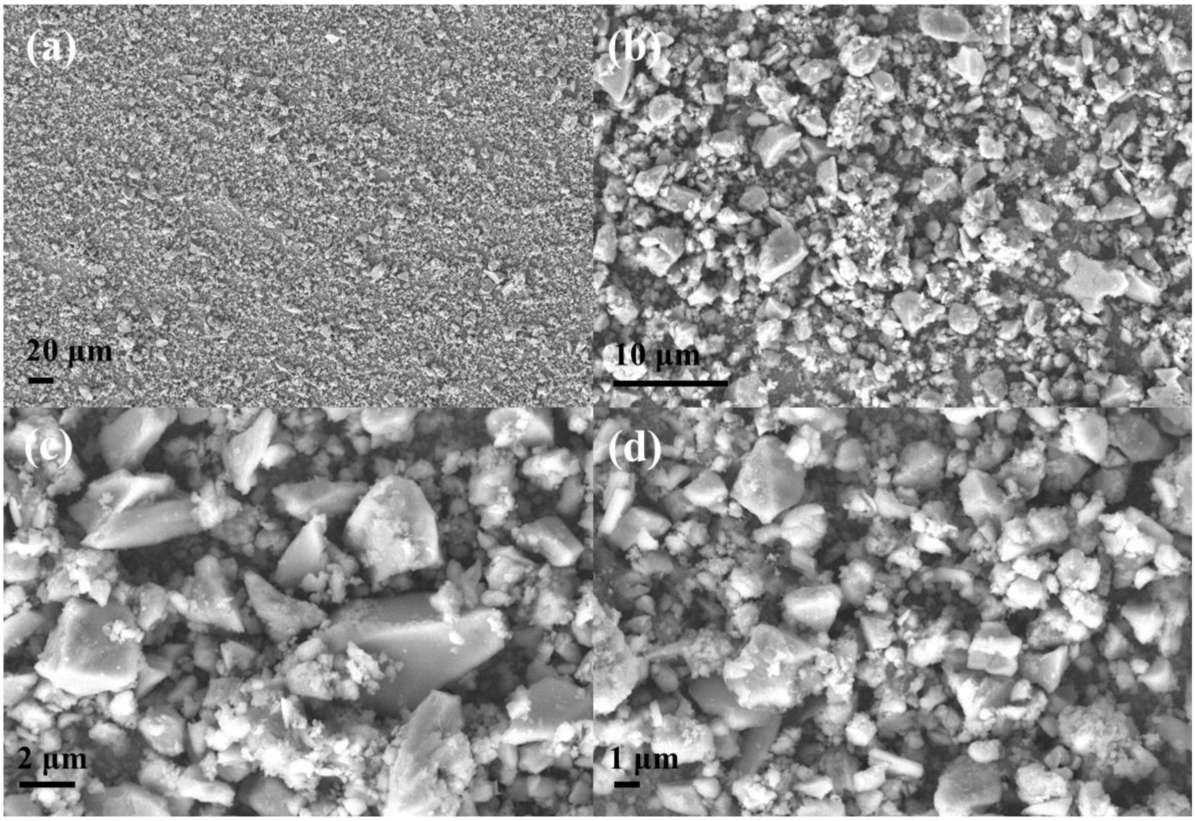

Figure S3. SEM images (a-d) of electrophoretically deposited $MnGa_4$ film with different magnifications. The morphology of $MnGa_4$ was consistent with the as-synthesized $MnGa_4$.

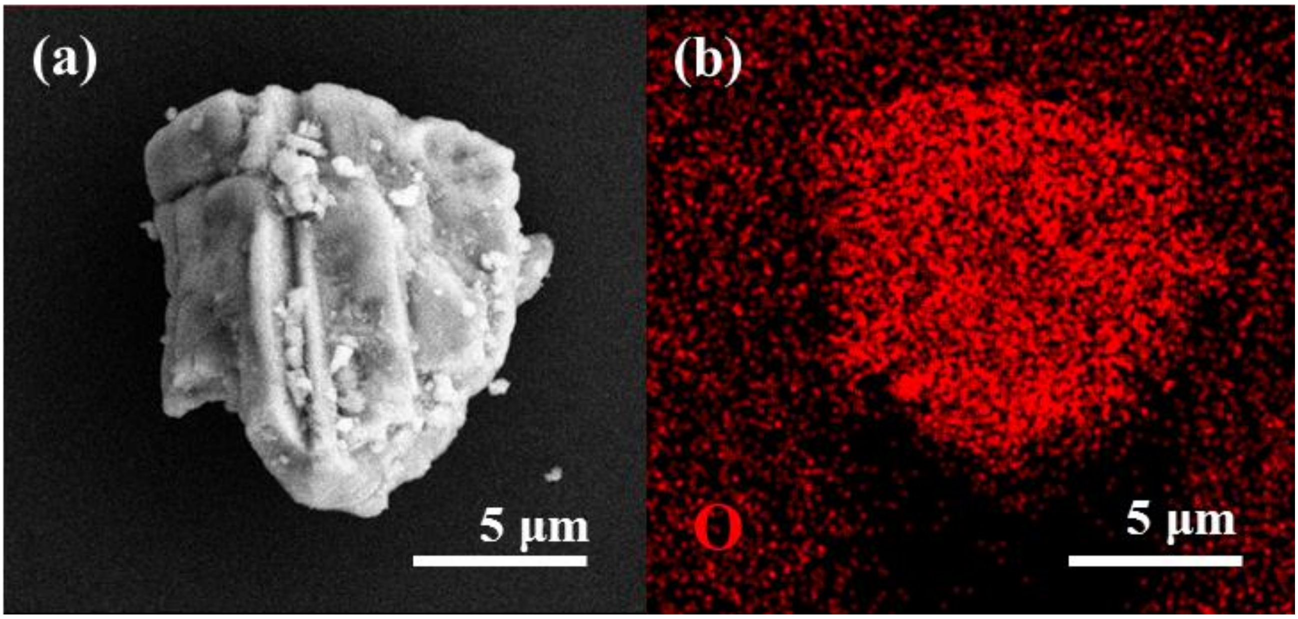

Figure S4. The SEM image (a) and the elemental mapping of oxygen (b) on the surface of $MnGa_4$ crystals (please refer to Figure 1 and Table S2).

Table S1. The distribution of the elements by atomic % in the as-prepared $MnGa_4$ crystals, grounded polycrystalline $MnGa_4$, $MnGa_4$ films after OER, Mn metal powder and Mn films after OER. The EDX results were collected at several points of the measurement, and the average value is presented.

	Mn(atomic %)	Ga (atomic %)	O (atomic %)
$MnGa_4$ Crystals	20.33	79.67	<1
$MnGa_4$ Grounded powder	20.19	79.81	<1
$MnGa_4$ deposited	20.45	79.55	<1
$MnGa_4$ OER CA	36.85	5.26	57.89
Mn	99.41	-	0.59
Mn OER CA	73.03	-	26.97

Table S2. Determination of manganese and gallium ratio in $MnGa_4$ was obtained by ICP-AES, EDX, and XPS analysis. Each experiment was conducted thrice independently, and the average value is presented.

	Mn:Ga (Theo.)	Mn:Ga (EDX)	Mn:Ga (ICP-AES)	Mn:Ga (XPS)
$MnGa_4$ Powder	1:4	1:4.06	1:4.01	~1:4
$MnGa_4$ deposited	1:4	1.4.01	-	~1:4
$MnGa_4$ OER CV		1:3.38	-	-
$MnGa_4$ OER CA	-	1:0.14	1:0.08	1:0

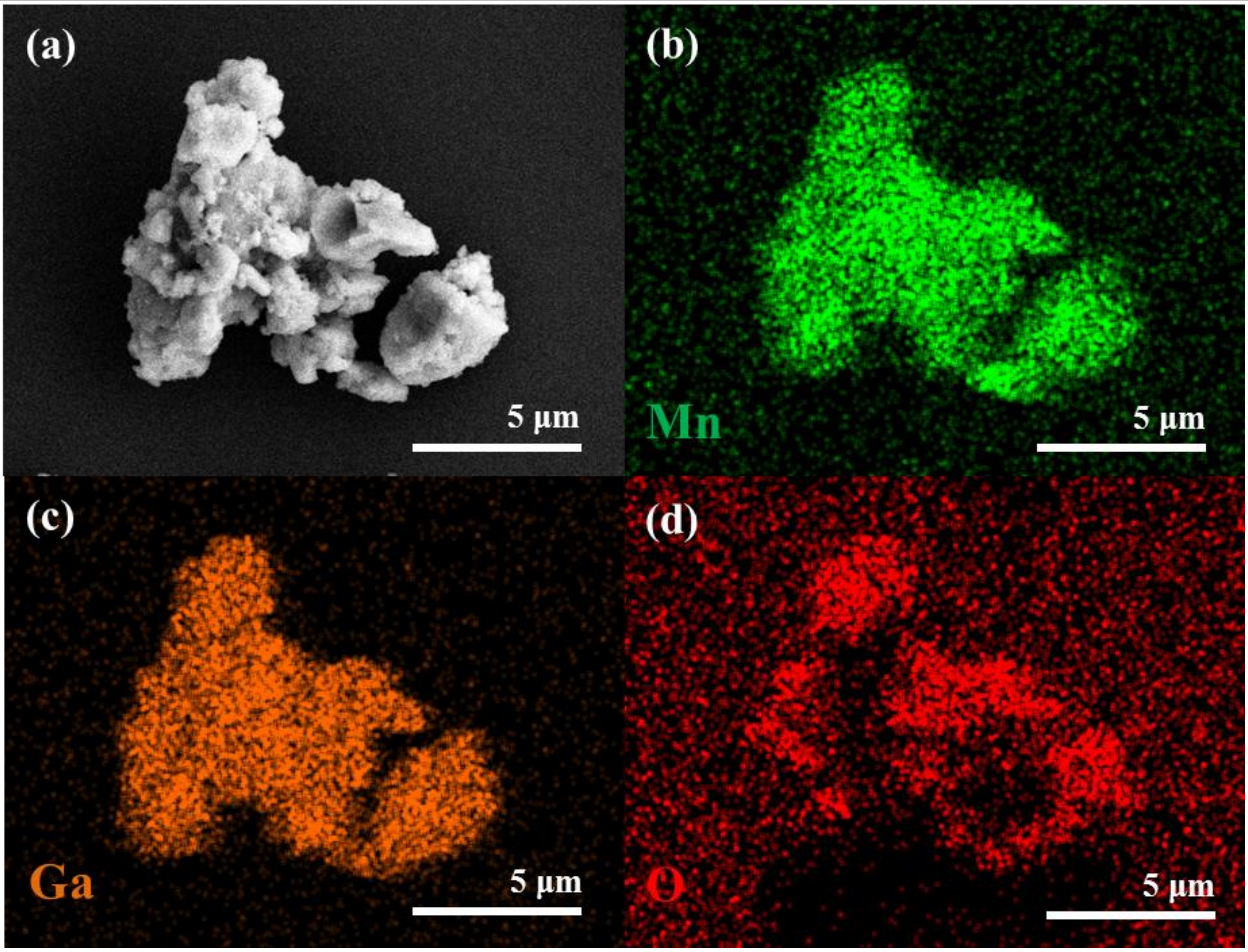

Figure S5. The SEM image (a) and the EDX mapping (b-d) which was carried out on the grounded crystals of $MnGa_4$ to ensure the phase purity of the material. Similar to crystals, the grounded $MnGa_4$ particles exhibited a homogenous distribution of manganese and gallium within the structure without any oxygen (<1) content. Detailed atomic % of the distribution of the elements obtained by EDX is listed in Table S1.

Table S3. Results of the four-point probe resistivity measurement on FTO of the $MnGa_4$ catalysts which show the highly metallic character of $MnGa_4$ and are in line with the literature reports. Besides, the resistivity of Mn was also measured to have a fair comparison.[4,13]

Sample	Resistivity (Ω/sq)
FTO	7.26
$MnGa_4$	6.94
Mn	8.16

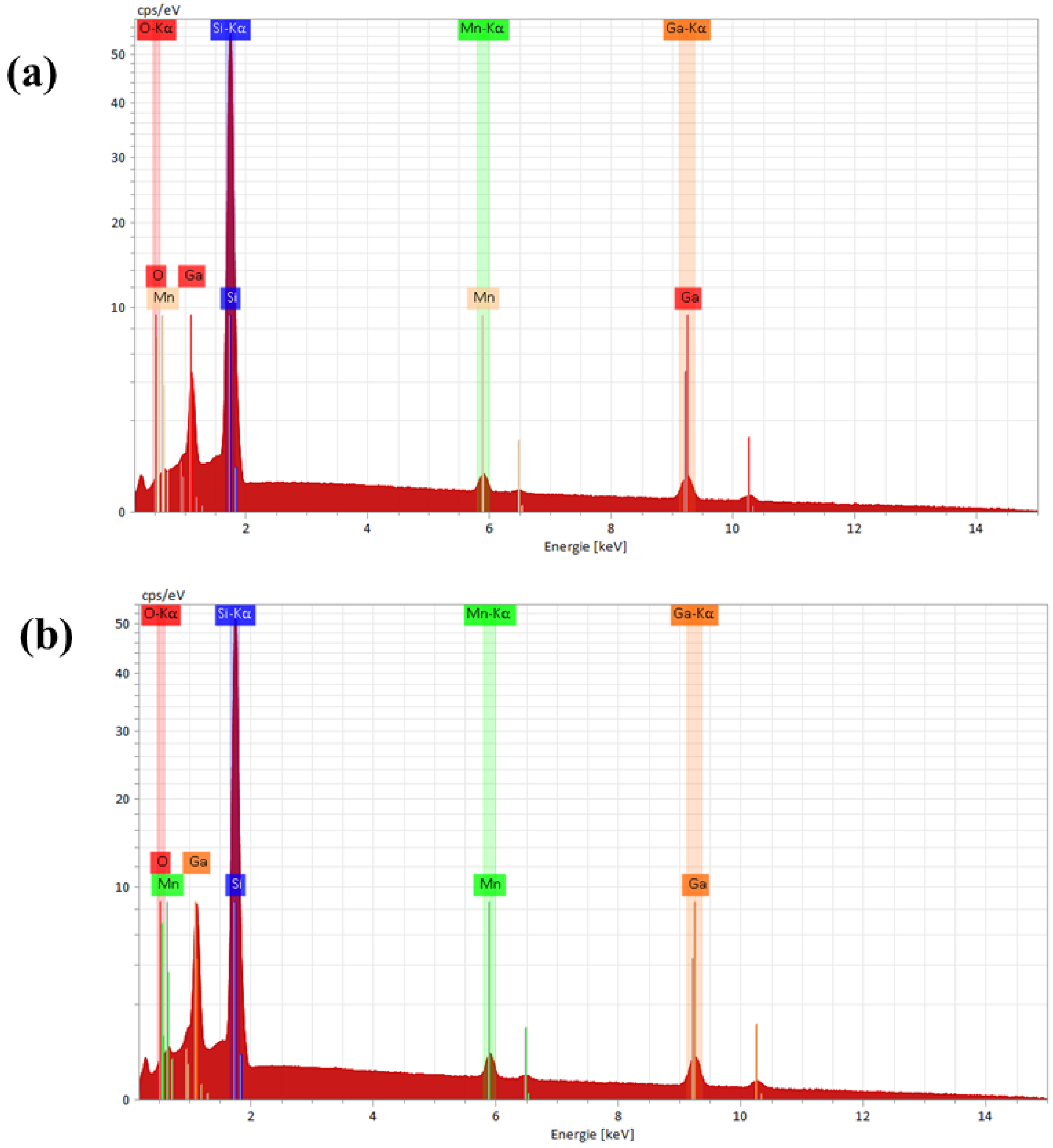

Figure S6. The EDX mapping spectrum of (a) $MnGa_4$ crystals, (b) grounded polycrystalline $MnGa_4$ samples.

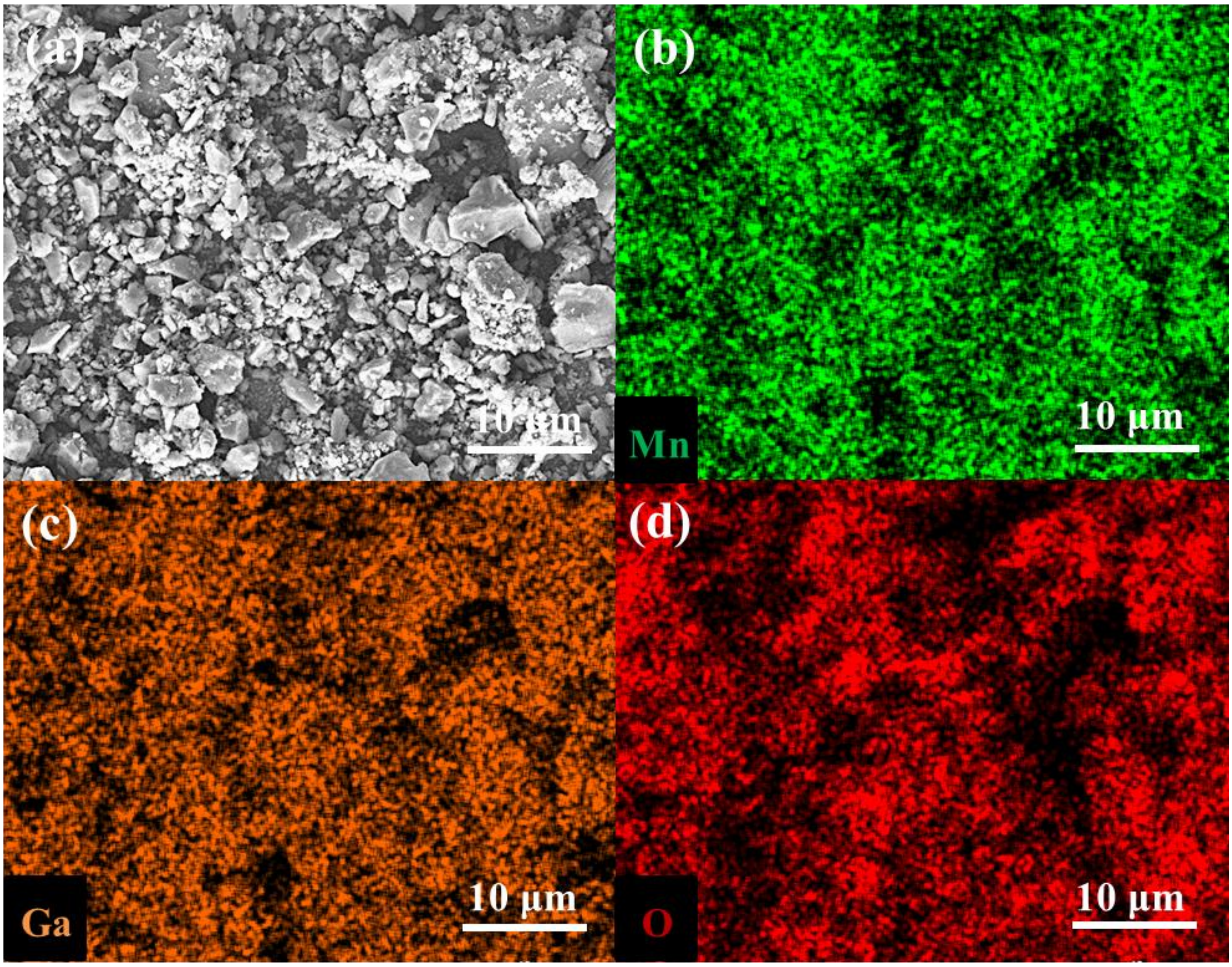

Figure S7. The SEM image (a) and the EDX mapping (b-d) which was carried out on the electrophoretically deposited $MnGa_4$ film showing a homogenous distribution of manganese and gallium within the structure without any oxygen (<1) content. Detailed atomic % of the distribution of the elements obtained by EDX is listed in Table S1.

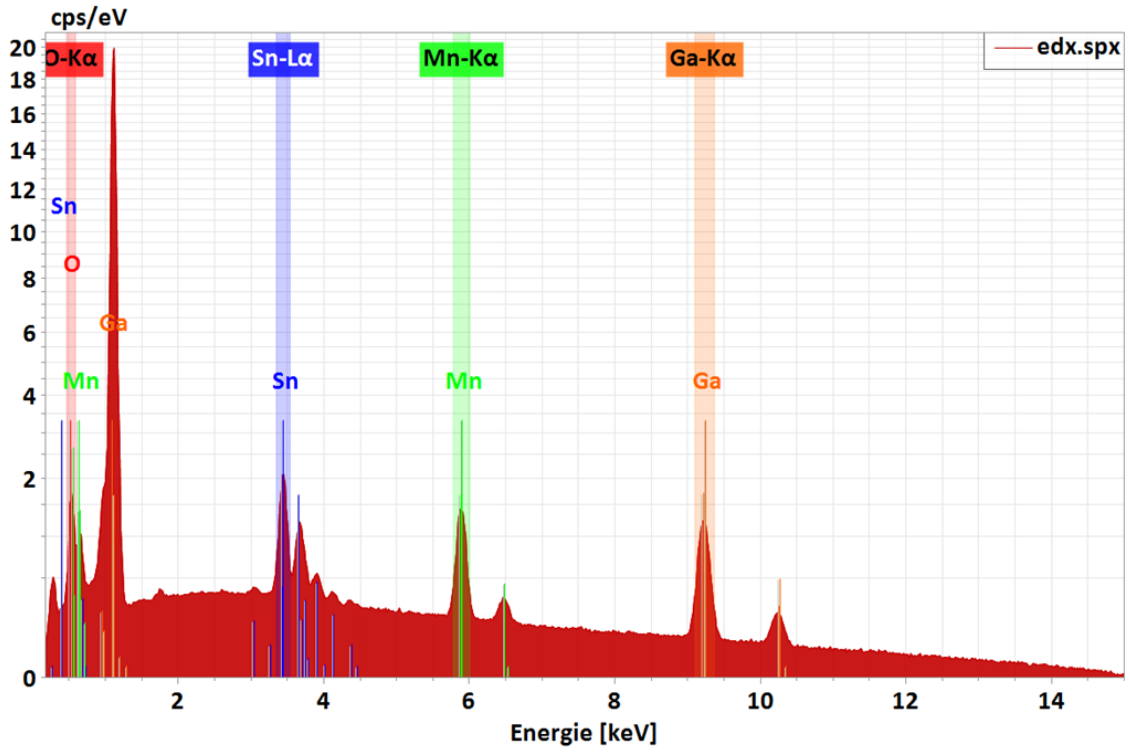

Figure S8. The EDX mapping spectrum of electrophoretically deposited $MnGa_4$ film**.**

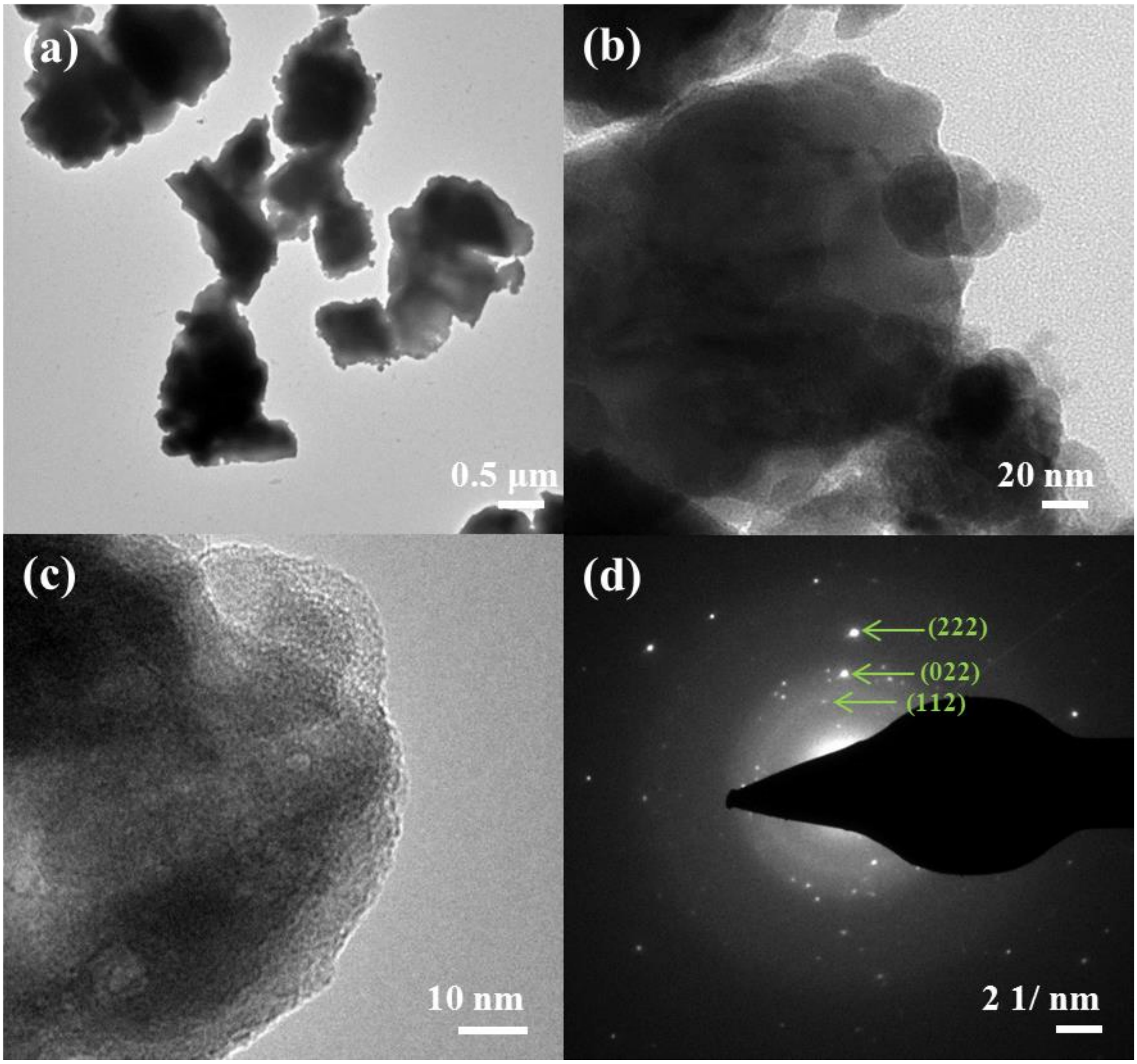

Figure S9. The TEM image (a) and High-resolution TEM (HR-TEM) images (b, c) of $MnGa_4$ reveal irregularly shaped particles with varying sizes. The selected area diffraction pattern (SAED, d) indicated that the particles were very crystalline. The SAED pattern displayed diffractions rings corresponding to the crystallographic planes (112), (022) and (222) at d = 0.23, 0.2 and 0.16 nm which is also consistent with PXRD pattern confirming the phase purity of the product.[4,13]

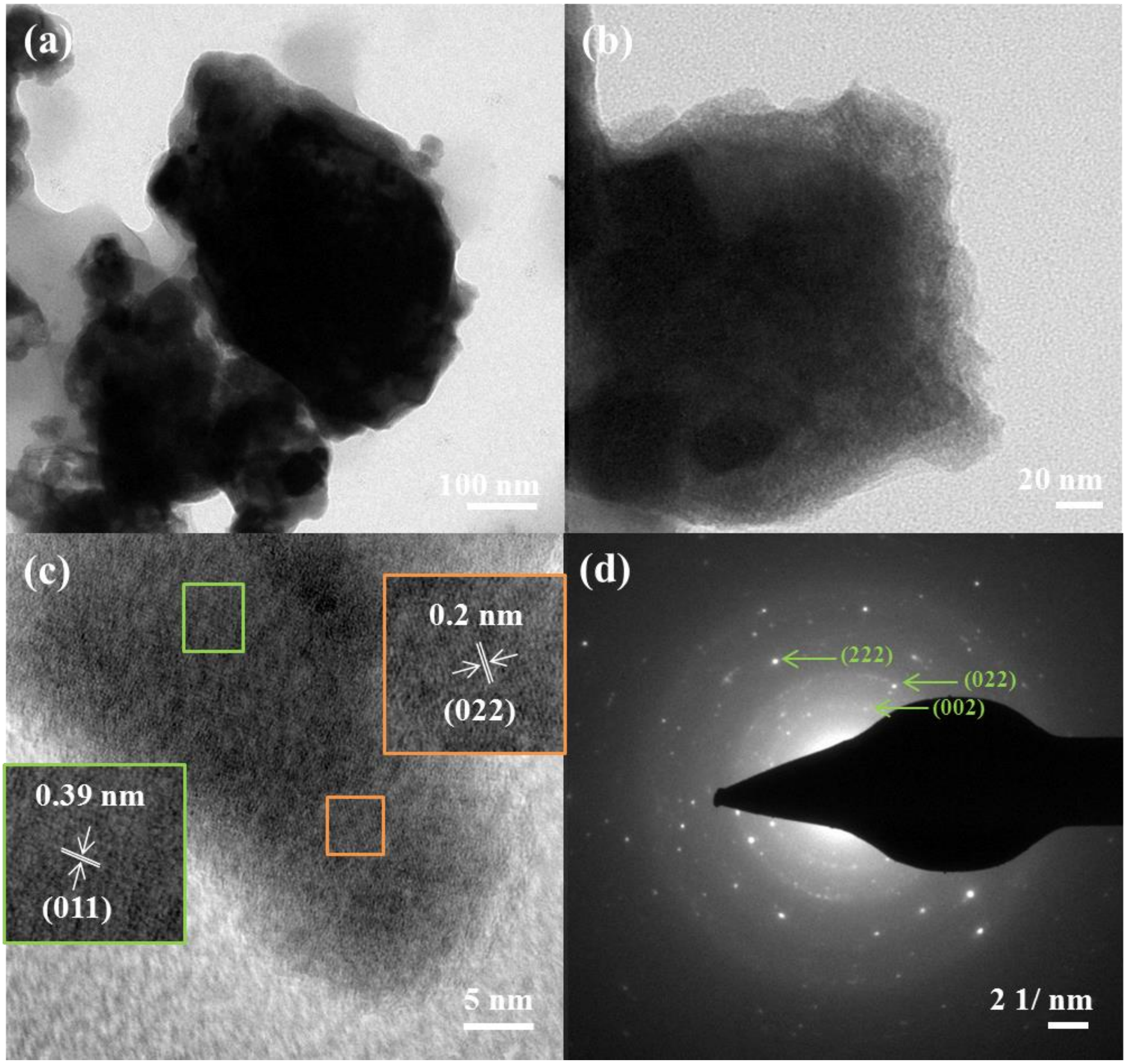

Figure S10. The TEM image (a) and HR-TEM images (b, c) of electrophoretically deposited $MnGa_4$ film. The selected area diffraction pattern (SAED, d) indicated that the particles were very crystalline. The SAED pattern displayed diffractions rings corresponding to the crystallographic planes (002), (022) and (222) at d = 0.28, 0.2 and 0.16 nm which is also consistent with PXRD pattern confirming the phase purity of the product.[4,13]

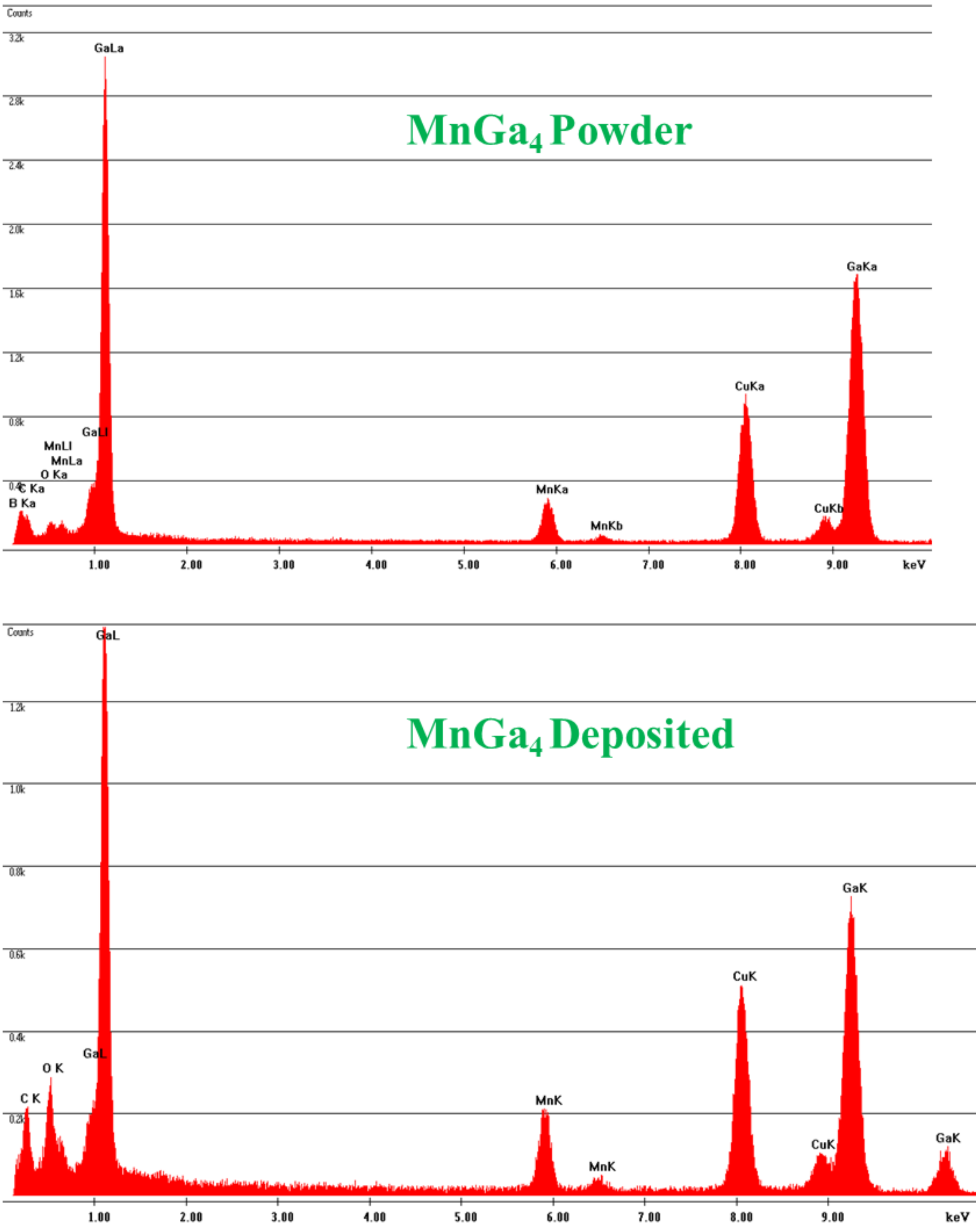

Figure S11. EDX analysis of as-synthesized and electrophoretically deposited $MnGa_4$ film, confirming the presence of Mn and Ga. The peaks for copper can be unambiguously correlated to the TEM grid (carbon film on 300 mesh Cu-grid).

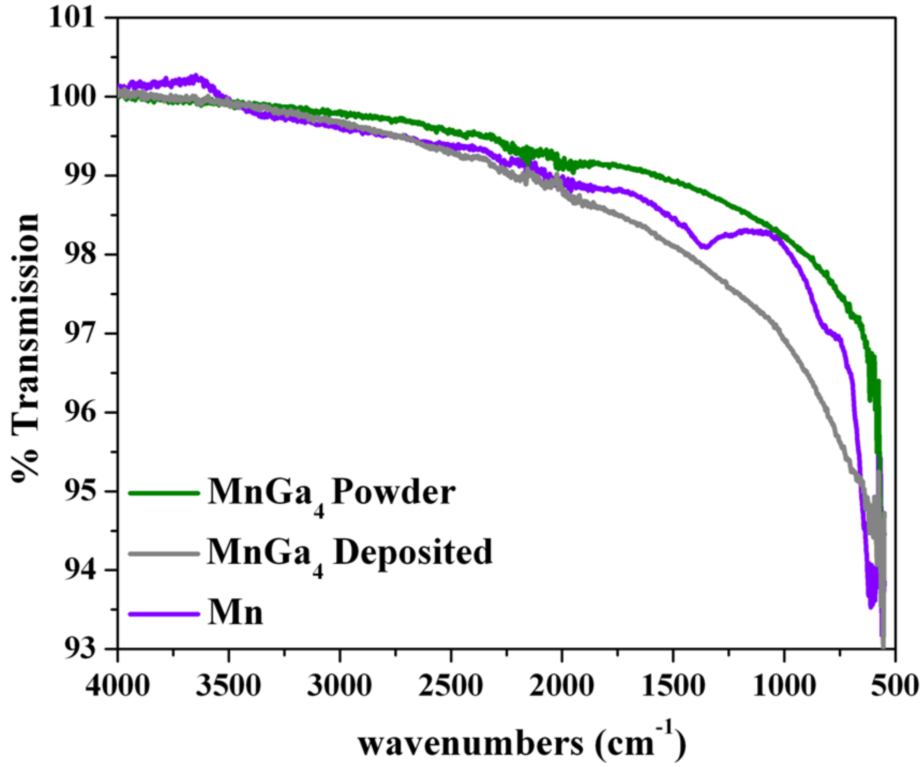

Figure S12. The FTIR spectra of powder and electrophoretically deposited $MnGa_4$ film as well as Mn powder. Apart from surface passivation, no indication of massive hydroxylation was attained. The band between 500-700 cm^{-1} could be ascribed to vibrations for Mn–Ga.

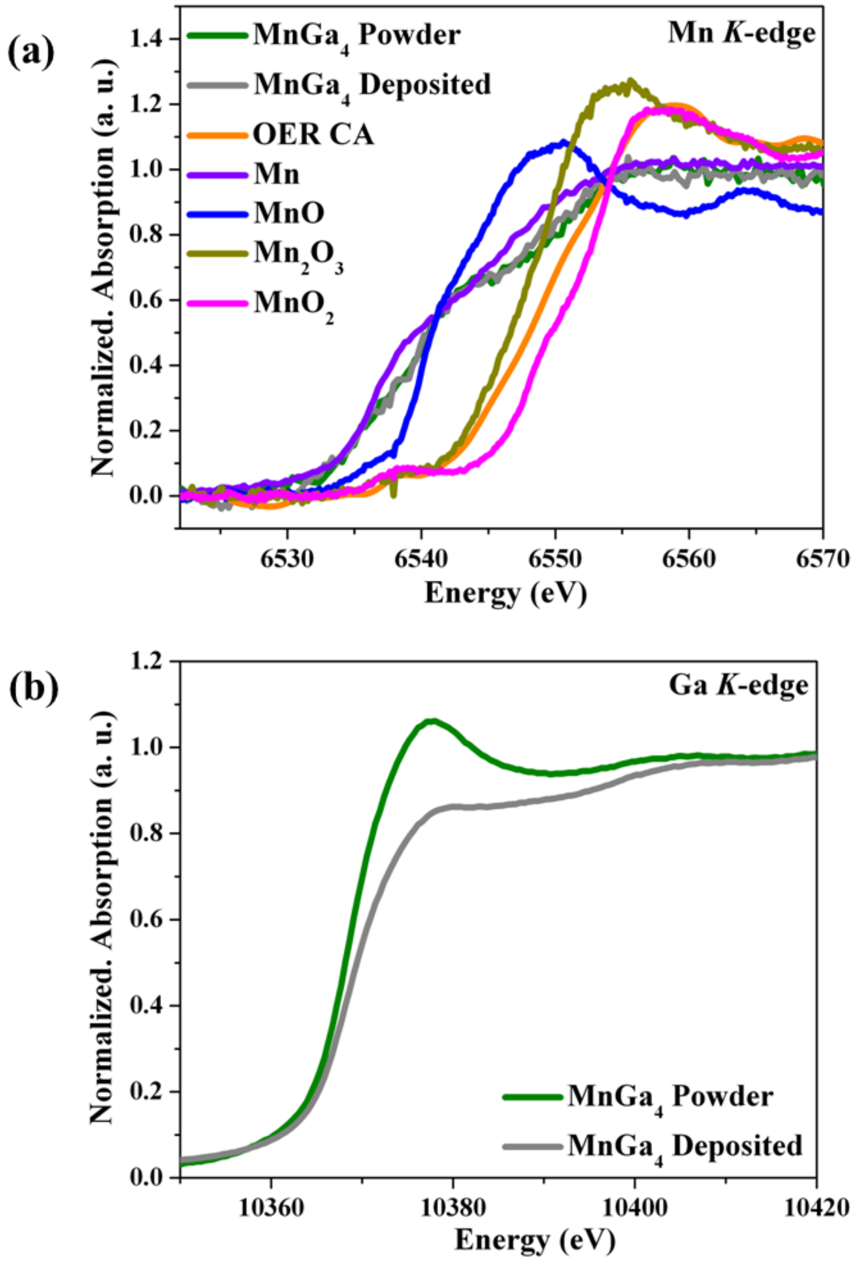

Figure S13. The XANES spectra of powder and electrophoretically deposited $MnGa_4$ film at (a) Mn *K*-edge and (b) Ga *K*-edge measured in the air under ambient pressure. To deduce a clear comparison, the spectra of Mn, MnO, Mn_2O_3, and MnO_2 with different oxidation states measured in similar conditions have also been included as references. The Mn *K*-edge, corresponding to a dipole transition of 1s core electron to 4p-like unoccupied states, serves as a qualitative spectroscopic fingerprint to support the identification of the manganese species present in $MnGa_4$. The shape of the Mn *K*-edge XANES in $MnGa_4$ overlaps with the one of metallic Mn (violet) with a slight shift suggesting the most of the Mn possesses metallic character.[2,14] Similarly, the Ga *K*-edge XANES also exhibits spectra closely resembling with that of Ga, as reported in the literature.[15]

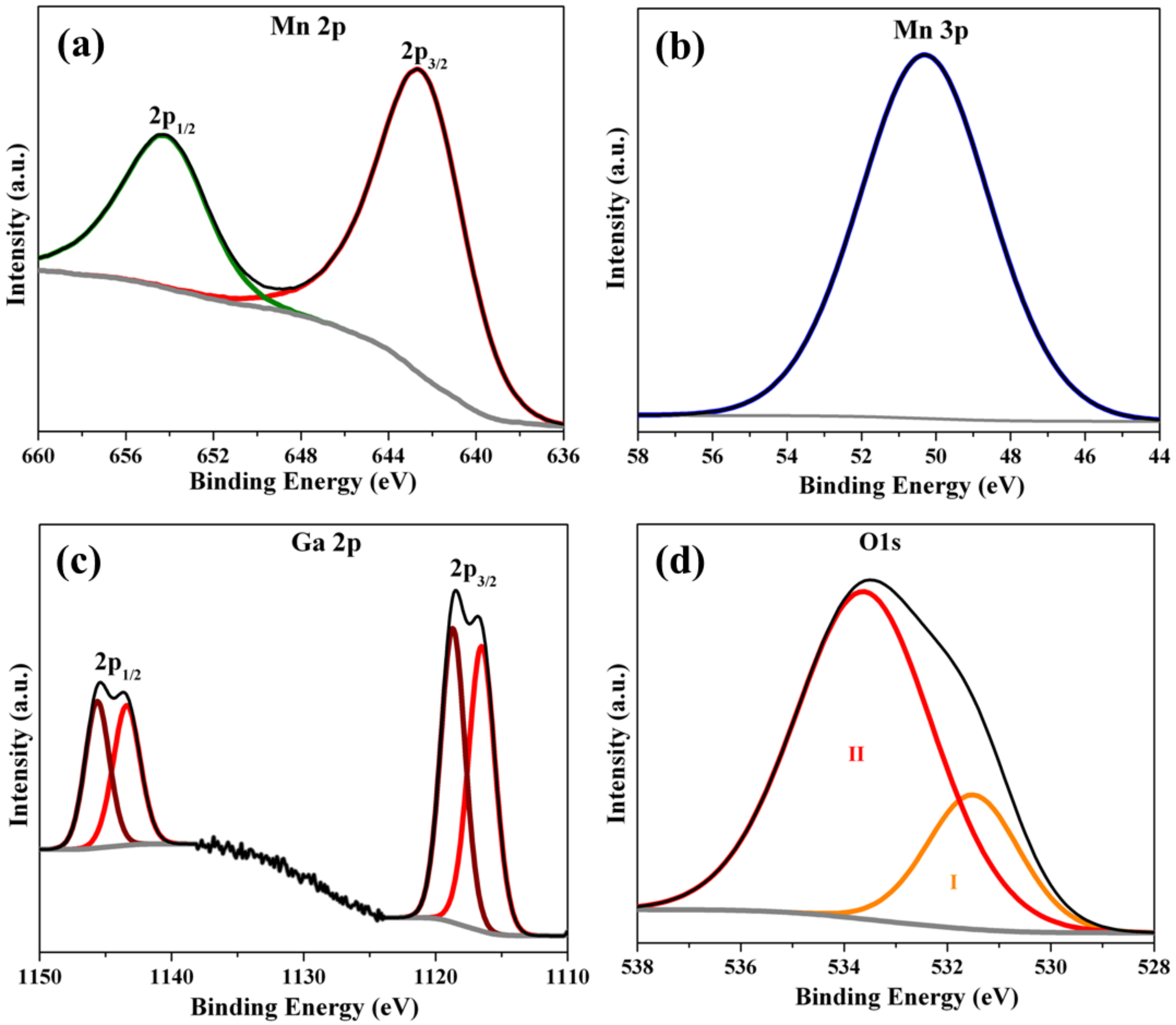

Figure S14. The high-resolution deconvoluted (a) Mn 2p, (b) Mn 3p, (c) Ga 2p and (d) O 1s XPS spectra of deposited $MnGa_4$ films. The Mn $2p_{3/2}$ and Mn $2p_{1/2}$ spectrum exhibit sharp peaks at the binding energy of 642.3 eV and 654.2 eV (a) that are very close to the oxidation state of Mn^{IV} (MnO_2).[16] Similarly, in the case of manganese-based materials, the oxidation state of Mn can also be deduced from their Mn 3p spectra, and it is typical to measure ~47.5 eV for Mn^{II}, ~48.5 eV for Mn^{III} and ~50 eV for Mn^{IV}.[16c,17] Therefore, the resulted binding energy value of 50.2 eV in Mn 3p spectra could be assigned to Mn^{IV} (b). In the case of Ga 2p, the binding energy of 1116.4 eV attained for Ga 2p3/2 is very similar to the binding energy of elemental Ga (1116.4 eV).[18] The second peak observed at the binding energy of 1118.2 eV could be corroborated with Ga bonded to oxo-species, confirming the unavoidable surface oxidation of the intermetallic phase, which is a common phenomenon and often observed in chalcogenides, pnictogenide or intermetallic compounds.[5,7d,19] The surface oxidation was also confirmed from O1s XPS spectra where two deconvoluted peaks (I and II) at 531.3 eV and 533.8 eV can directly be ascribed to surface hydroxylation and water adsorbed onto the surface.[5,7d,19b-e,20]

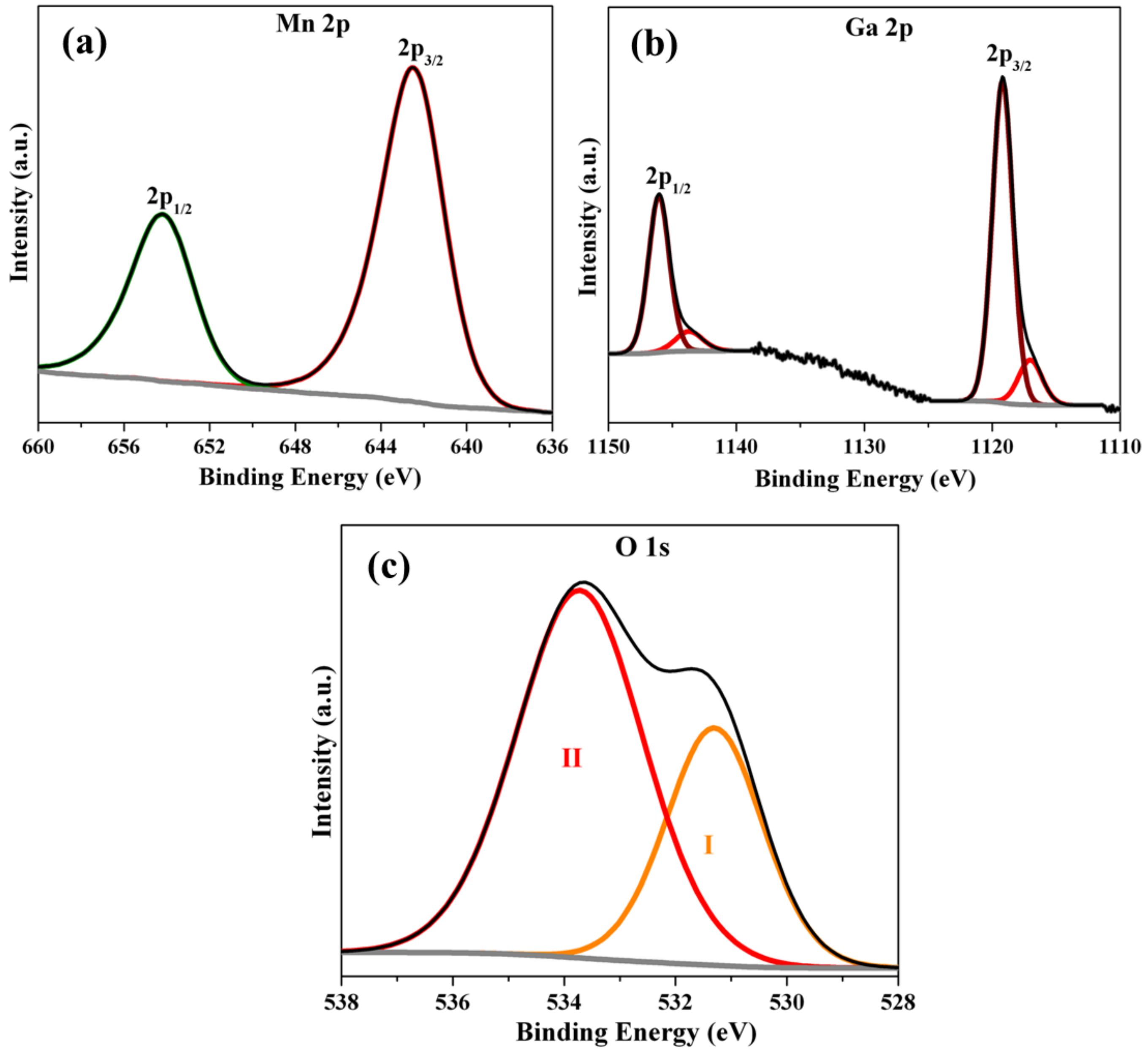

Figure S15. The high-resolution deconvoluted (a) Mn 2p, (b) Ga 2p and (d) O 1s XPS spectra of $MnGa_4$ powder. For a detailed deconvoluted description, please refer to Figure S14. The XPS of both electrophoretically deposited film and powder $MnGa_4$ materials were identical except slight surface oxidation of Ga in the powdered material.

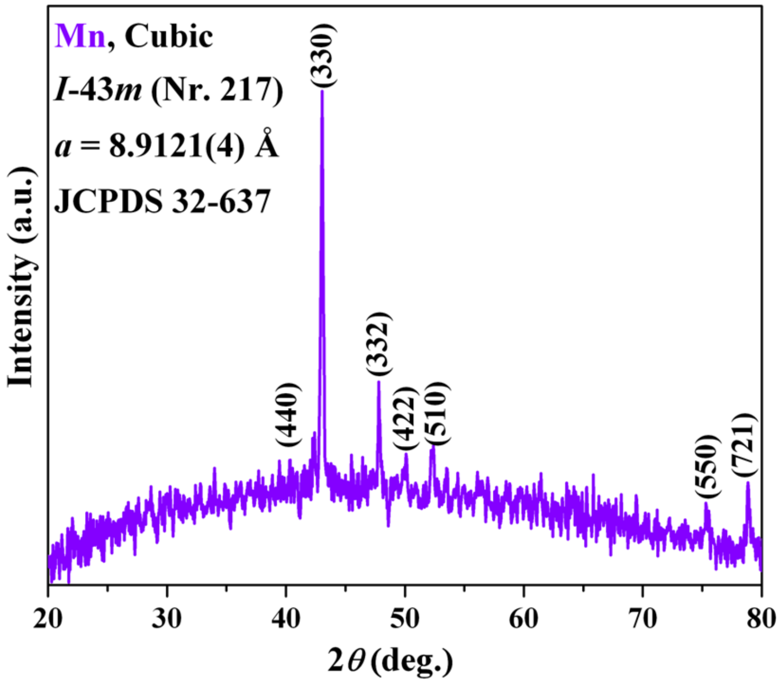

Figure S16. The PXRD pattern of metallic Mn. The powder pattern of Mn matches well with reported cubic Mn (*I*-43*m*, Nr. 217) with lattice parameter *a* = 8.9121(4) Å, *V* = 353.92(2) Å^3 and *Z* = 58. The HRTEM and SAED further identified the phase confirmation.

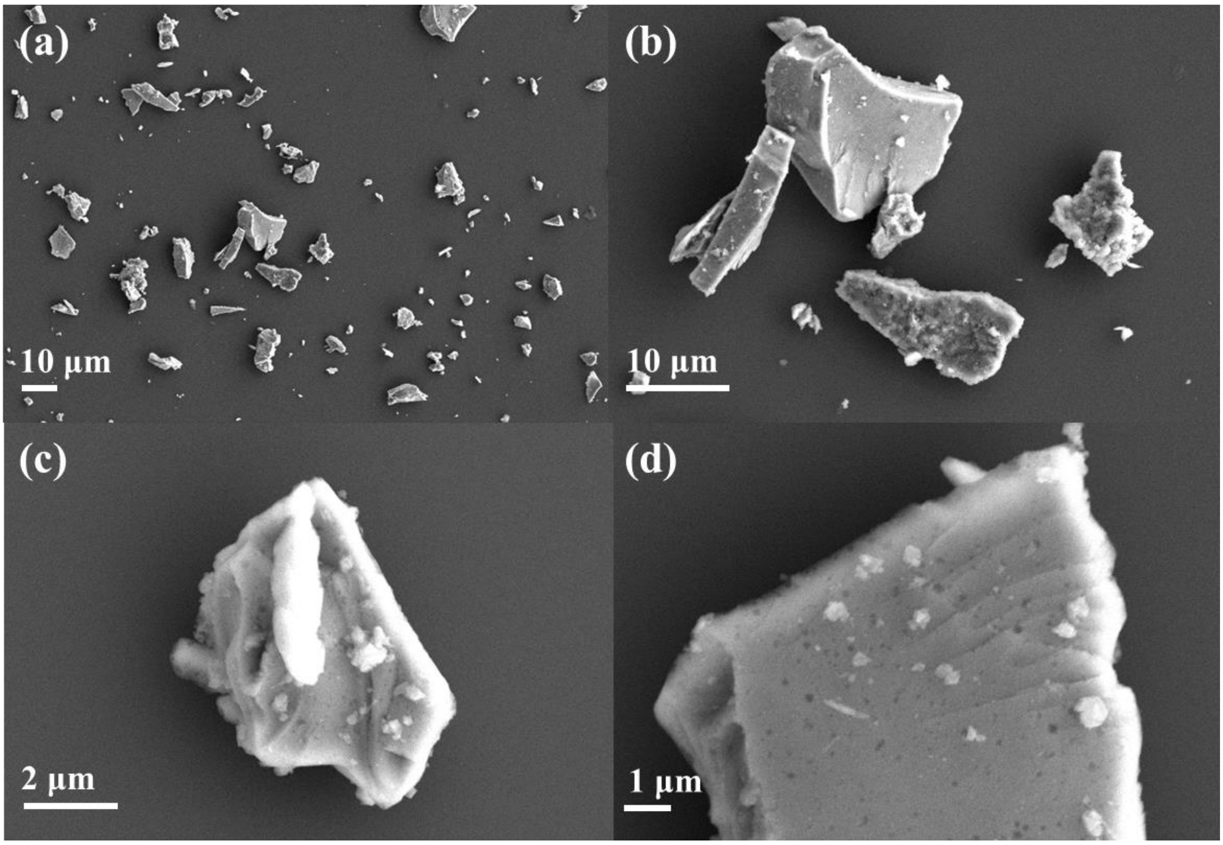

Figure S17. SEM images (a-d) of metallic Mn with different magnifications. The Mn contained large plate-shaped particles with varying sizes.

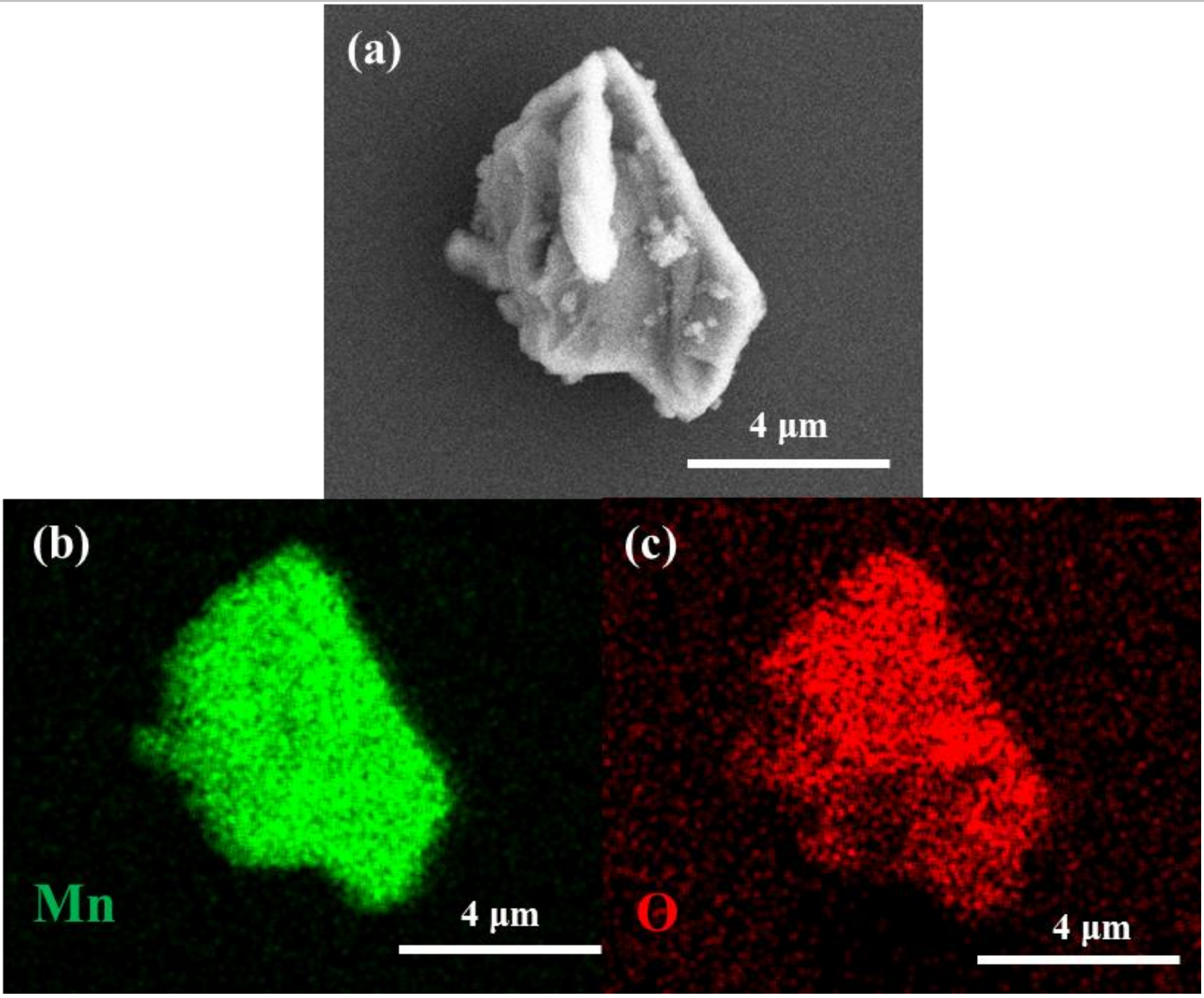

Figure S18. The SEM image (a) and the EDX mapping of Mn (b) exhibiting a homogenous distribution of manganese within the structure without much oxygen (<1) content (c). Detailed atomic % of the distribution of the elements obtained by EDX is listed in Table S1.

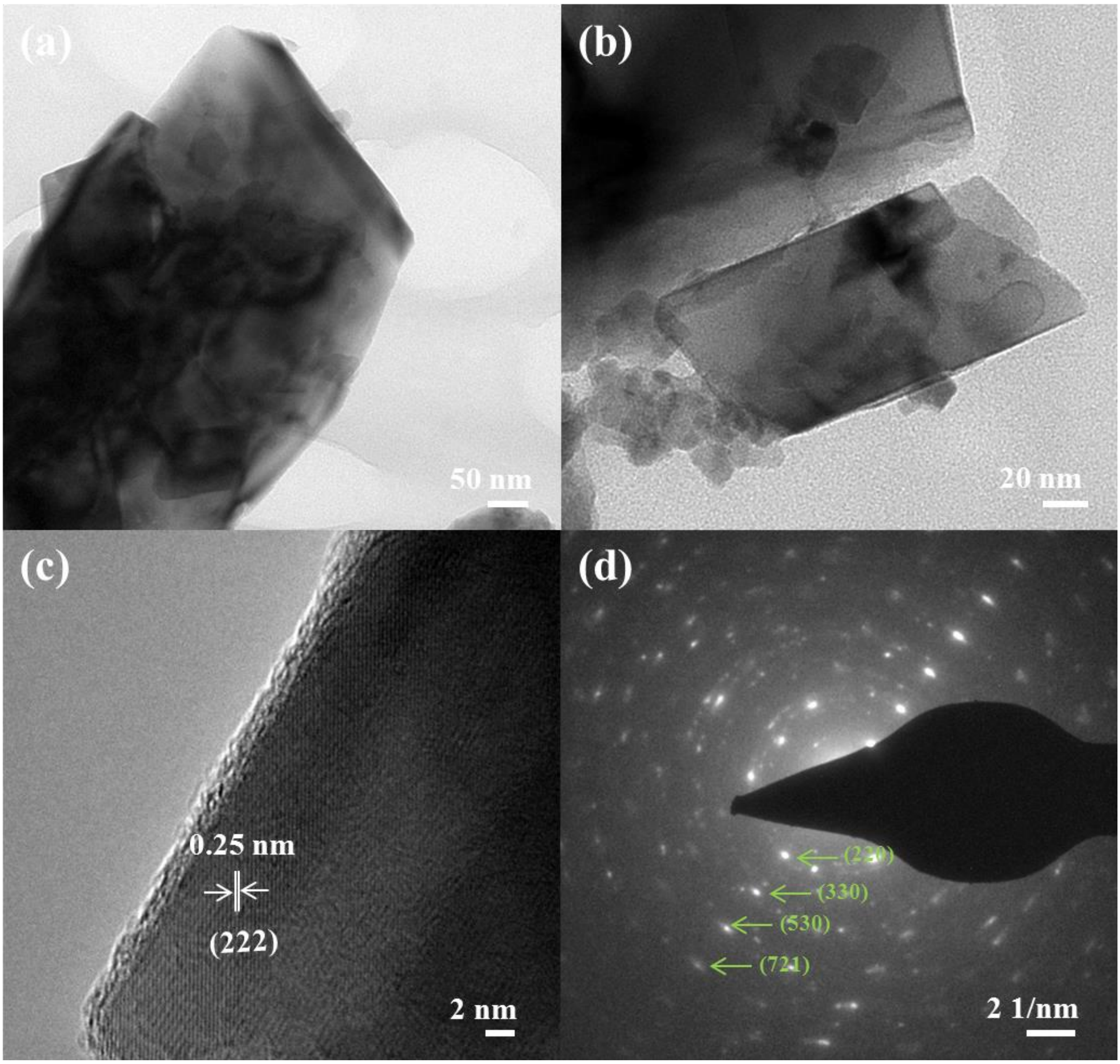

Figure S19. The TEM image (a) and High-resolution TEM (HR-TEM) images (b, c) of Mn reveal plate-shaped particles with varying sizes. The lattice fringes corresponding to the crystallographic plane (222) at d = 0.25 nm of Mn were detected from HR-TEM (c). The SAED pattern displayed diffraction rings corresponding to the crystallographic plane (220), (330), (530) and (721) at d = 0.3, 0.21, 0.15 and 0.12 nm which is also consistent with PXRD pattern confirming the phase purity of the product (JCPDS 32-637).

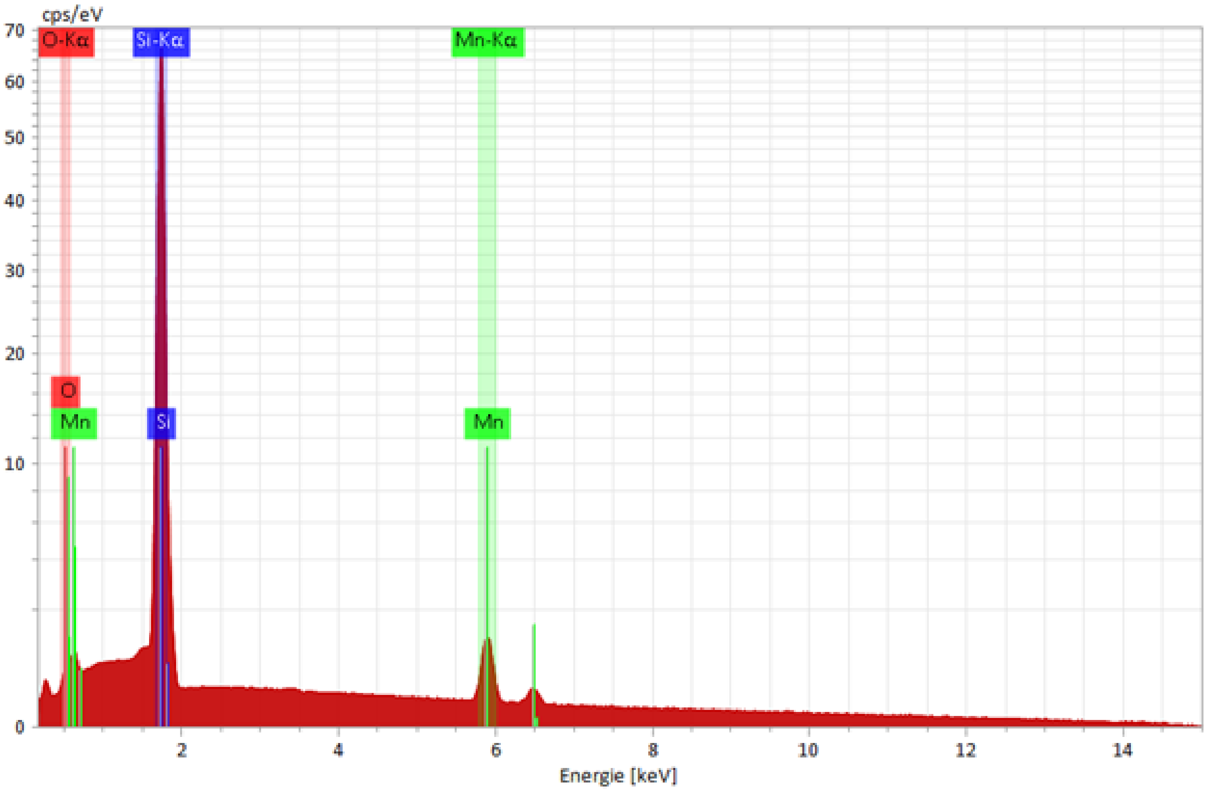

Figure S20. The EDX mapping spectrum of Mn.

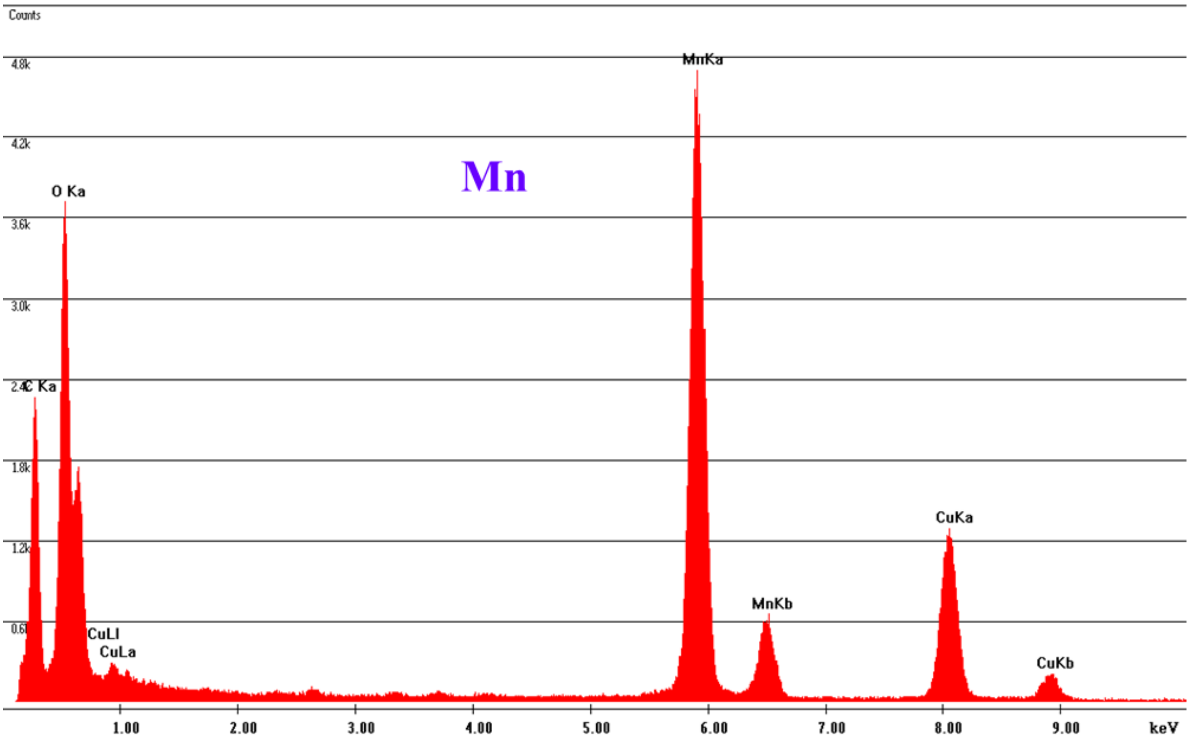

Figure S21. The EDX analysis of Mn. The peaks for copper can be unambiguously correlated to the TEM grid (carbon film 1on 300 mesh Cu-grid).

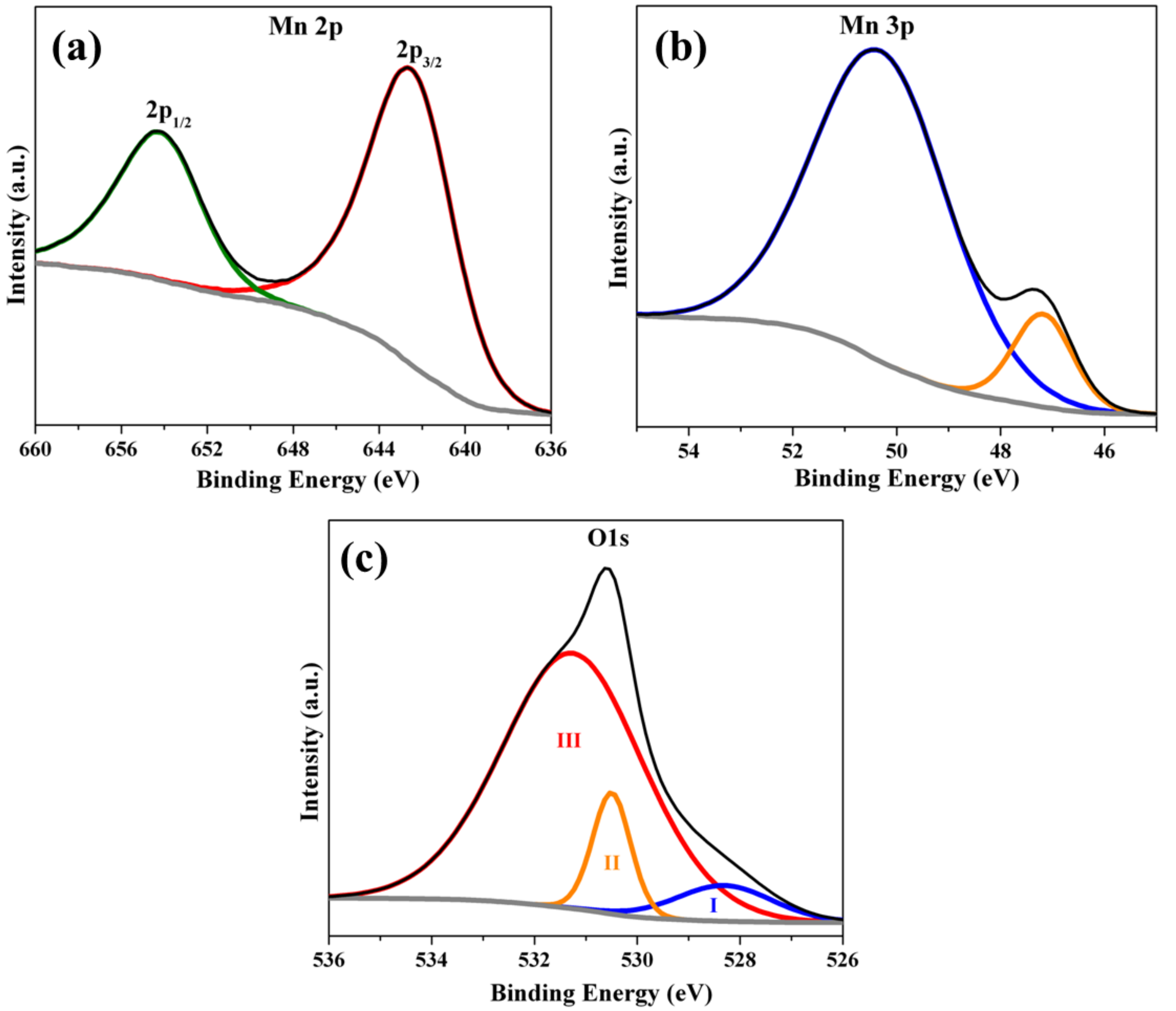

Figure S22. The high-resolution deconvoluted (a) Mn 2p, (b) Mn 3p and (c) O1s XPS spectra of Mn. The Mn $2p_{3/2}$ and Mn $2p_{1/2}$ spectra exhibited sharp peaks at the binding energy of 642.4 eV and 654.3 eV (a) that are very close to the oxidation state of Mn^{IV} (MnO_2) indicating that the Mn metal is aerially oxidized on the surface (a).[16] Similarly, the Mn 3p spectra exhibited two peaks at the binding energy of 47.3 eV and 50.2 eV corresponding to the metallic Mn and Mn^{IV} (b).[16c,17,21] The O1s XPS spectra could be deconvoluted into three peaks (I, II and III) where the small peak at ~529 is due to the formation of oxide species while the peaks at 530.8 eV and a broad peak at 531.8 eV can be ascribed to hydroxylation.[5,7d,19b-e,20]

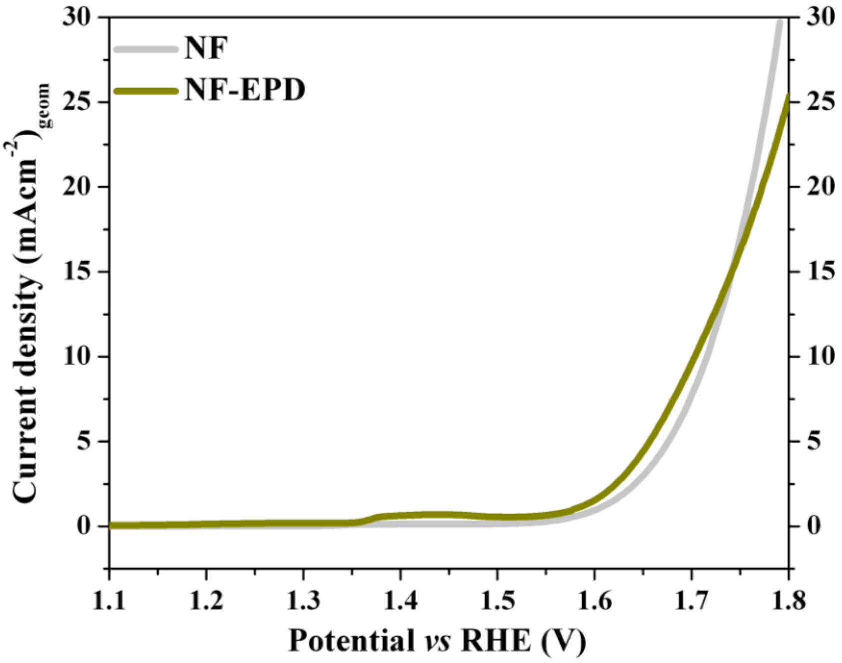

Figure S23. To know whether bare NF can be activated under EPD conditions, we applied a potential difference of 10 V in a mixture of iodine and acetone (without any catalyst) in exactly in a similar condition) to that of catalysts and subsequently recorded the LSV's (5 mVs^{-1}) in 1 M KOH solution. The LSV curves of both bare and EPD NF showed insignificant change activity under applied potential conditions.

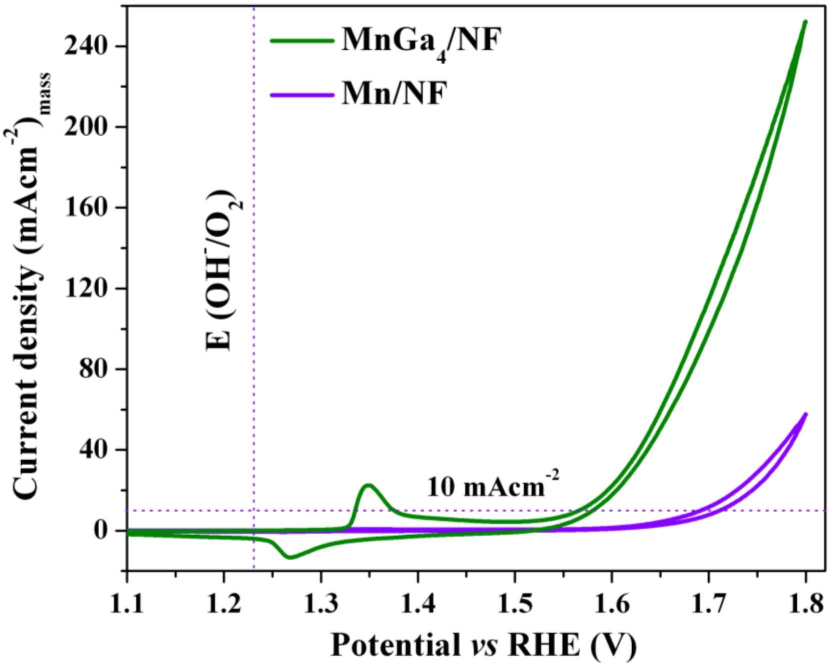

Figure S24. The mass normalized (loading 2 mg) current density plots of $MnGa_4$/NF and Mn/NF obtained from the values after OER CA in aqueous 1 M aqueous KOH for OER at a sweep rate of 5 mVs^{-1}.

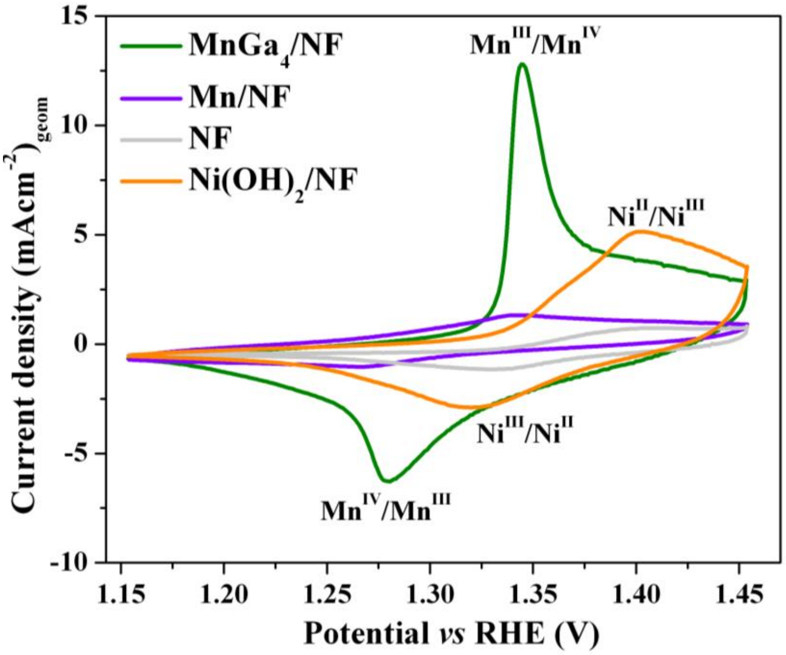

Figure S25. The CV's of $MnGa_4$ and Mn measured between 1.15 to 1.45 V (vs. RHE) in 1 M aqueous KOH solution with a sweep rate of 5 mVs^{-1} featuring a redox pair corresponding to the oxidation of low-valent manganese species to their higher valance.[5,22] The measured $Ni(OH)_2$ and NF references showed significant shift (consistent with the literature[23]) in redox peaks compared to $MnGa_4$ and Mn materials indicating the oxidation/reduction of Mn,[5,22a-c] although a slight amount of oxidation of Ni in NF cannot completely ruled out.

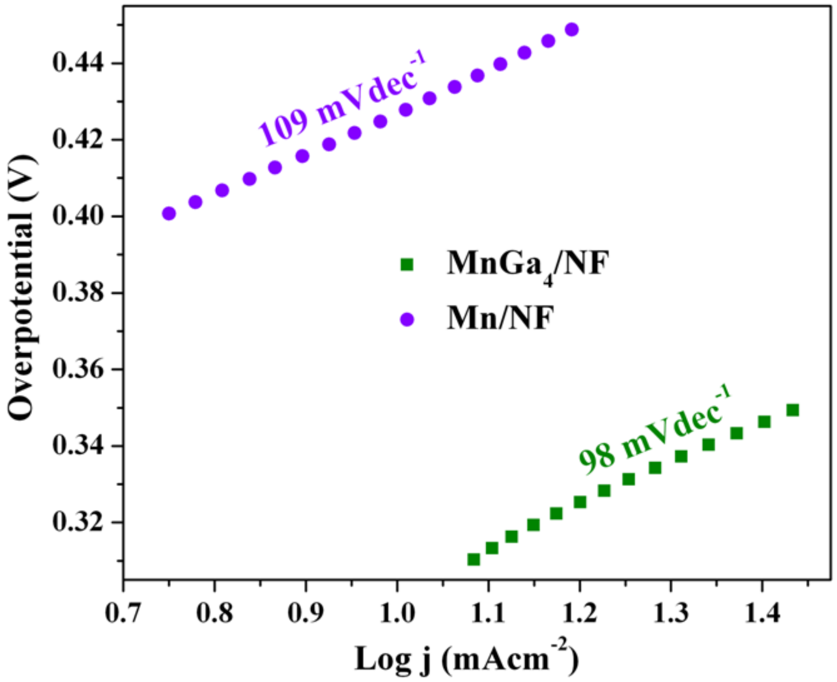

Figure S26. The Tafel plots of $MnGa_4$ and Mn measured at a sweep rate of 1 mVs^{-1}. The lower Tafel slope of $MnGa_4$ to that of Mn evidences a better OER reaction kinetics.[24]

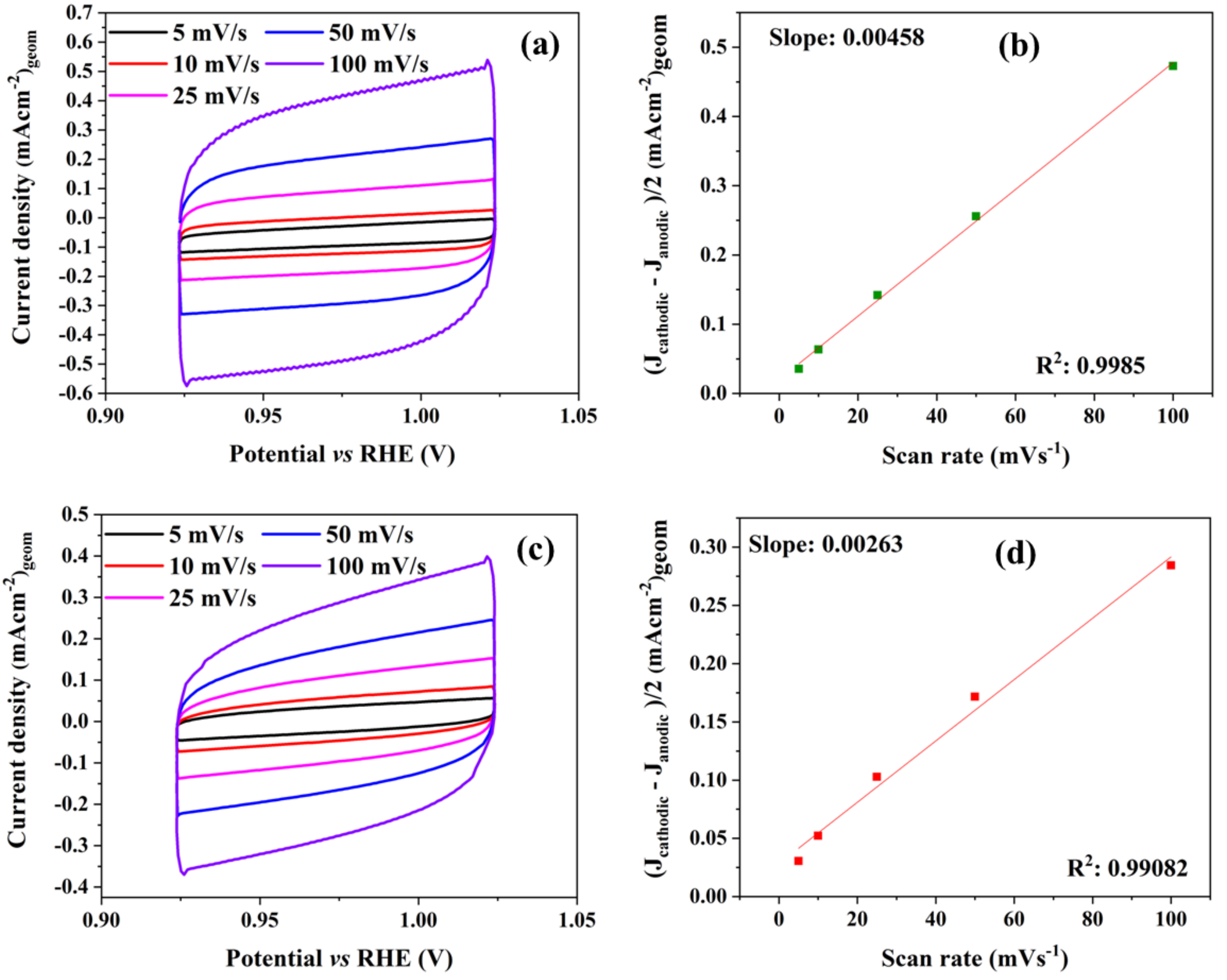

Figure S27. The ECSA analysis by the CV scans in a non-Faradaic potential range of as-prepared electrodes (a) $MnGa_4$ and (c) Mn on NF in 1 M aqueous KOH solution at a sweep rate of 5 mVs^{-1}, 10 mVs^{-1}, 25 mVs^{-1}, 50 mVs^{-1}, and 100 mVs^{-1}. Half of the differences in current density variation ($\Delta J = (J_{cathodic}-J_{anodic})/2$) at a potential of 0.975 V vs. RHE plotted against scan rate fitted to a linear regression allows the determination of double-layer capacitance (C_{dl}). The C_{dl} values obtained for (b) $MnGa_4$ and (d) Mn were 4.58 and 2.63 mF cm^{-2}. From the C_{dl} values and the specific capacitance of the material (C_s) per unit area, the ECSA was calculated.[10a,11] The attained ECSA for $MnGa_4$ was 2.7 cm^2 while Mn metal showed an ECSA of 1.54 cm^2. This demonstrates more accessible active sites in $MnGa_4$ than Mn favoring efficient adsorption and transfer of reactants to improve the electrochemical reaction.[7d,12]

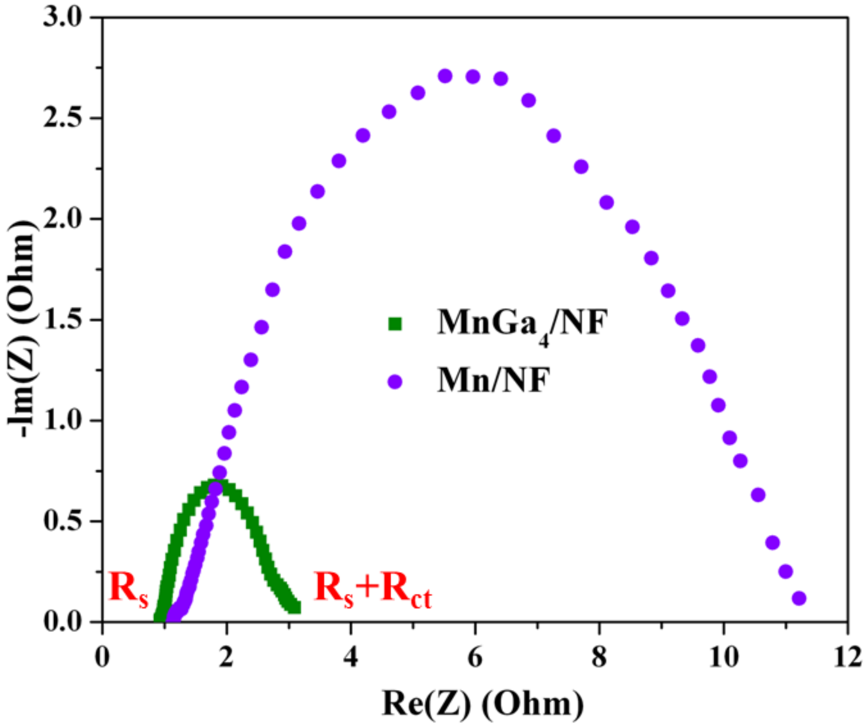

Figure S28. The Nyquist plot obtained from EIS. The R_s and R_{ct} represent solution resistance and charge transfer resistance. A highly reduced R_{ct} value was obtained for $MnGa_4$ in comparison to Mn, suggesting faster charge transfer process.[10-12]

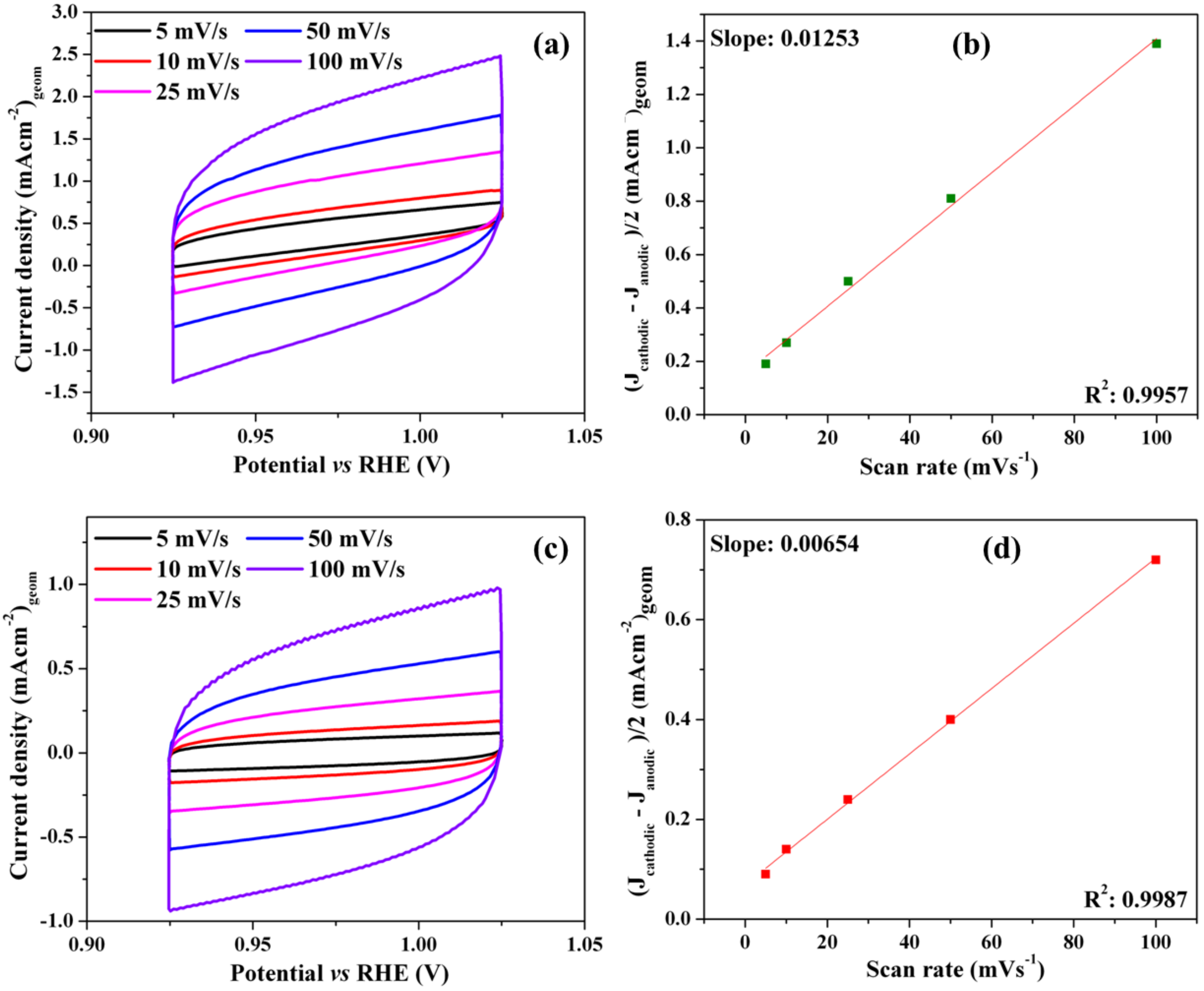

Figure S29. The ECSA analysis of (a) $MnGa_4$ and (c) Mn on NF electrodes after 24 h of OER CA. The CV scans were conducted in a non-Faradaic potential range in 1 M aqueous KOH solution at a sweep rate of 5 mVs^{-1}, 10 mVs^{-1}, 25 mVs^{-1}, 50 mVs^{-1}, and 100 mVs^{-1}. Half of the differences in current density variation ($\Delta J = (J_{cathodic}-J_{anodic})/2$) at a potential of 0.975 V vs. RHE plotted against scan rate fitted to a linear regression allows the determination of double-layer capacitance (C_{dl}). Almost thrice higher C_{dl} values, 12.53 and 6.54 mF cm^{-2} were obtained for (b) $MnGa_4$ and (d) Mn after OER CA demonstrating the substantial transformation of the as-prepared material. The attained ECSA for $MnGa_4$ was 7.4 cm^2 while Mn metal showed an ECSA of 3.84 cm^2.

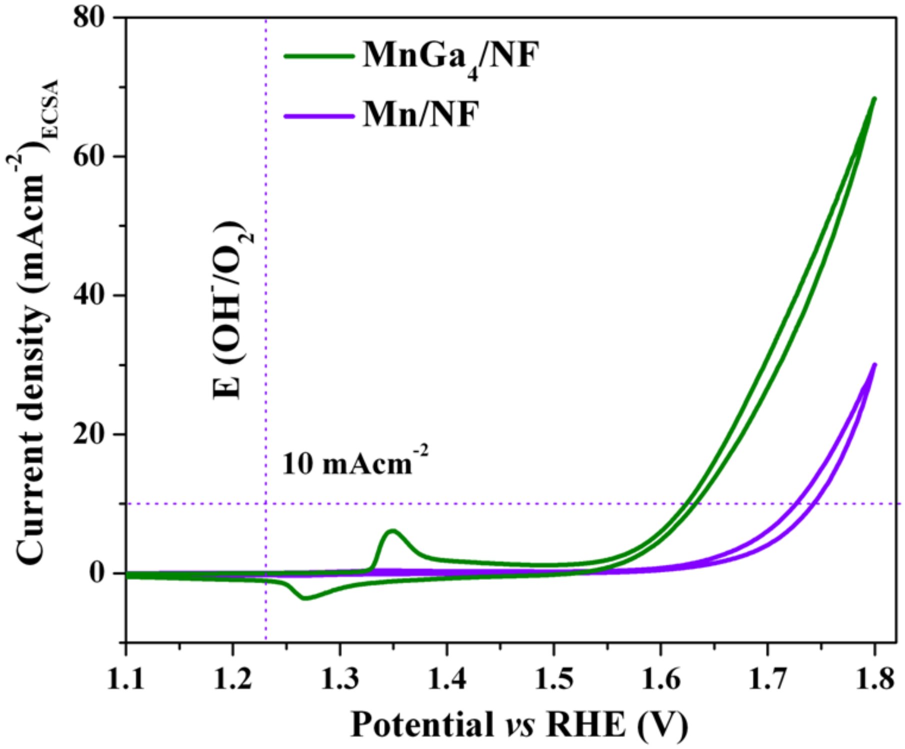

Figure S30. The ECSA normalized current density plots of $MnGa_4$/NF and Mn/NF obtained from the values after OER CA in aqueous 1 M aqueous KOH for OER at a sweep rate of 5 mVs^{-1}.

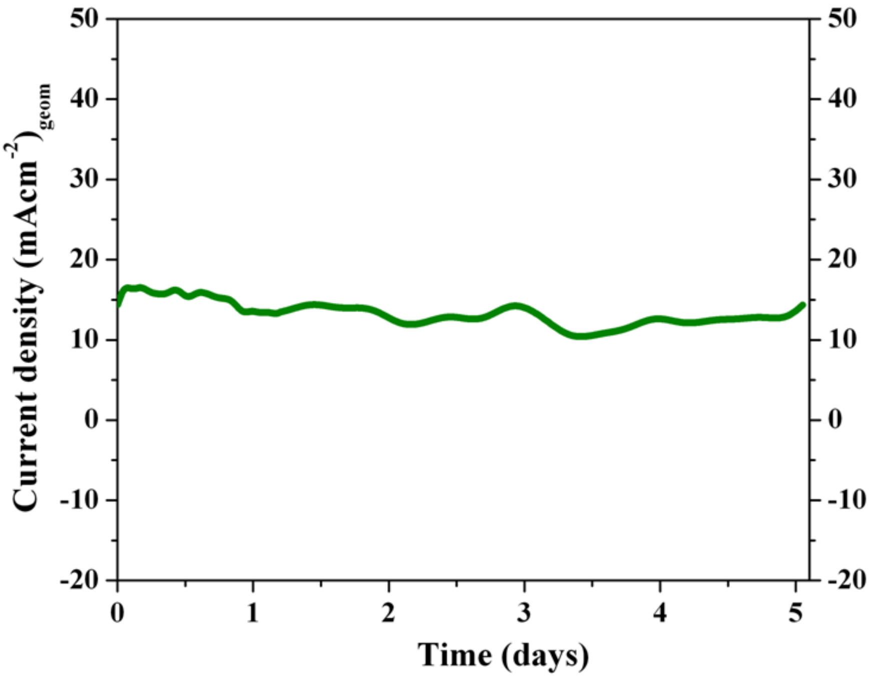

Figure S31. The CA response of $MnGa_4$/NF measured in OER conditions at an overpotential of 300 mV in 1 M aqueous KOH solution. The catalyst was stable for more than five days. The minor fluctuation was in current is attributed to the evaporation of the electrolyte over time, and by adjusting the level of the electrode, a steady current was reached again.[25]

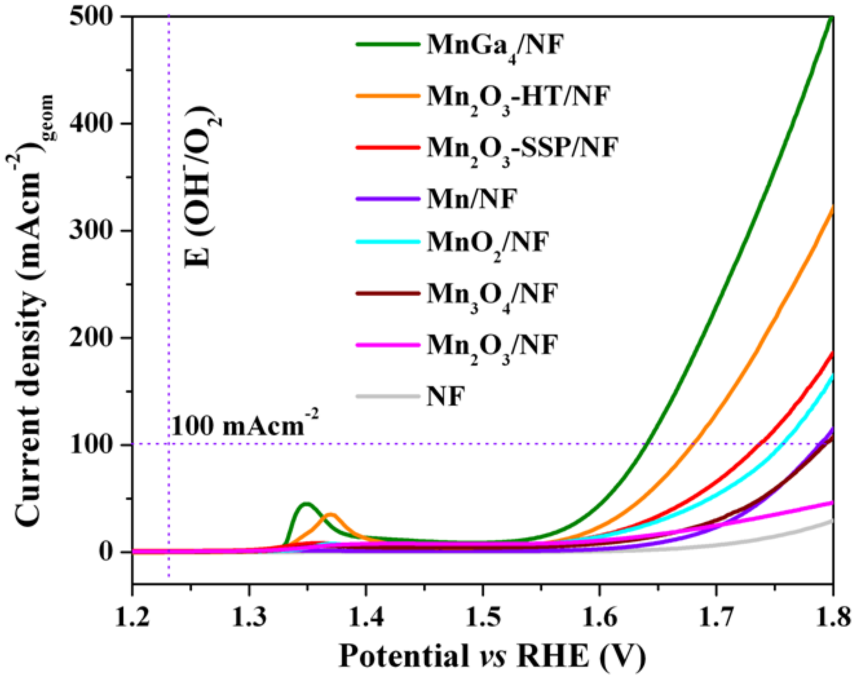

Figure S32. The LSV of $MnGa_4$ and Mn and their comparison to that of different synthetic and commercial manganese oxides in aqueous 1 M KOH on NF (mass loading 2 $mgcm^{-2}$) at a scan rate of 5 mVs^{-1}. The bar diagram of overpotentials at 10 $mAcm^{-2}$ is shown in Figure 2 (main text).

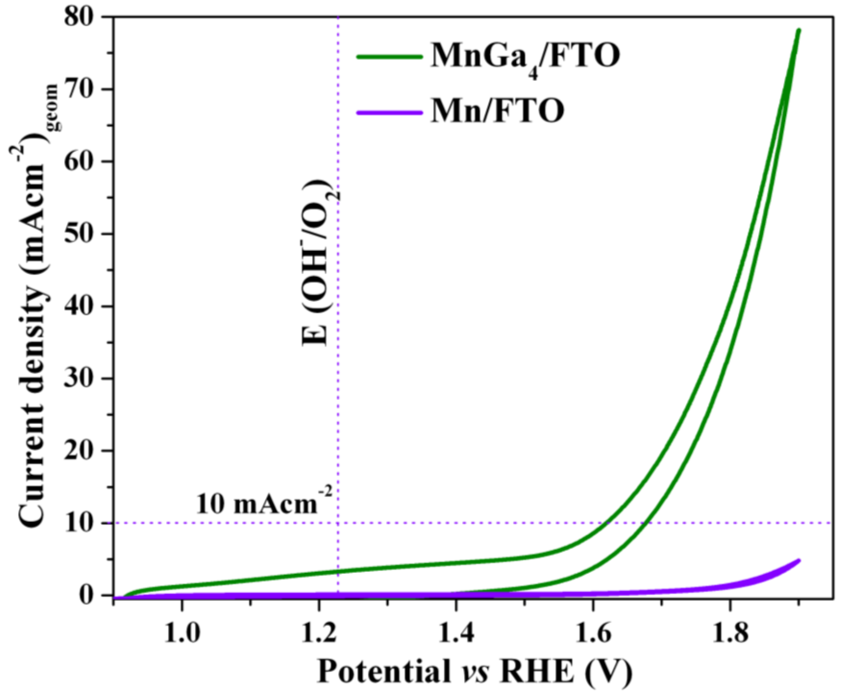

Figure S33. The CV's of $MnGa_4$ and Mn measured on FTO (mass loading 0.7 $mgcm^{-2}$) as an electrode substrate in aqueous 1 M KOH at a scan rate of 10 mVs^{-1}. A similar trend in OER activity in comparison to NF was observed.

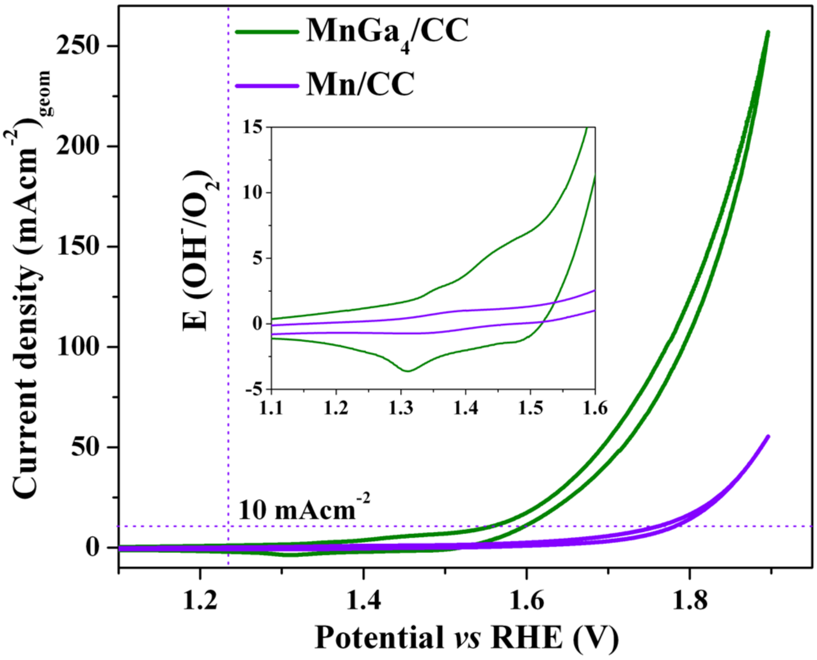

Figure S34. The CV's of $MnGa_4$ and Mn measured on carbon cloth (CC) as an electrode (mass loading 1.1 $mgcm^{-2}$) substrate in aqueous 1 M KOH at a scan rate of 10 mVs^{-1}. A similar trend in OER activity in comparison to NF and FTO was observed. The inset shows the redox peaks of Mn from lower to higher oxidation state during OER.

Calculation of Faradaic efficiency

The Faradaic efficiency (FE) of $MnGa_4$ in 1M KOH towards oxygen evolution reaction was measured with $MnGa_4$ on nickel foam in a closed electrochemical cell. The cell and the electrolyte were first degassed with Argon for 30 min under stirring. Afterward, the constant current density of 10 $mAcm^{-2}$ was applied for a certain period. At the end of electrolysis, the gaseous samples were taken out of the headspace by a gas-tight syringe and analyzed by a GC calibrated for O_2. Every injection step was repeated at least three times, and the average value is presented.

The Faradaic efficiency (FE) is calculated based on:

$$FE(O_2,\%) = \frac{V_{O_2} \times 4 \times F}{V_m \times j \times t} \times 100\%$$

V_{O2} is the evolved volume of oxygen, F is the Faraday constant (96485.33289 C/mol), V_m is the molar volume of the gas, j is the current density (10 $mAcm^{-2}$), and t is the period of electrolysis.

Table S4: Calculation of Faradaic efficiency

	j ($mAcm^{-2}$)	t (sec)	V_{O2} (mL)	FE (O_2,%)
$MnGa_4$	10	300	0.169	97

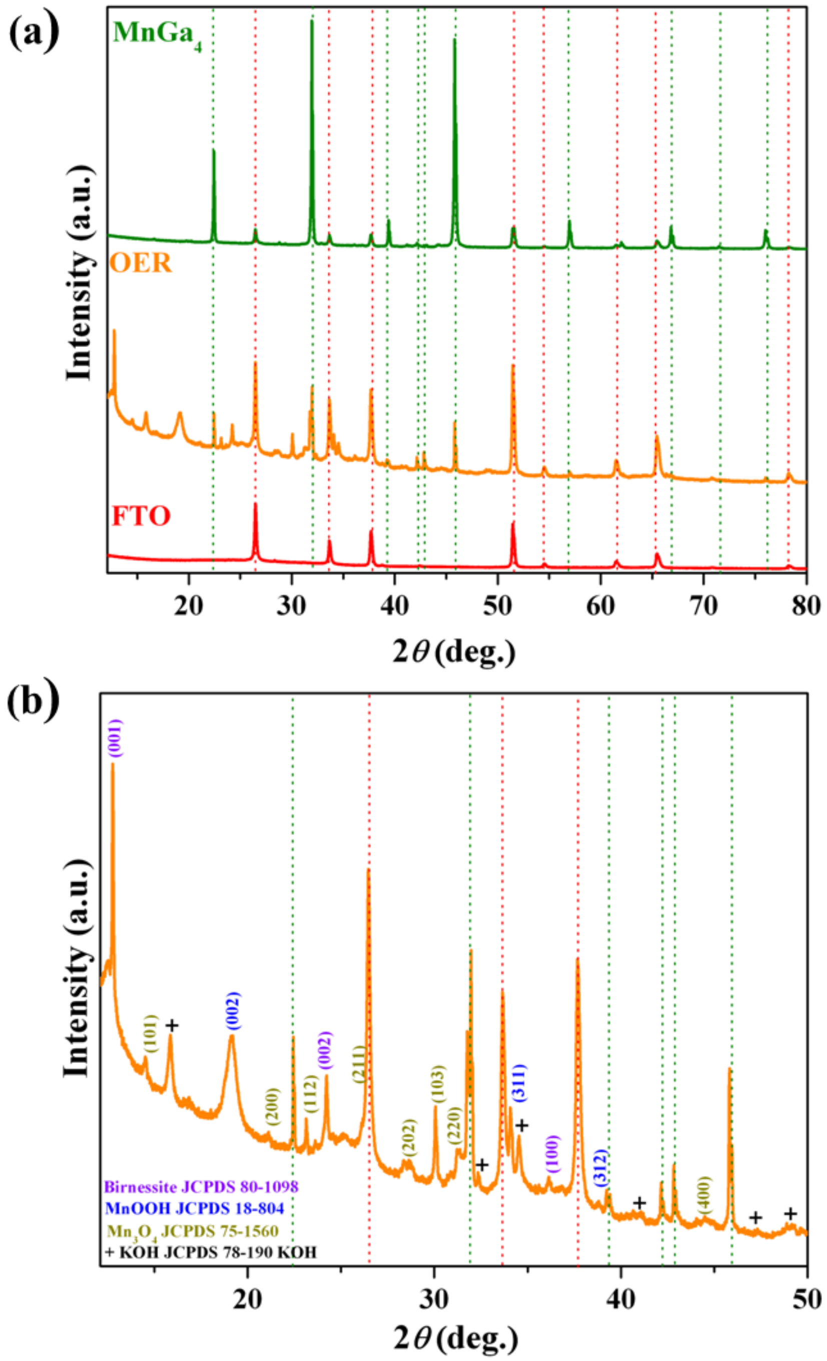

Figure S35. (a) The PXRD pattern of as-synthesized intermetallic $MnGa_4$, after OER CA (24 h) and bare FTO. The respective reflections of as-synthesized $MnGa_4$ and bare FTO are marked in broken green and red lines. The PXRD pattern of $MnGa_4$ after OER CA experiments was indexed carefully (b) that showed the presence of (K-) birnessite δ-MnO_2 (JCPDS 80-1098), feitknechtite β-MnOOH (JCPDS 18-804) and hausmannite α-Mn_3O_4 (JCPDS 75-1560) phases. Beside manganese oxides, some amount of KOH from the electrolyte was also attained.

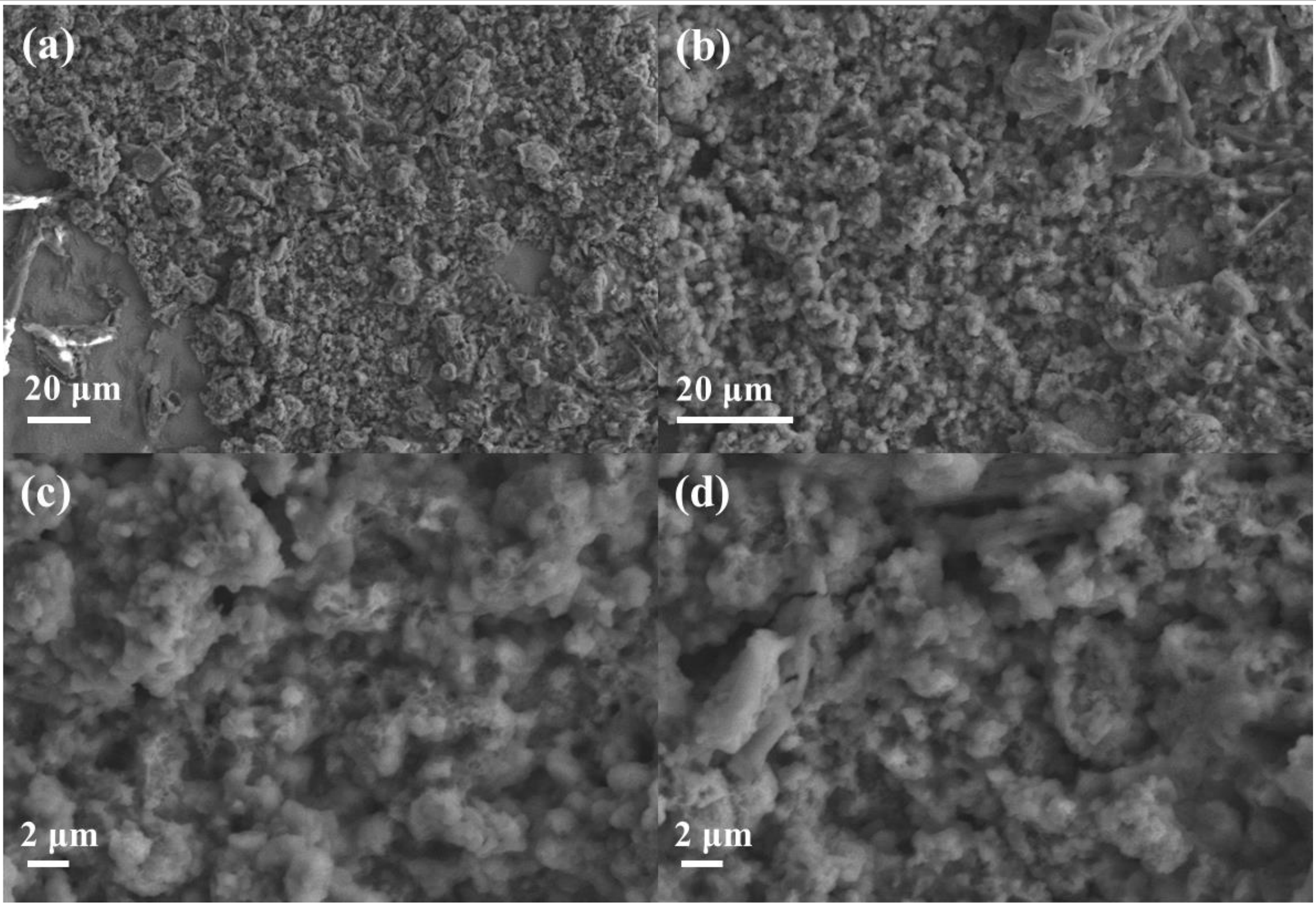

Figure S36. SEM images (a-d) of $MnGa_4$ films after OER CA (24 h) showing different magnifications. The particles displayed heavy corrosion forming porous structure (c, d) and suggested the transformation of the initial $MnGa_4$ into an entirely new phase.

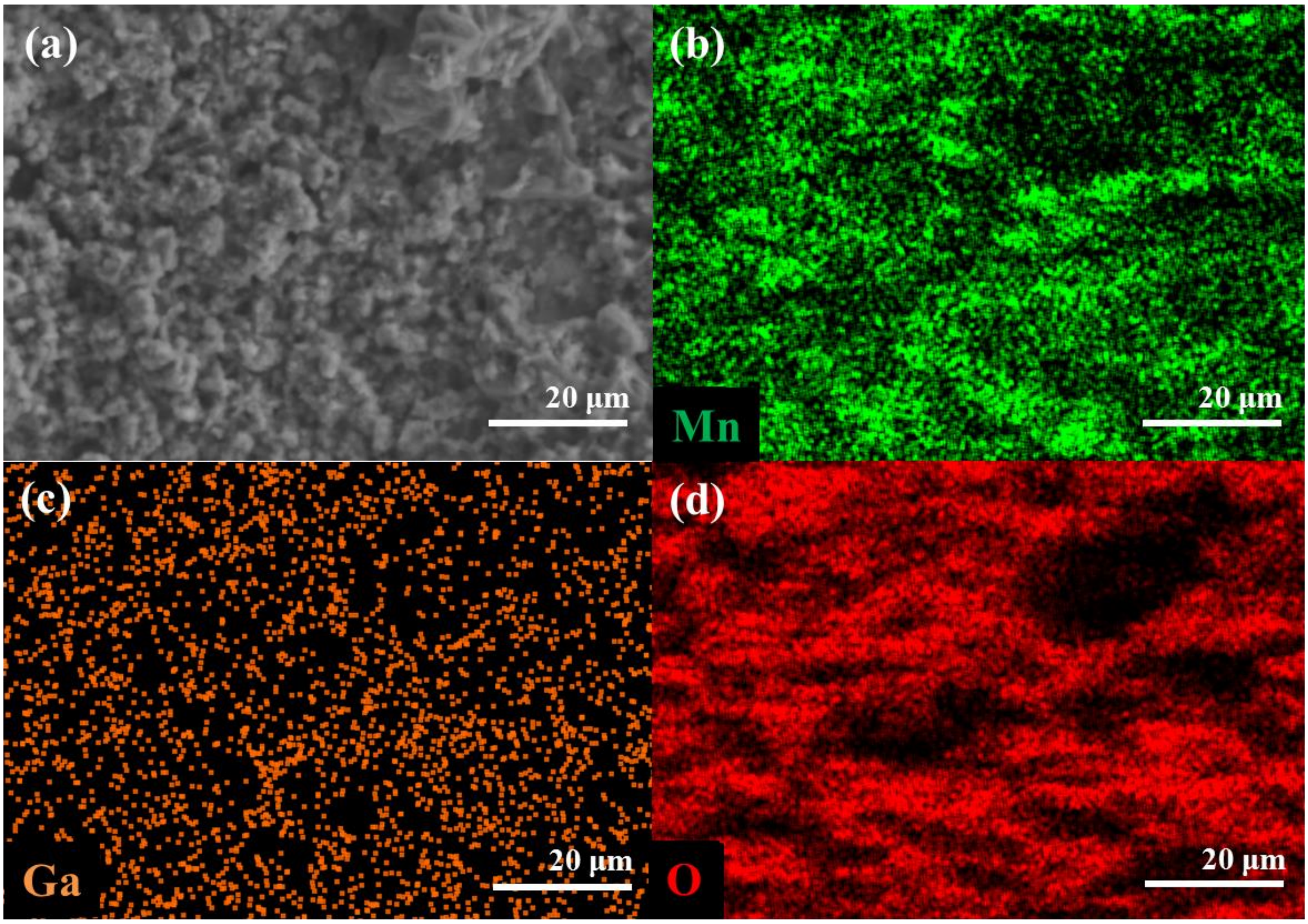

Figure S37. The SEM image (a) and the respective EDX mapping of $MnGa_4$ after OER CA (b-d). The spectra exhibit a significant change in the morphology as well as in the structure. Although the manganese (b) and oxygen (d) were homogeneously distributed within the particles, the gallium (c) atoms mostly disappeared from the structure indicating heavy corrosion of the particles under OER conditions. Detailed atomic % of the distribution of the elements obtained by EDX is listed in Table S1.

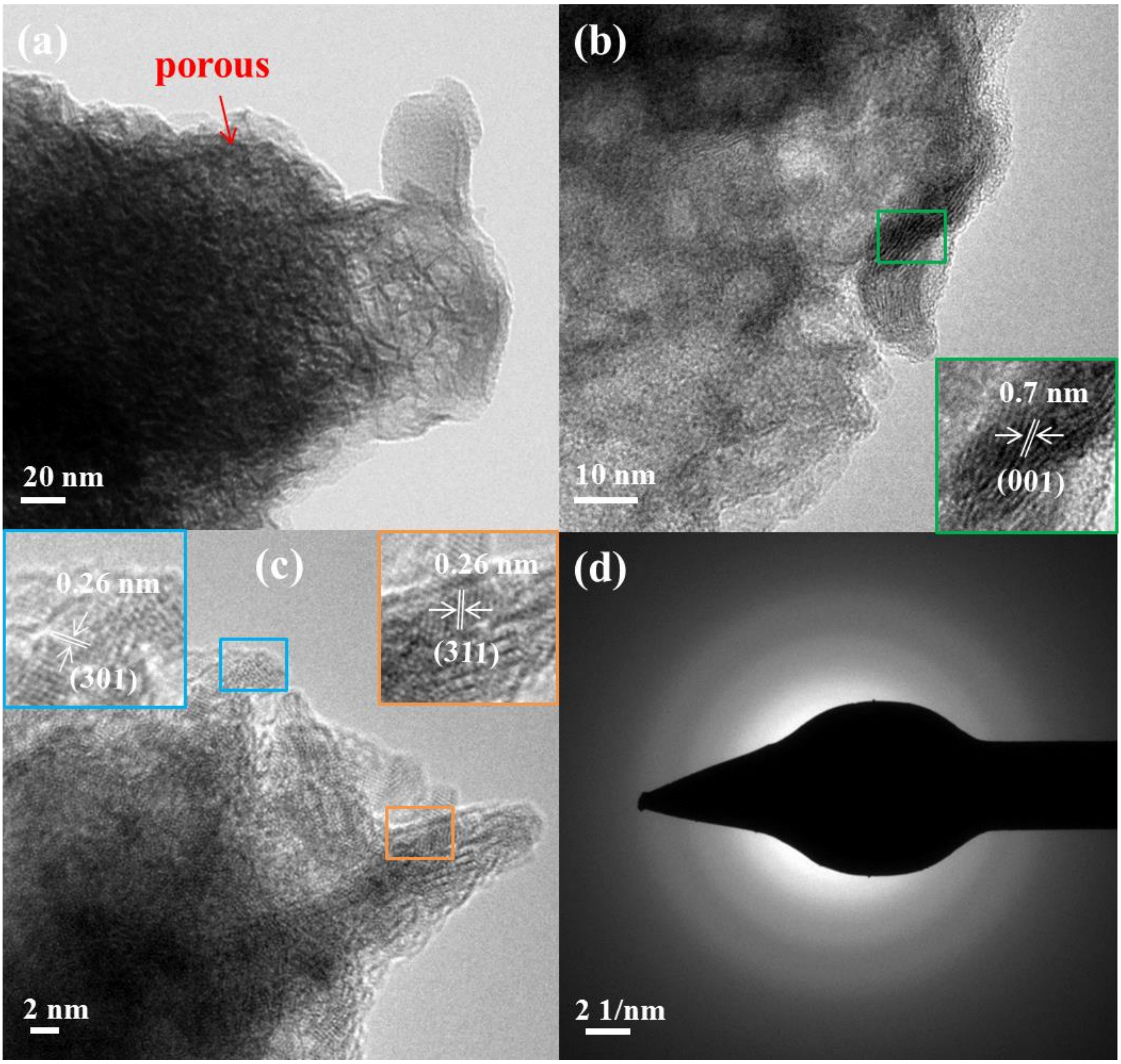

Figure S38. (a) TEM (a) HR-TEM images (b, c) and SAED (d) pattern of $MnGa_4$ after OER CA (24 h). The TEM images (a) showed transformation of $MnGa_4$ completely into a hollow porous nanostructure. A closer look at the edge (b) of nanostructure suggested a lattice spacing of 0.7 nm that can be assigned to the (001) plane of (K-) birnessite δ-MnO_2 structure (JCPDS 80-1098). Furthermore, the distance of ~0.26 nm was also attained that could be ascribed to either (301) planes of feitknechtite β-MnOOH (JCPDS 18-804) or (311) planes of hausmannite α-Mn_3O_4 (JCPDS 75-1560) structure. The obtained TEM results are consistent with the PXRD pattern.

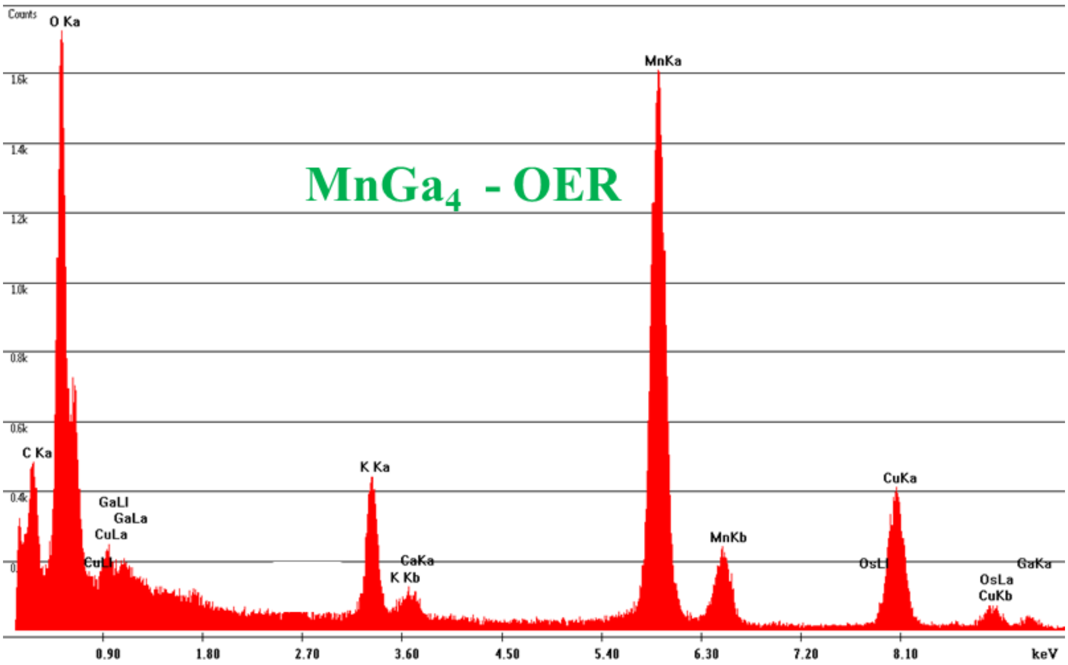

Figure S39. The EDX analysis of $MnGa_4$ after OER CA (24 h). The weakening of Ga peaks and enhancement in Mn peaks were observed that are indicative of severe loss of Ga in OER conditions. The presence of K is due to the electrolyte (in the form of KOH, and the peaks for copper can be unambiguously correlated to the TEM grid (carbon film on 300 mesh Cu-grid).

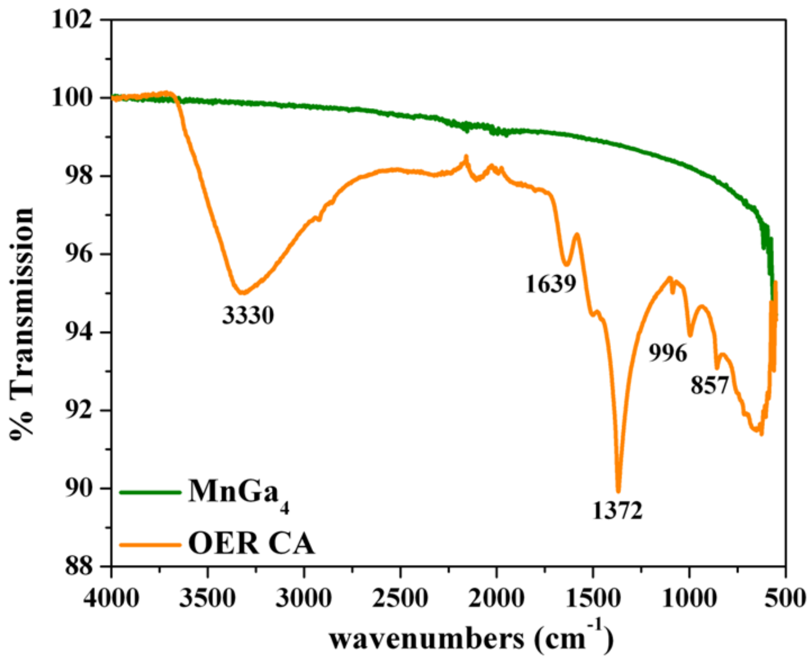

Figure S40. The FTIR-spectra of $MnGa_4$ and the films after OER CA. After the OER, a broad band was observed at ~3330 cm^{-1} which is due to stretching vibrations of interlayer water molecules whereas the band at ~1640 cm^{-1} could be assigned to the bending vibration of H_2O and structural OH groups.[26] The bands between 800 and 1400 cm^{-1} (1372, 996 and 857 cm^{-1}) are typically assigned to the bending vibrations of –OH groups bound with Mn atoms.[27] Bands around ~700 cm^{-1} and lower are characteristic bands of manganese oxides.[26b,28]

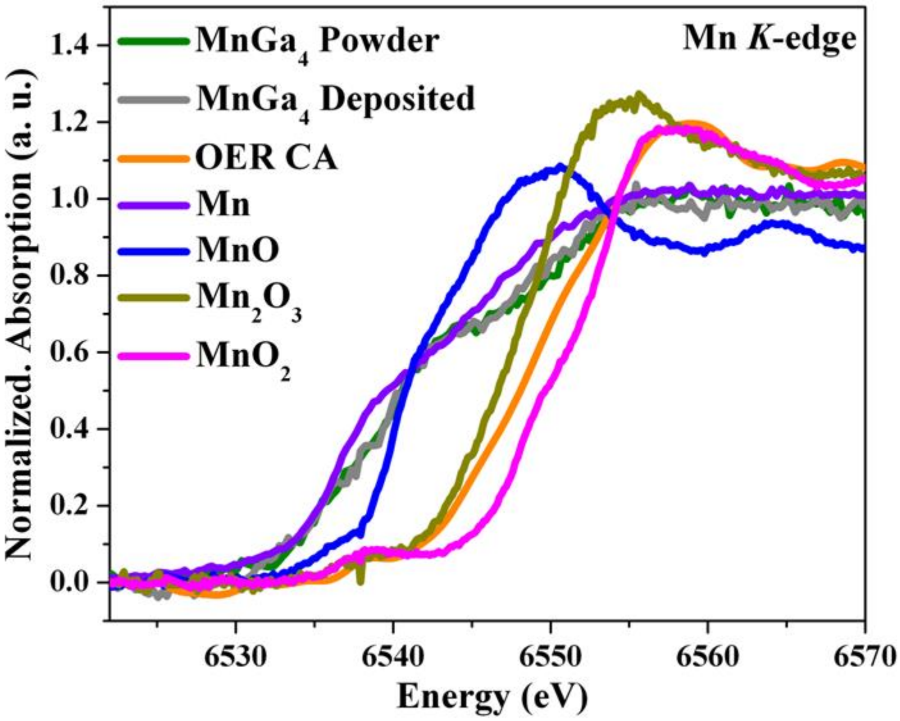

Figure S41. The Mn *K*-edge XANES spectra of as-prepared $MnGa_4$, electrophoretically deposited $MnGa_4$ film and $MnGa_4$ film after OER CA measured in the air under ambient pressure. To deduce a clear comparison, the spectra of commercial powder Mn, MnO, Mn_2O_3, and MnO_2 with different oxidation states measured in similar conditions have also been included as references. From the spectra, it was clear that the $MnGa_4$ after OER film was completely oxidized in comparison to the as-prepared and as-deposited $MnGa_4$. The oxidation state of $MnGa_4$.was found to be in between commercial Mn_2O_3 and MnO_2, indicating the transformation of original structure very close the commercial MnO_2 materials.[2,14]

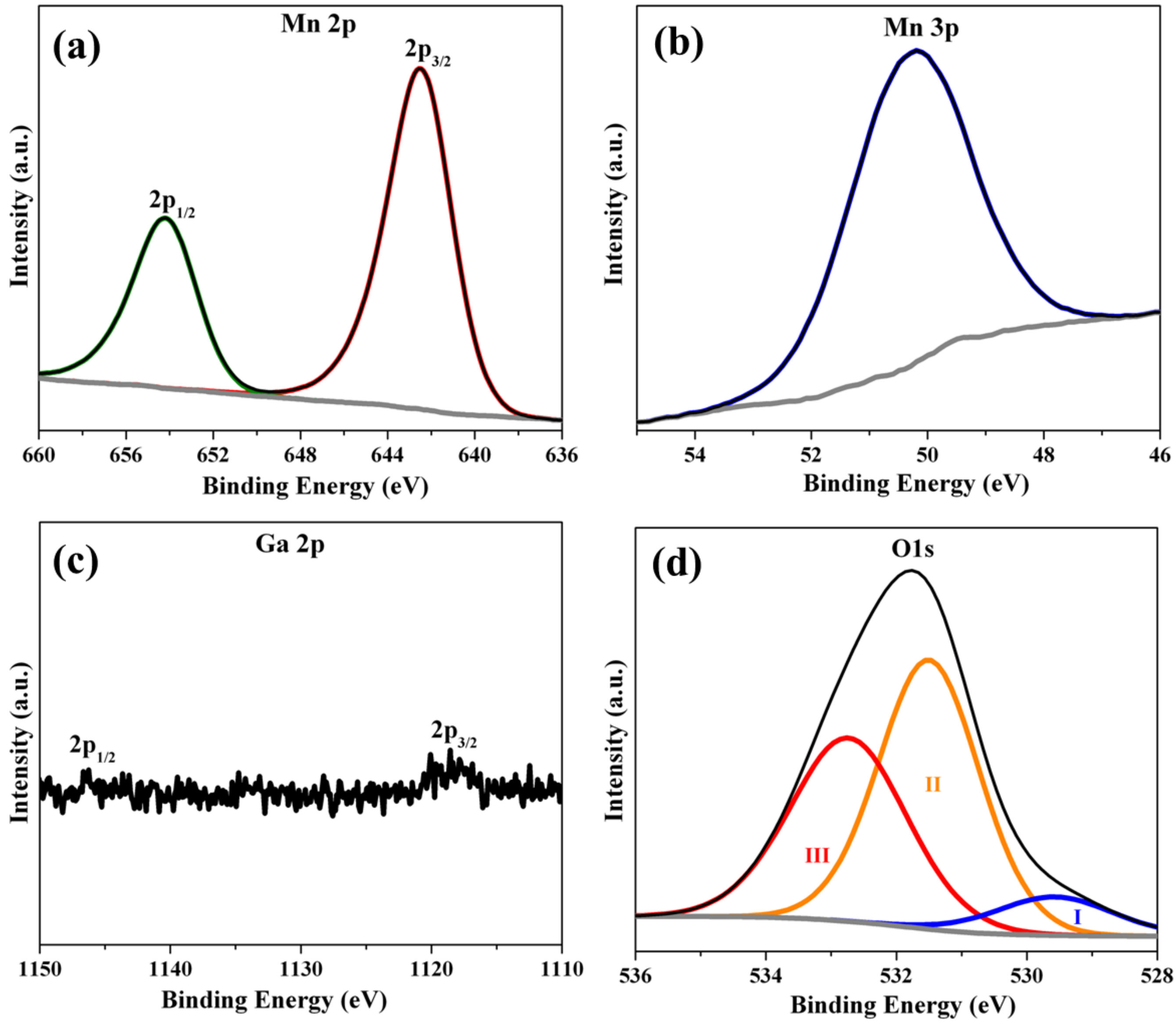

Figure S42. The high-resolution deconvoluted (a) Mn 2p, (b) Mn 3p, (c) Ga 2p and (d) O1s XPS spectra of $MnGa_4$ after OER CA (24 h). The Mn $2p_{3/2}$ and Mn $2p_{1/2}$ spectra did not show much deviation from the as-prepared samples by displaying two sharp peaks at the binding energy of 642.3 eV and 654.2 eV (a) that are very close to the oxidation state of Mn^{IV} (MnO_2).**[16]** Similarly, as shown for as prepared $MnGa_4$, the Mn 3p spectra showed a broad peak at a binding energy value of 50.2 eV that could also be assigned to Mn^{IV} (b).**[16c,17]** In the case of Ga 2p, the peaks responsible for Ga were absent, which demonstrates the massive loss of Ga from the surface of $MnGa_4$ under in situ OER conditions with the transformation of the initial structure. O1s XPS spectra further confirmed this. The O 1s spectrum was deconvoluted into three (I, II and III) peaks, the first at ~ 529 is due to the formation of oxide phase whereas the peak at the binding energy of 531.5 eV and 532.8 eV can directly be ascribed to hydroxylated (-OH/-OOH) and adsorbed water onto the surface.[5,7d,19b-e,20]

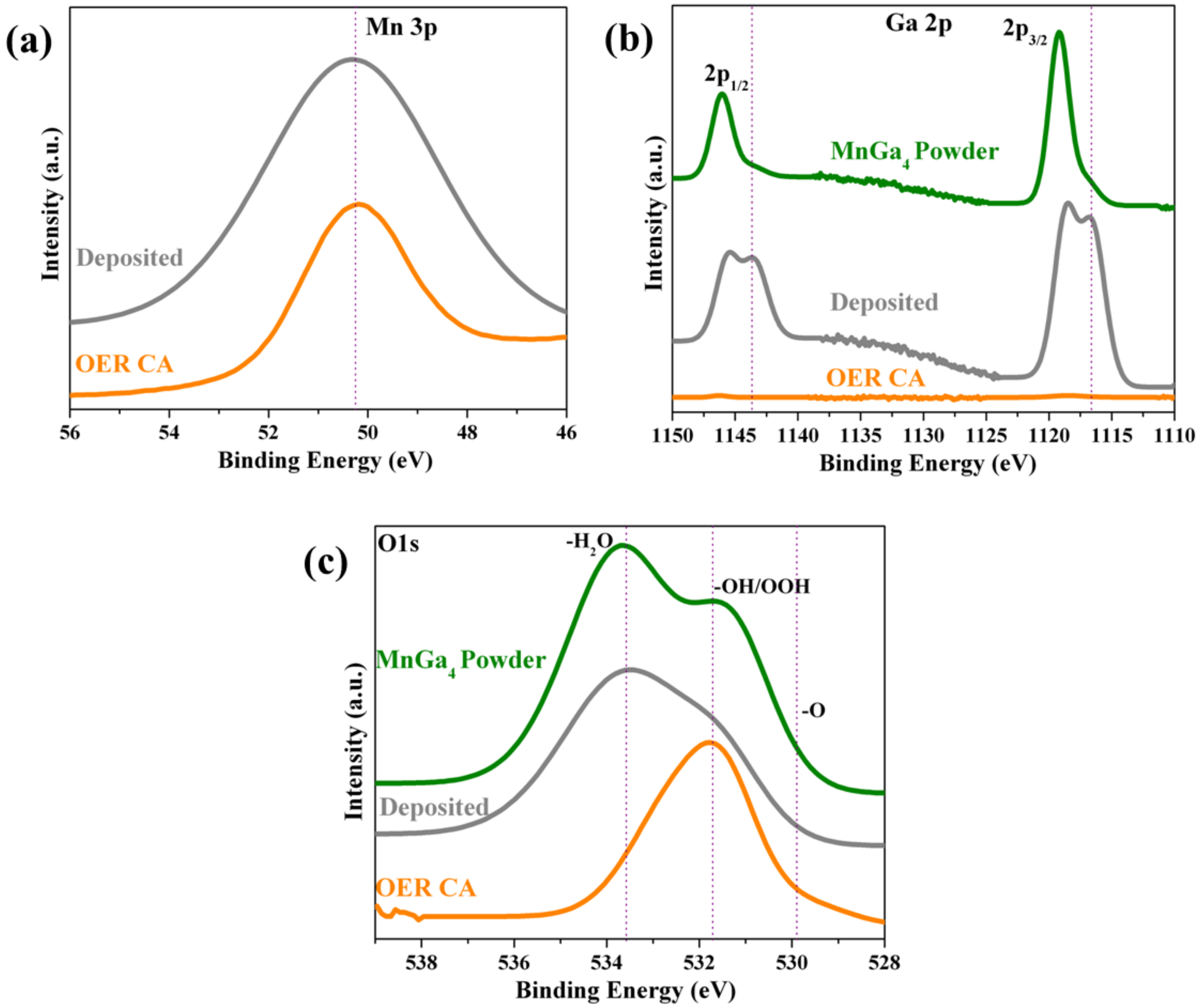

Figure S43. A direct comparison of (a) Mn 2p, (b) Ga 2p and (c) O1s XPS spectra of as-prepared, as-deposited and after OER CA films of $MnGa_4$ (24 h) indicating the in-situ transformation of $MnGa_4$ under OER conditions.

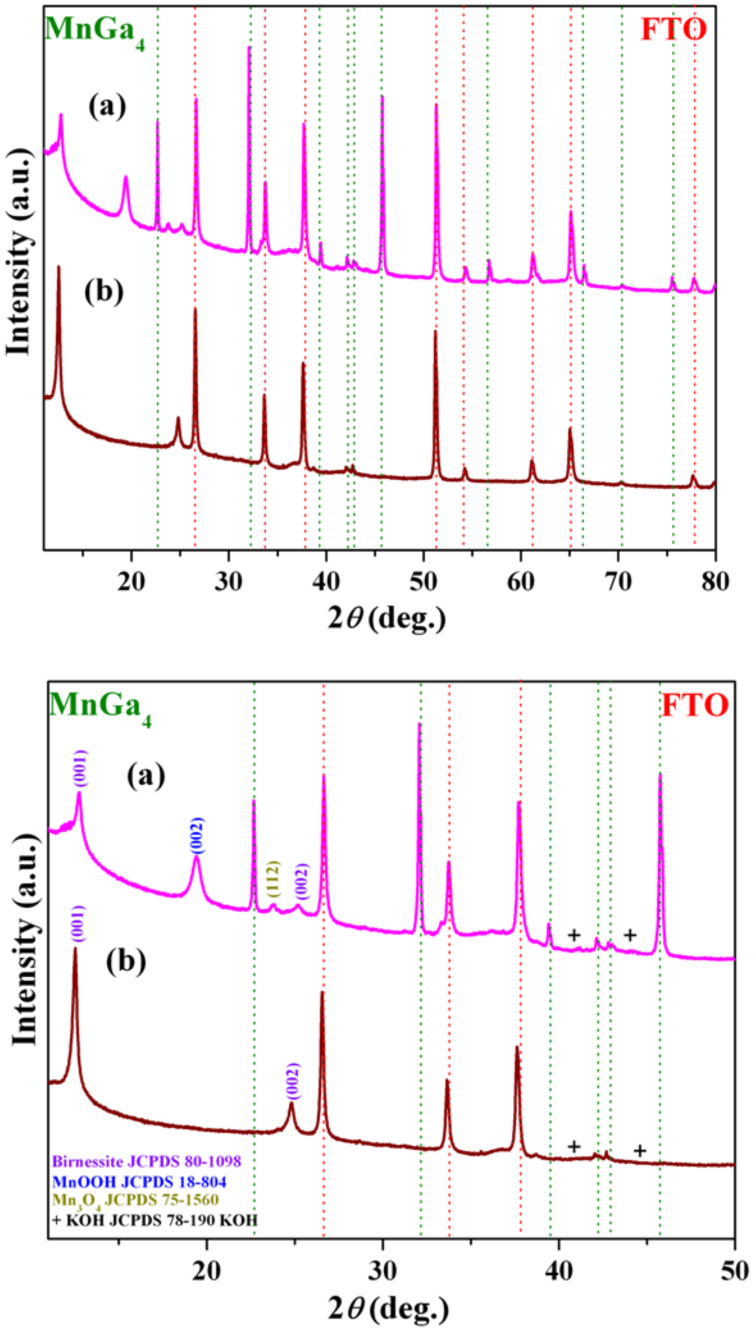

Figure S44. (a) The PXRD pattern of $MnGa_4$ films after OER CA (24 h) maintaining a current of 5 $mAcm^{-2}$ (a) and 20 $mAcm^{-2}$ (b). At lower currents (or potentials), slow transformation of $MnGa_4$ was observed forming (K-) birnessite δ-MnO_2 (JCPDS 80-1098), feitknechtite β-MnOOH (JCPDS 18-804) as major phase and hausmannite α-Mn_3O_4 (JCPDS 75-1560) minor phase whereas at potentials of 10 $mAcm^{-2}$ (Figure S35) the transformation was somewhat faster. At higher currents, a complete conversion of $MnGa_4$ into δ-MnO_2 was attained. This is a strong indication that the conversion of $MnGa_4$ takes via the α-Mn_3O_4 through β-MnOOH and finally forming most stable and active δ-MnO_2, and the conversion rate is dependent on the applied potentials.

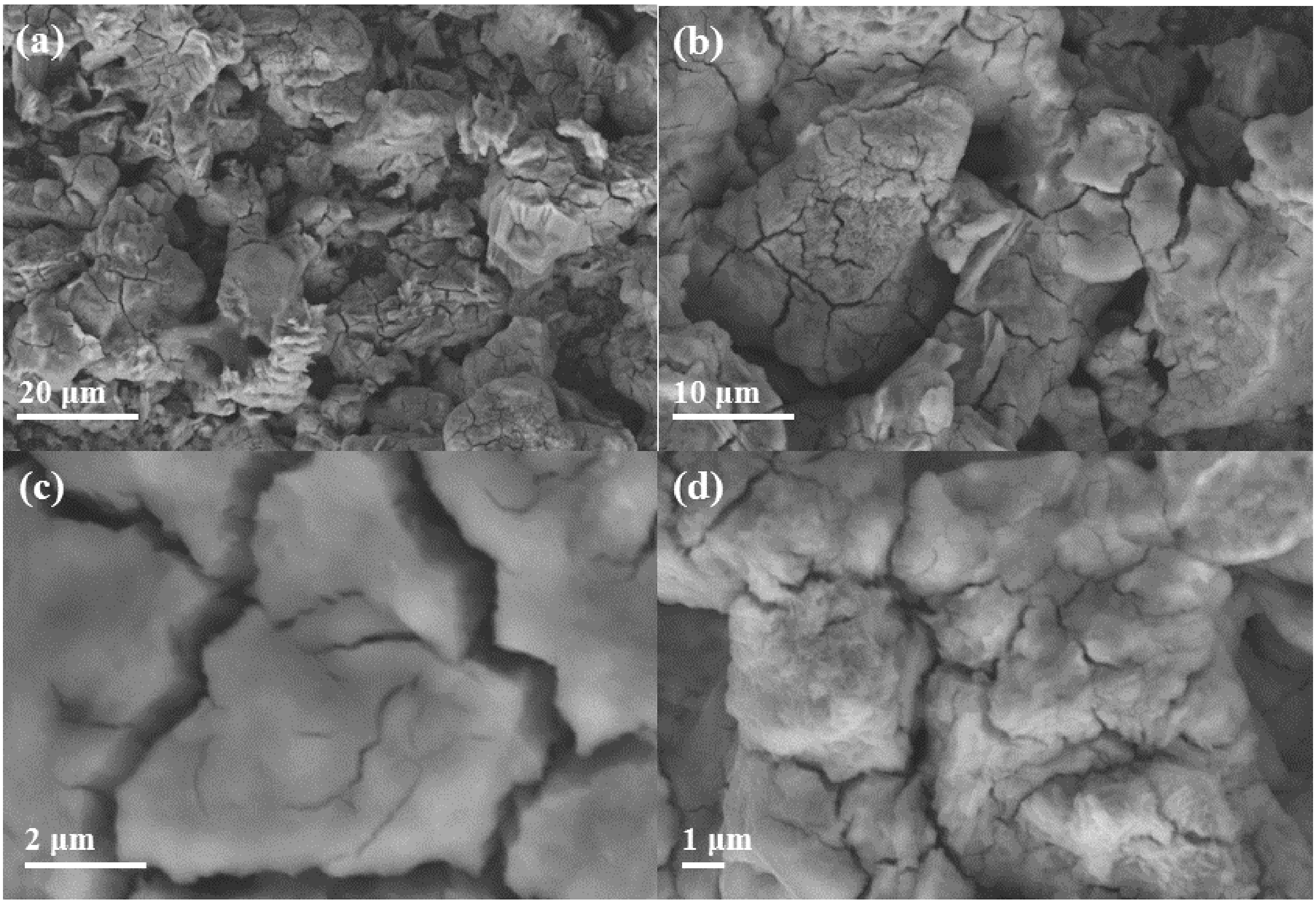

Figure S45. SEM images (a-d) of $MnGa_4$ films after OER CV (10 cycles) showing different magnifications. The particles displayed slight corrosion (slow transformation) leading to the formation of porous structure (c, d).

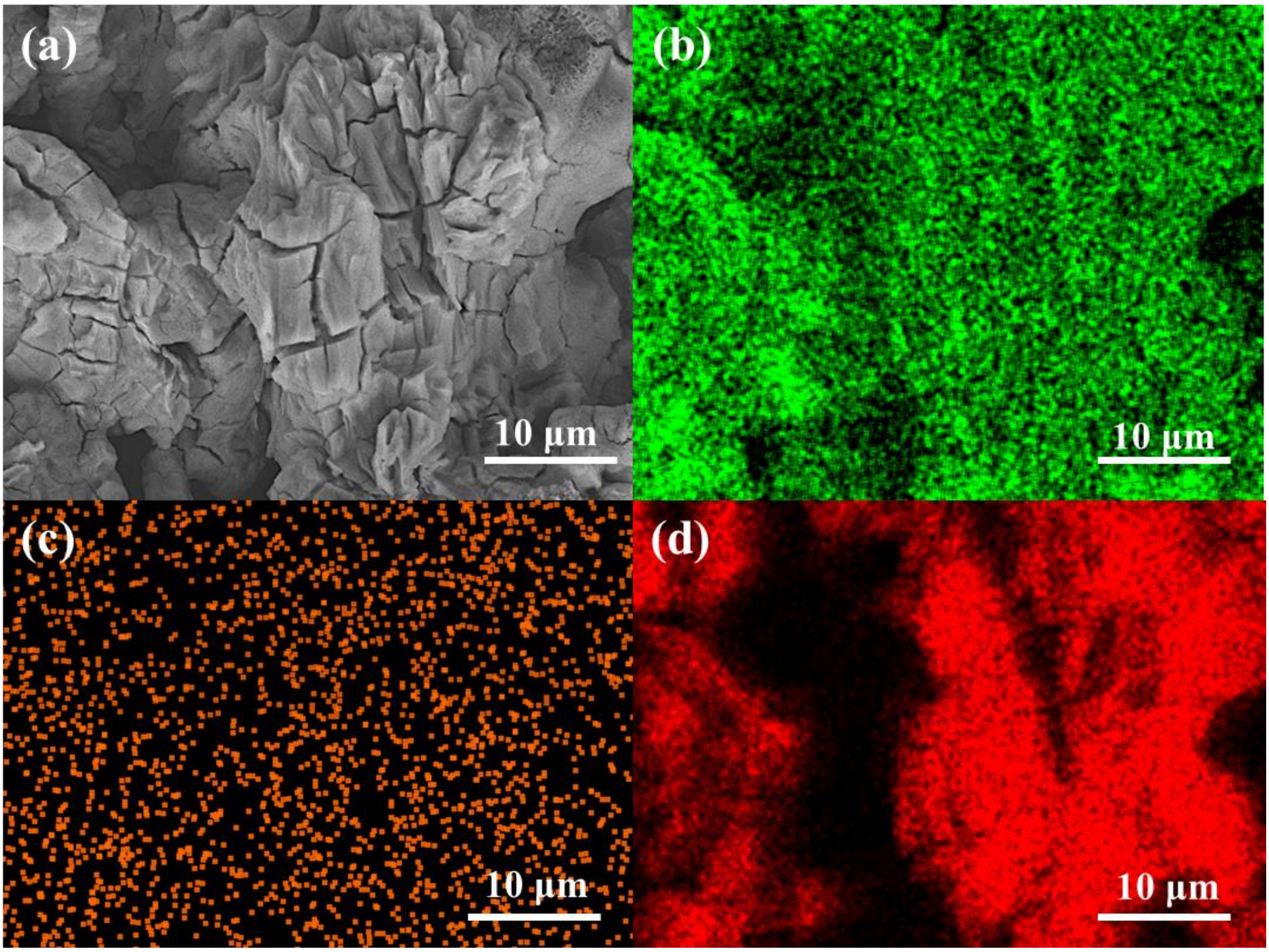

Figure S46. The SEM image (a) and the respective EDX mapping of $MnGa_4$ after OER CV cycles (b-d). The spectra exhibit drastic change than the original $MnGa_4$ structure. The mapping showed a slight loss of gallium (c) from the structure with the inclusion of oxygen (d) in the structure. This indicates under the prolonged electrolysis, the loss of Ga from the materials takes place, which is likely to go deeper beyond the particle surface and completely transforms the original $MnGa_4$ structure to manganese hydroxides. Detailed atomic % of the distribution of the elements obtained by EDX is listed in Table S1.

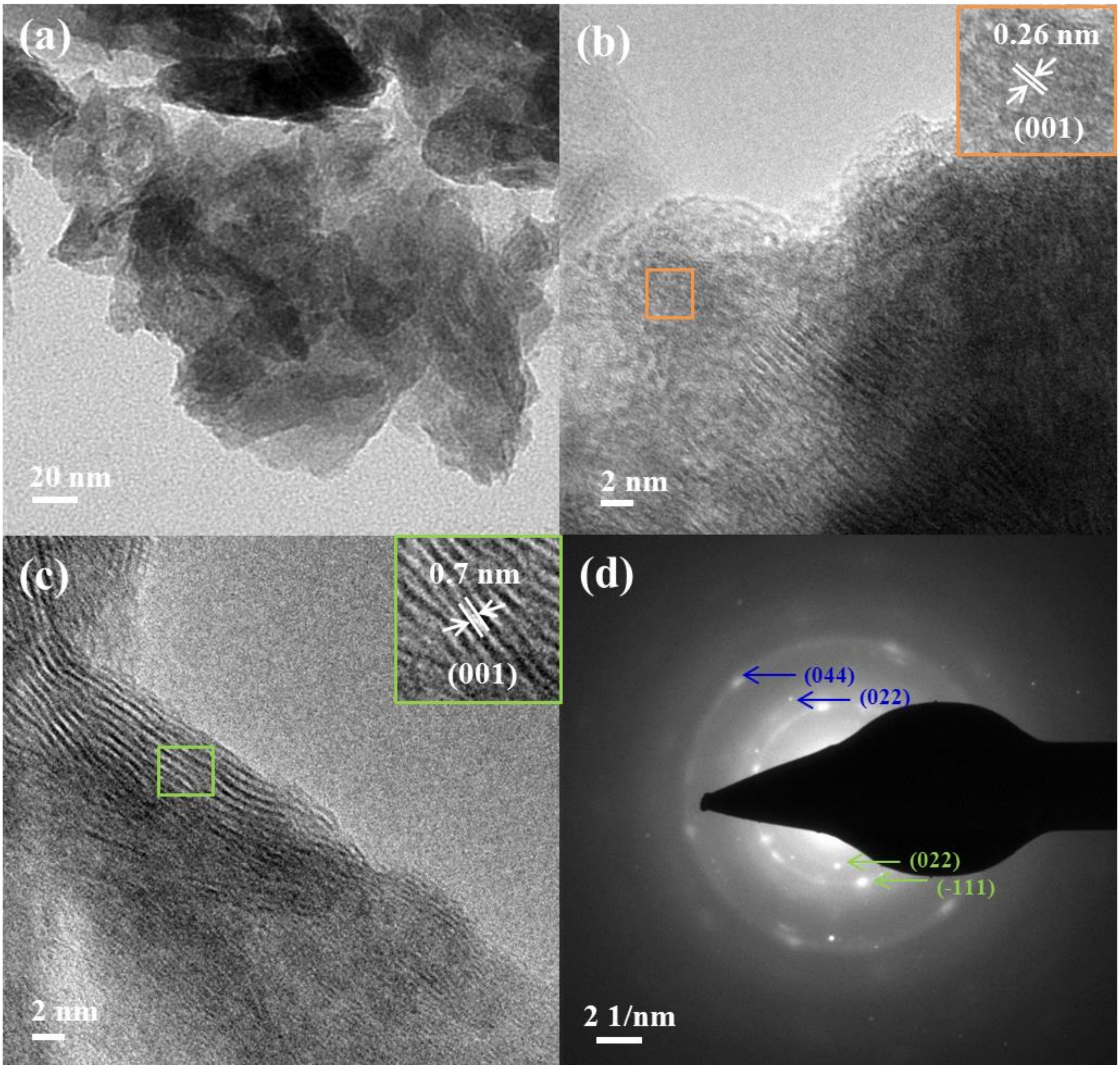

Figure S47. (a) TEM (a) HR-TEM images (b, c) and SAED (d) pattern of $MnGa_4$ after OER CV cycles. The TEM images (a) showed a loss of Ga from the particles transforming $MnGa_4$ partially into a hollow porous nanostructure. The nanostructure (b, c) suggested a lattice spacing of 0.7 nm and ~0.26 nm that can be assigned to the (001) plane of (K-) birnessite δ-MnO_2 structure (JCPDS 80-1098 and either (301) planes of feitknechtite β-MnOOH (JCPDS 18-804) or (311) planes of hausmannite α-Mn_3O_4 (JCPDS 75-1560) structure. The attained SAED diffraction rings for the as-prepared $MnGa_4$ as well as δ-MnO_2 phase indicate only a partial transformation of the as-prepared phase under CV conditions.

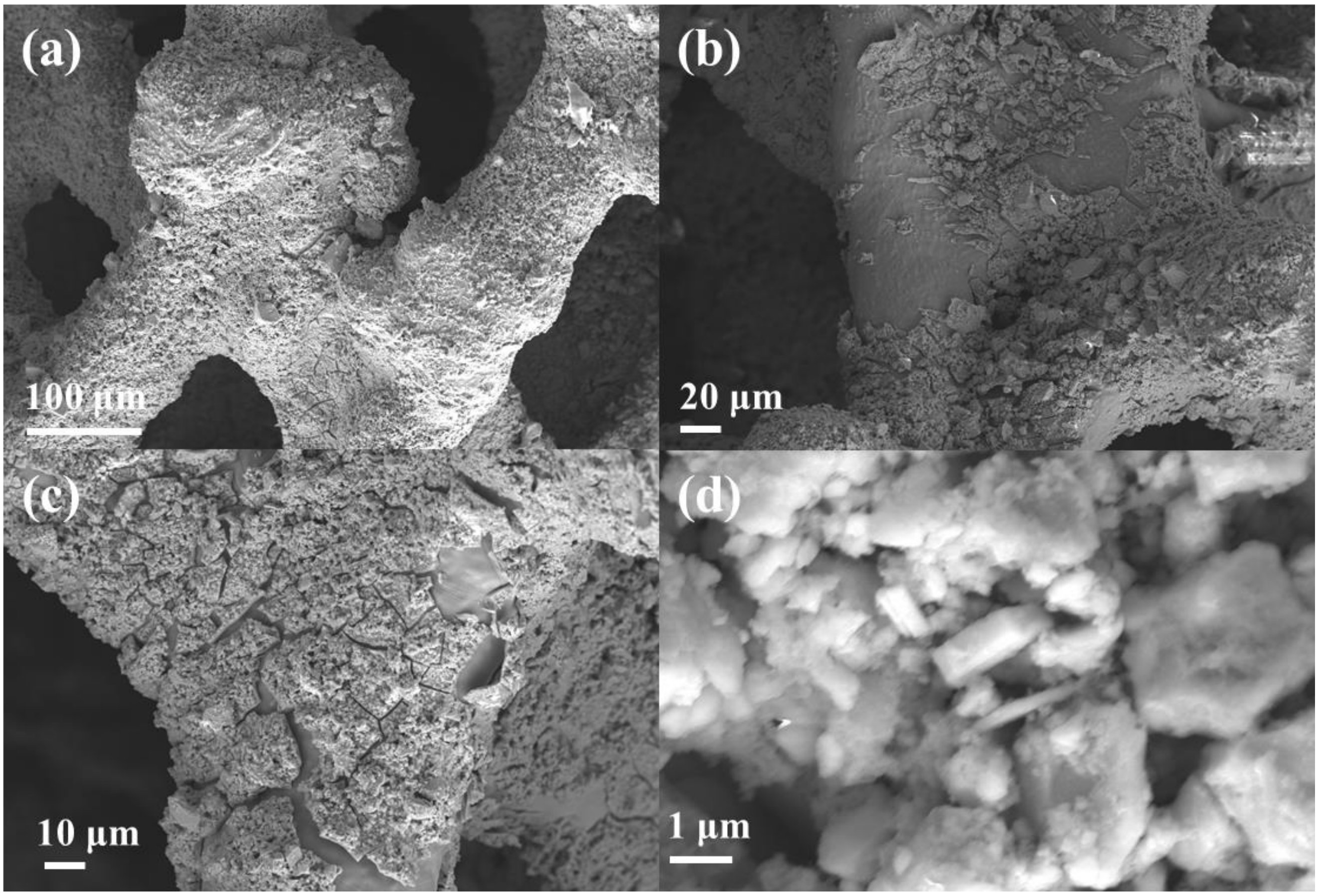

Figure S48. SEM images (a-d) of $MnGa_4$ films on NF with different magnifications where good coverage of material on NF was clearly observed

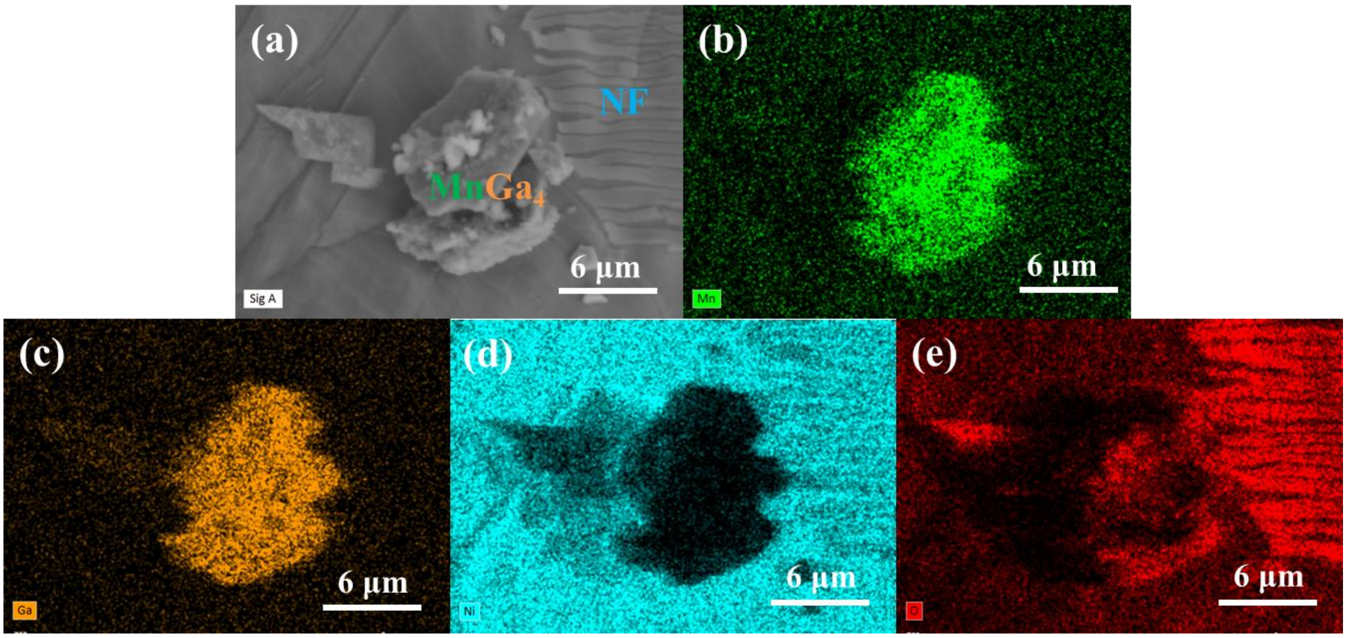

Figure S49. The SEM image (a) and the respective EDX mapping $MnGa_4$ films on NF (b-e) showing homogenously distribution of manganese (b) and gallium (c) on the particles while nickel (d) and oxygen (e) was only concentrated on the NF.

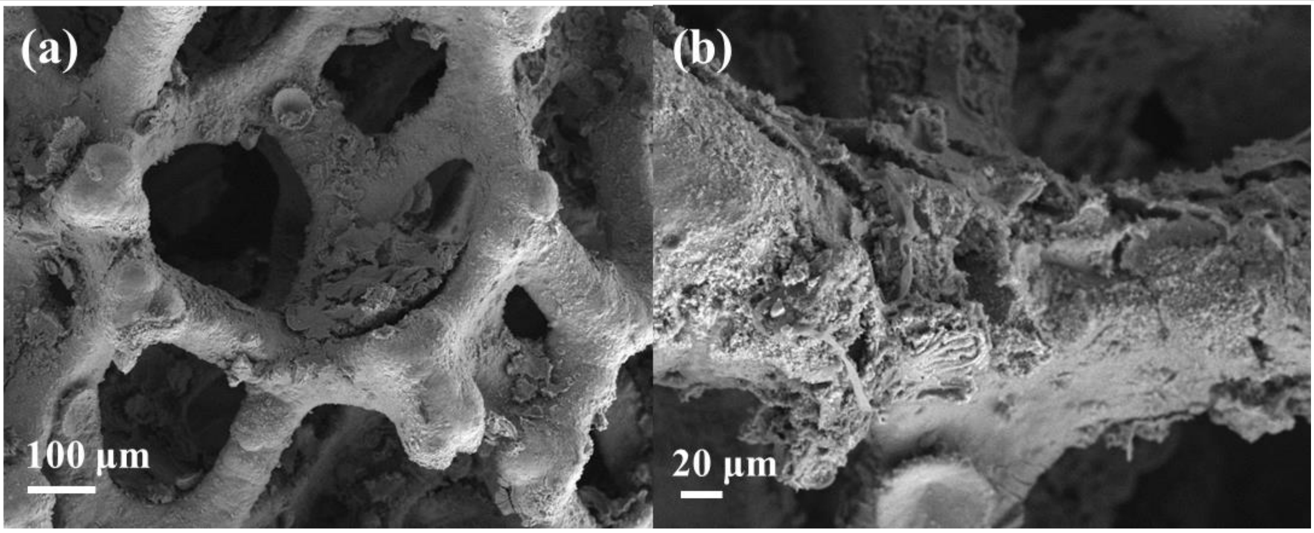

Figure S50. SEM images (a-d) of $MnGa_4$ films on NF after OER CA (24 h) with different magnifications showing the transformation of the as-synthesized material.

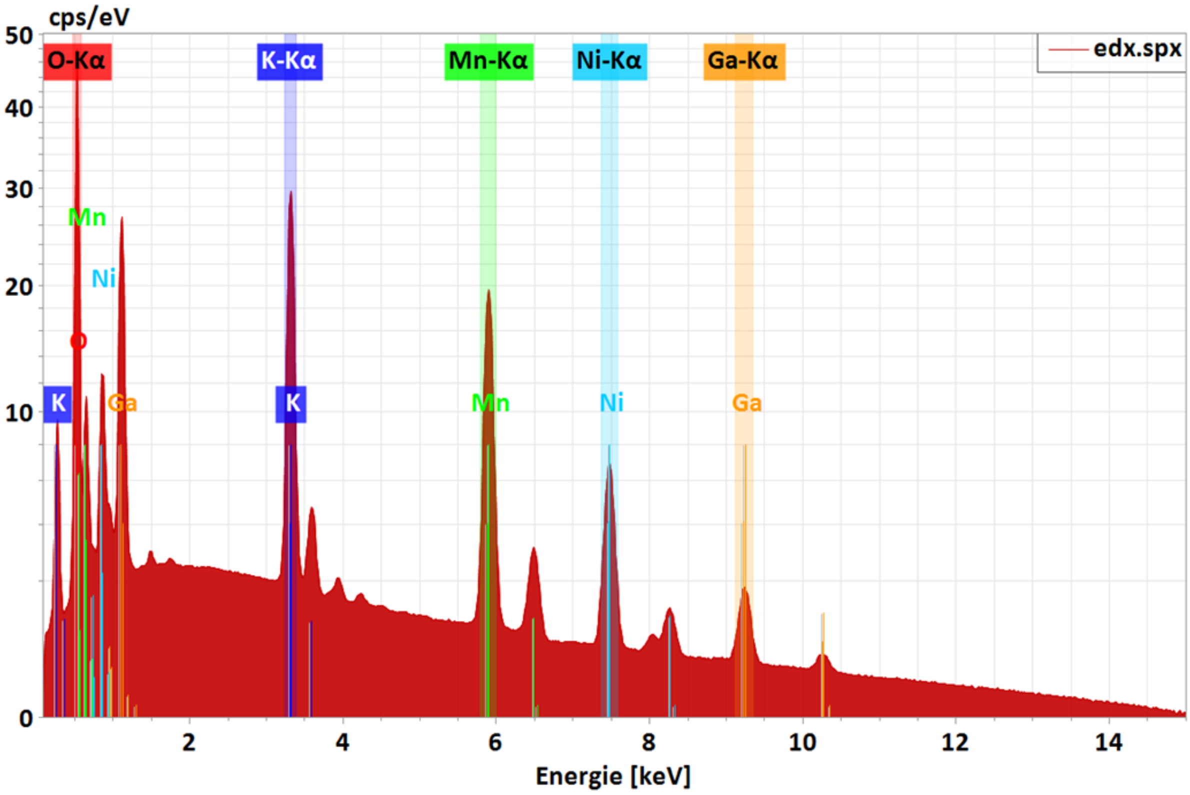

Figure S51. The EDX mapping spectrum of $MnGa_4$ films on NF after OER CA

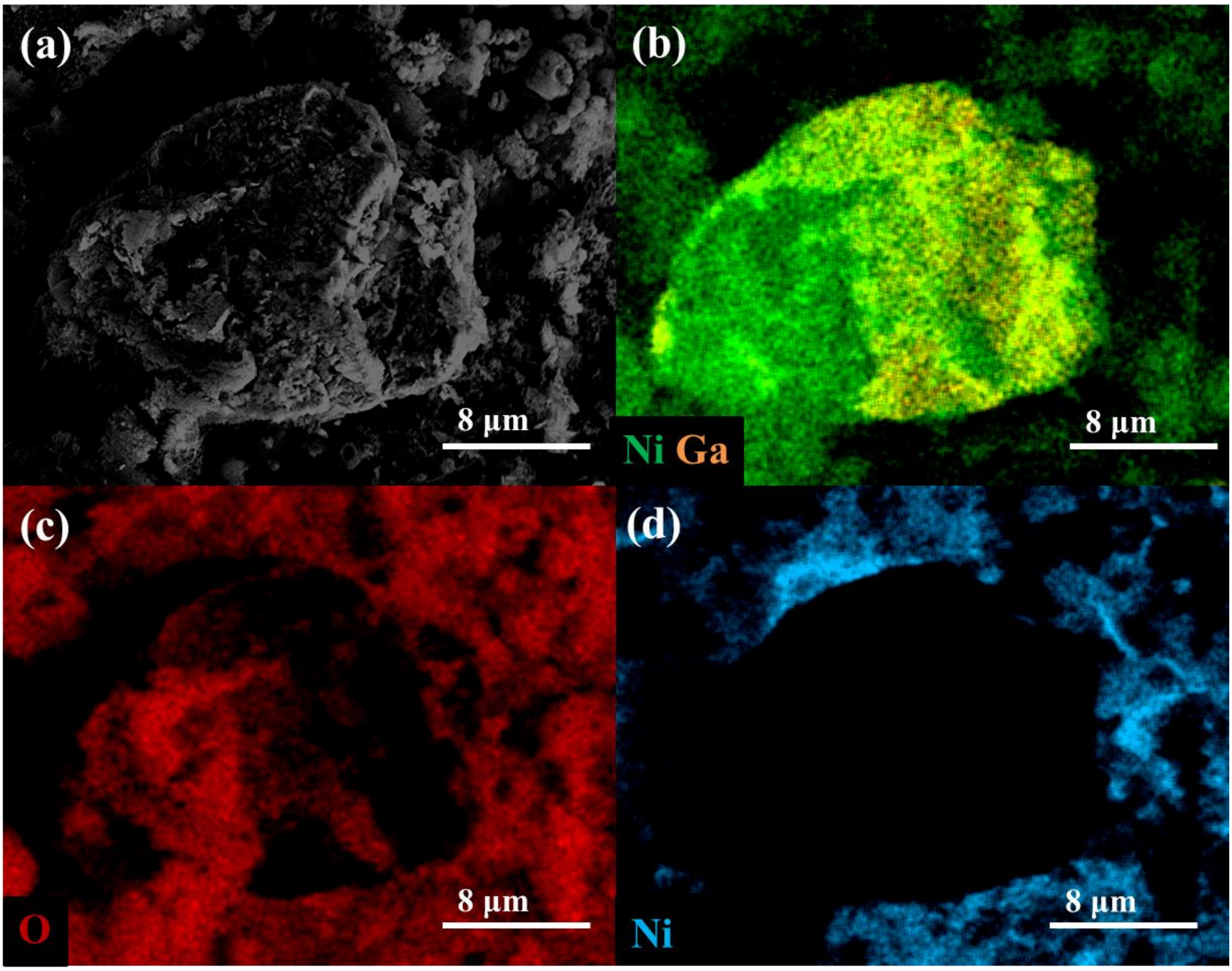

Figure S52. The SEM image (a) and the respective EDX mapping of sheared $MnGa_4$ films after OER CA on NF (b-d). The spectra exhibit homogenously distributed manganese (b) and oxygen (c) while the gallium (orange spots in b) atoms mostly disappeared from the structure indicating corrosion-induced transformation of the particles under OER conditions. Notably, the EDX mapping on the selected particle also ruled out the Ni incorporation in the transformed active material.

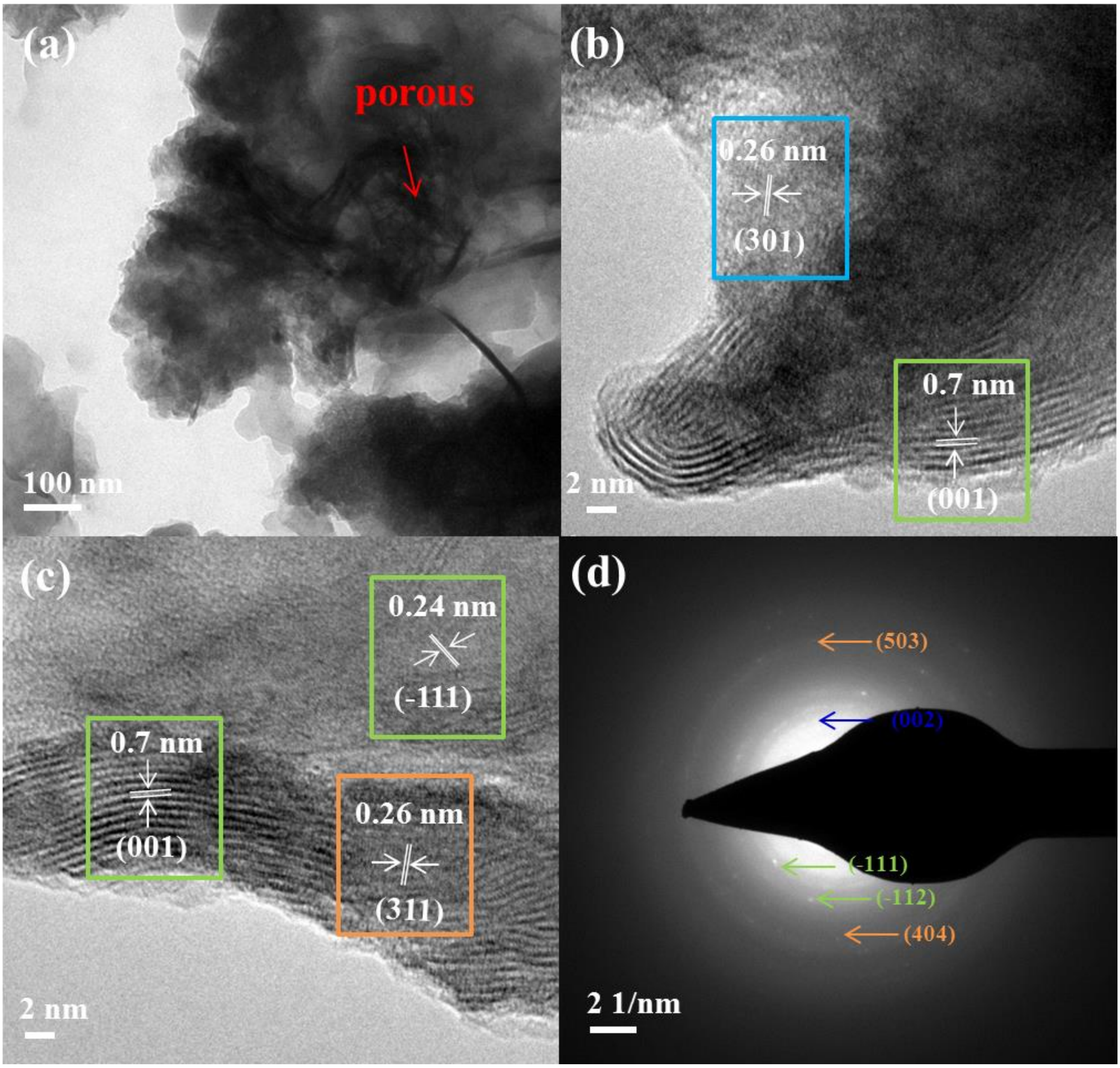

Figure S53. (a) TEM (a) HR-TEM images (b, c) and SAED pattern of $MnGa_4$ deposited on NF after OER CA (24 h). This was done to rule out the Ni incorporation in $MnGa_4$ from NF and therefore, $MnGa_4$/NF films after OER CA were sonicated and TEM was conducted on the obtained particles. The TEM images (a) showed a severe loss of Ga from the particles transforming $MnGa_4$ completely into a hollow porous nanostructure. A closer look at the nanostructures (b, c) suggested a lattice spacing of 0.7 and 0.24 nm that can be assigned to the (001) and (-111) plane of (K-) birnessite δ-MnO_2 structure (JCPDS 80-1098). Furthermore, the distance of ~0.26 nm was also attained that could be ascribed to either (301) planes of feitknechtite β-MnOOH (JCPDS 18-804) or (311) planes of hausmannite α-Mn_3O_4 (JCPDS 75-1560) structure. The obtained TEM results are consistent with the PXRD pattern. No nickel contained particles were observed, which was also confirmed by the EDX mapping (see Figure S51). The SAED pattern indicated crystalline particles with diffraction rings associated with δ-MnO_2 (green indices), MnOOH (orange indices) and α-Mn_3O_4 (blue indices), respectively.

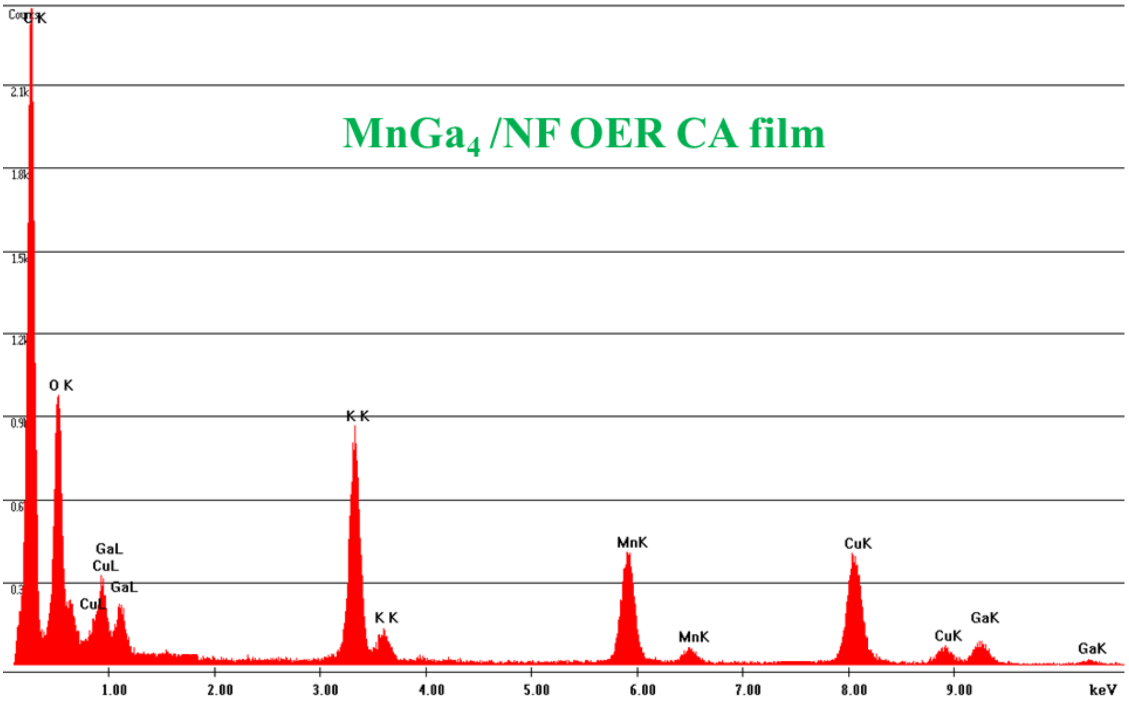

Figure S54. The EDX analysis of particles (obtained from sonication) $MnGa_4$ OER CA deposited on NF after (24 h). The weakening of Ga peaks and enhancement in Mn peaks were observed that are indicative of severe loss of Ga in OER conditions. The presence of K is due to the electrolyte (in the form of KOH, and the peaks for copper can be unambiguously correlated to the TEM grid (carbon film on 300 mesh Cu-grid).

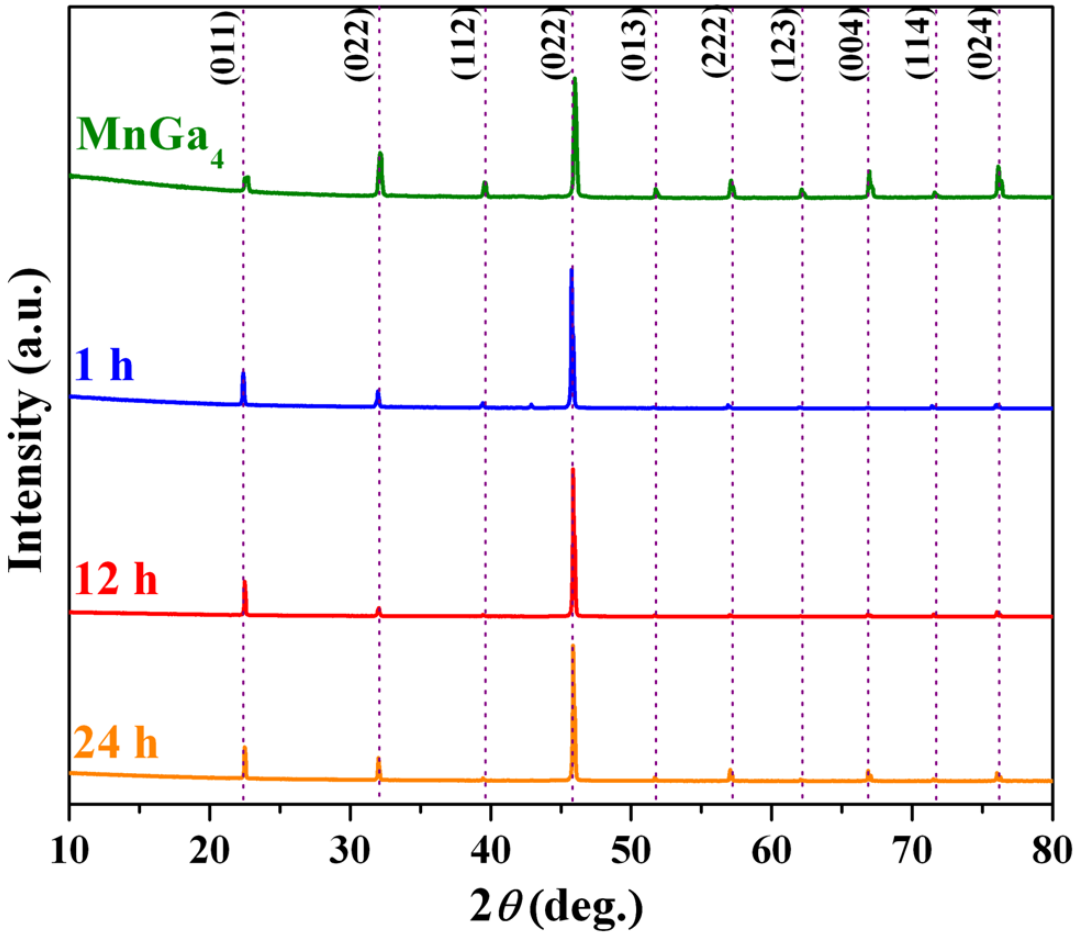

Figure S55. The chemical stability of $MnGa_4$ in 1 M aqueous KOH solution. 30 mg of $MnGa_4$ powder was suspended in 10 mL of KOH solution and stirred for various time intervals (1, 12 and 24 h). No change the relative PXRD pattern was observed. The ICP-AES values obtained from the aqueous KOH solution are presented in Table 4.

Table S5: Determination of percentage of gallium loss in $MnGa_4$ after the suspension of the as-prepared powder in 1 M aqueous KOH solution at various time intervals. Each experiment was conducted thrice independently, and the average value is presented.

	Mn (%)	Ga (%)
$MnGa_4$	0	0
2 h	0.067	4,9
12 h	0.074	7.4
24 h	0.081	11.3

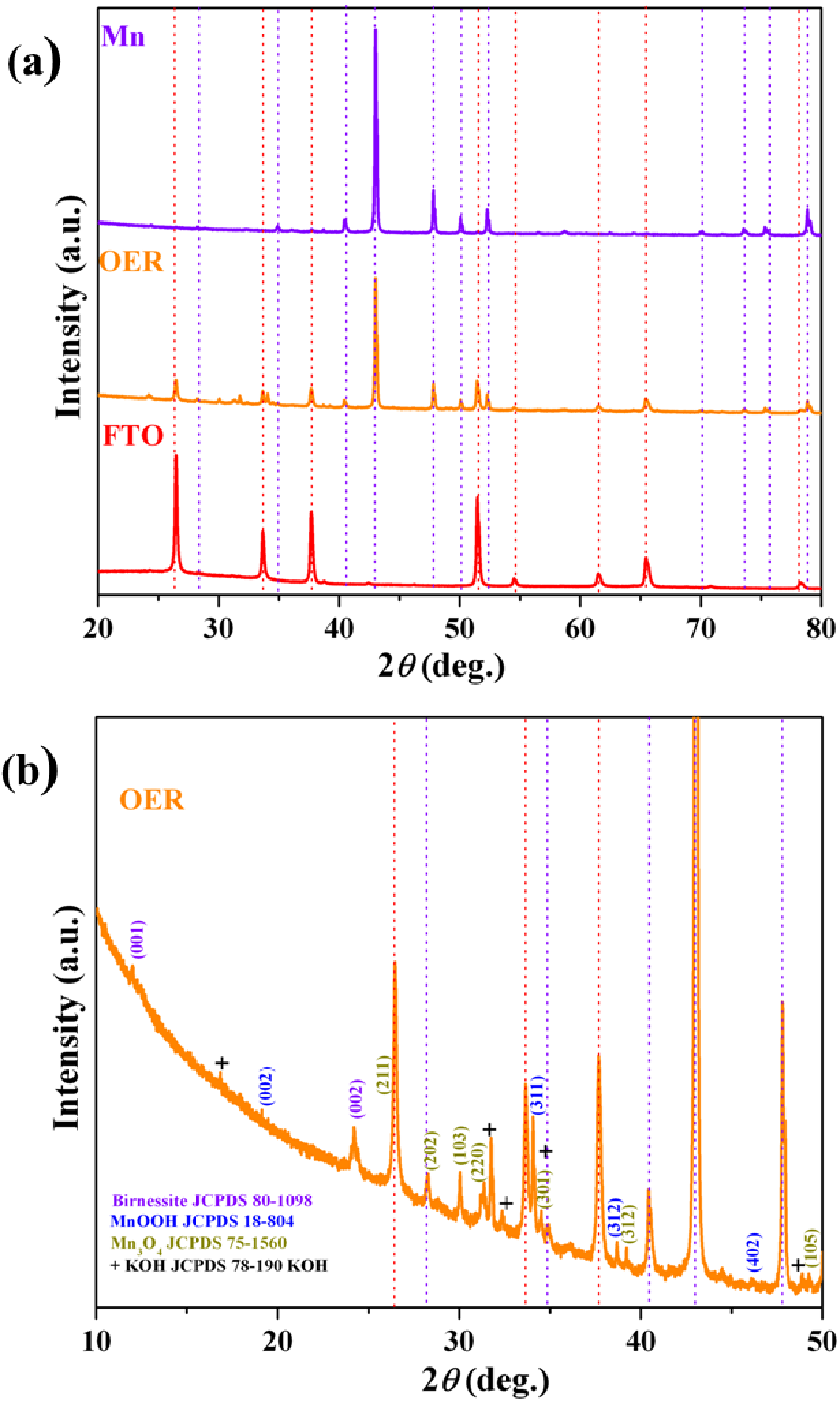

Figure S56. (a) The PXRD pattern of Mn, Mn after OER CA (24 h) and bare FTO. The reflections of Mn and bare FTO are marked in broken purple and red lines. The PXRD pattern of Mn after OER CA experiments was indexed carefully (b) that showed the presence of a slight amount of (K-) birnessite δ-MnO_2 (JCPDS 80-1098), feitknechtite β-MnOOH (JCPDS 18-804) and hausmannite α-Mn_3O_4 (JCPDS 75-1560). Apart from manganese oxides, some amount of KOH was also identified.

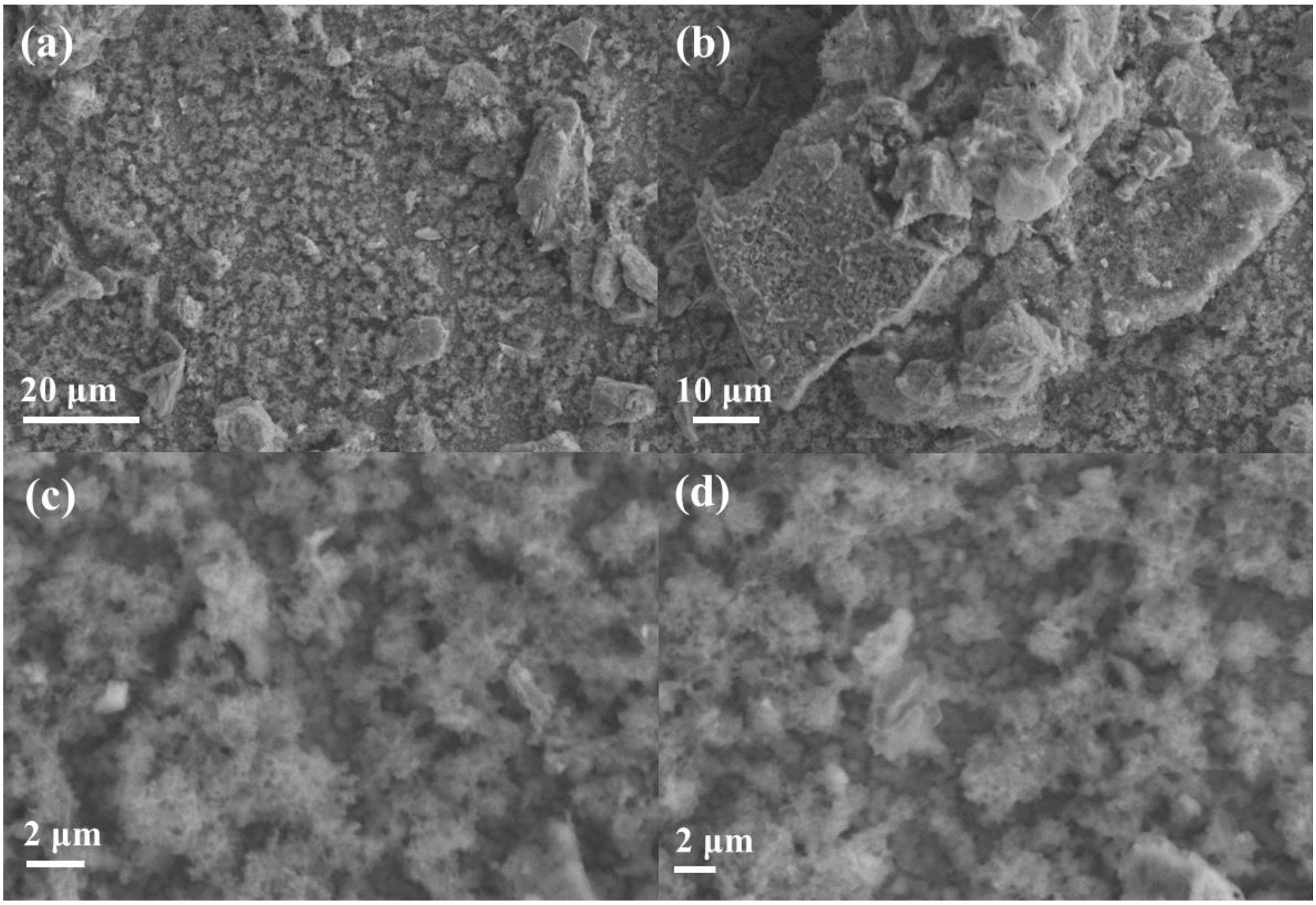

Figure S57. SEM images (a-d) of Mn films after OER showing different magnifications. The corrosion effect on Mn films is also obviously revealed the formation of MnO_x on the surface of the particles.

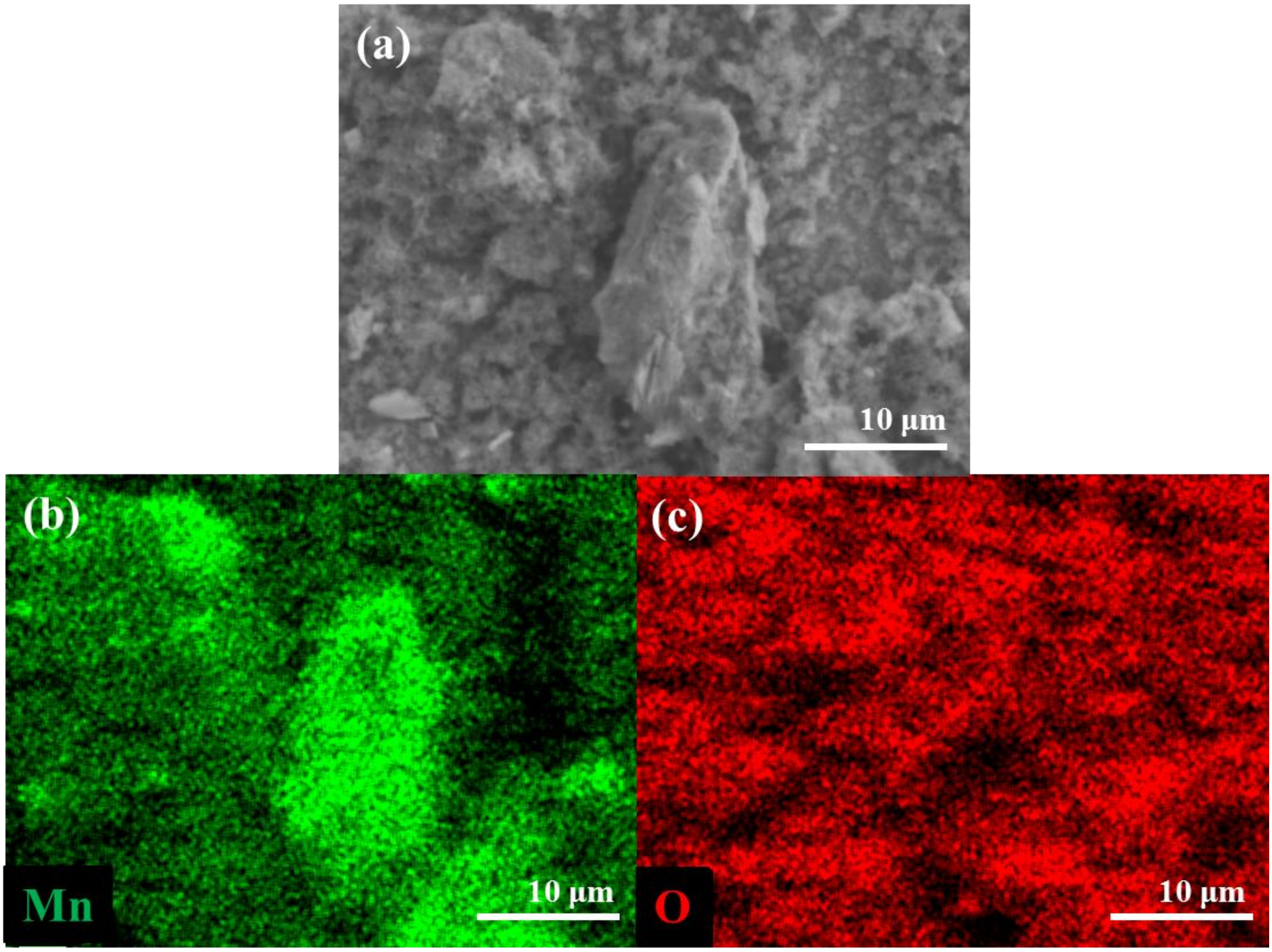

Figure S58. The SEM image (a) and the respective EDX mapping of Mn after OER CA (b-c). The spectra exhibit a change in the structure where the manganese (b) and oxygen (c) were homogeneously distributed within the particles. Detailed atomic % of the distribution of the elements obtained by EDX is listed in Table S1.

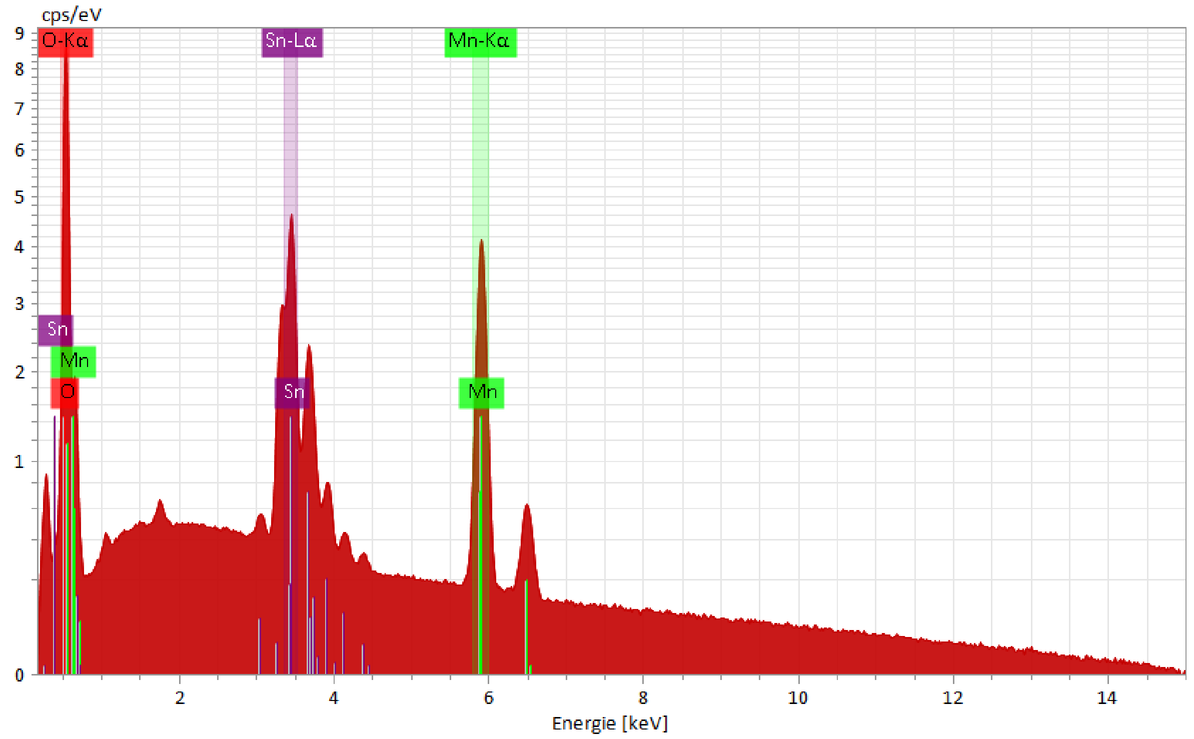

Figure S59. The EDX mapping spectrum of Mn.

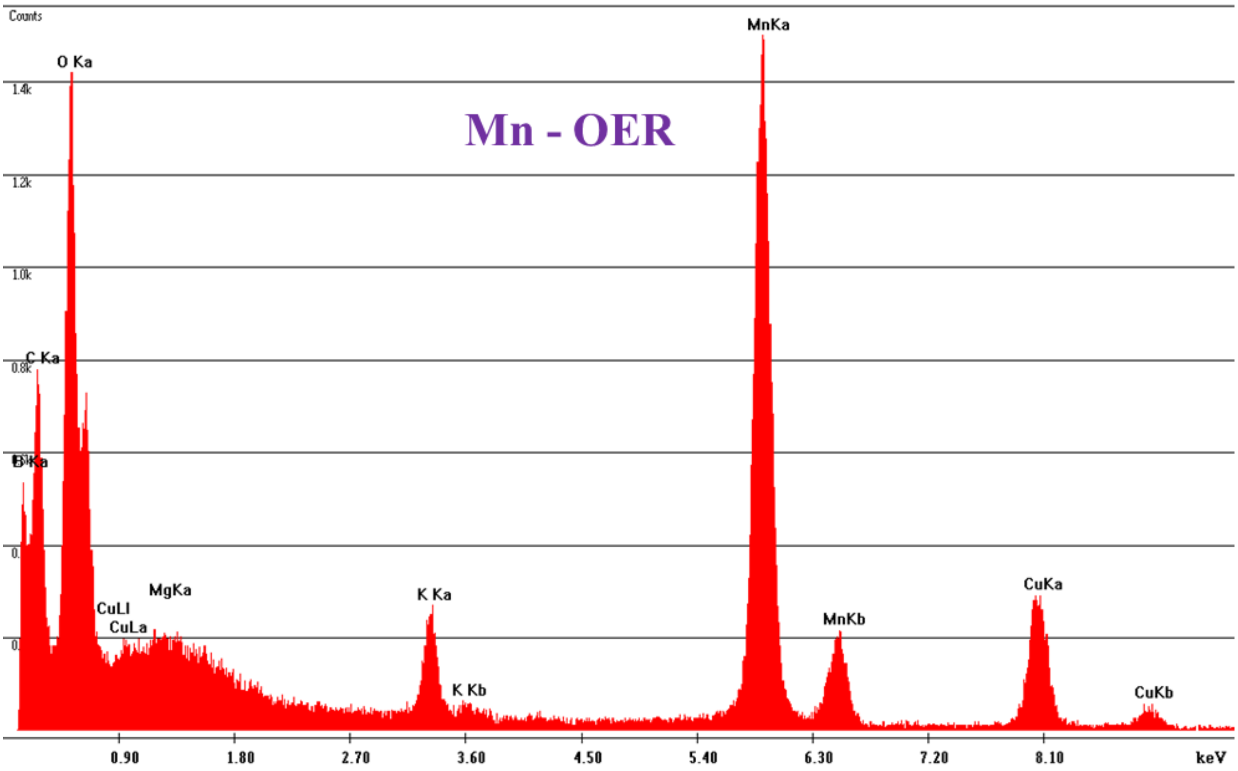

Figure S60. The EDX analysis of Mn after OER CA (24 h). The presence of K is due to the electrolyte (in the form of KOH), and the peaks for copper can be unambiguously correlated to TEM grid (carbon film on 300 mesh Cu-grid).

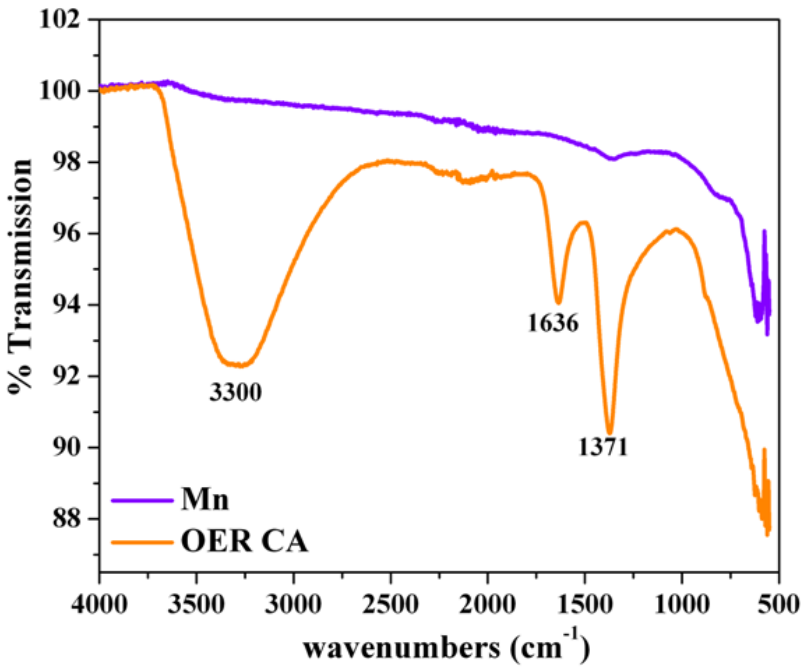

Figure S61. The FTIR spectra of Mn and the films after OER CA. After the OER, similar to $MnGa_4$, a broad band was observed at ~3330 cm^{-1} which is due to stretching vibrations of interlayer water molecules whereas the band at ~1640 cm^{-1} could be assigned to the bending vibration of H_2O and structural OH groups.[26] Bands around ~700 cm^{-1} and lower are characteristic bands of manganese oxides.[26b,28]

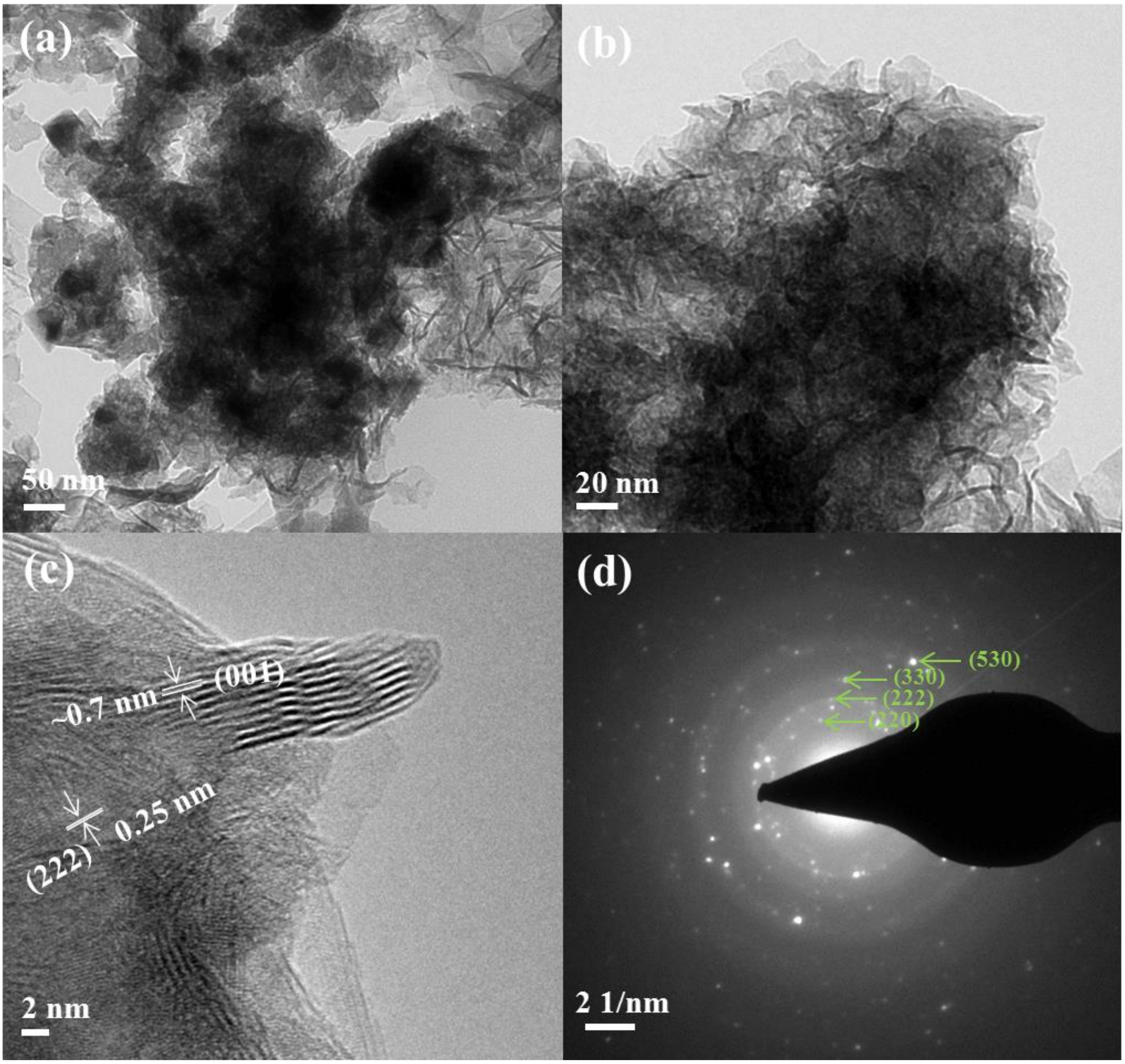

Figure S62. (a) TEM (a, b) HR-TEM images (c) and SAED pattern of Mn after OER CA (24 h). The TEM images (a, b) showed strong corrosion of Mn under applied oxidation potential in alkaline media. Clear large lattice fringes of a distance 0.7 nm corresponding to (001) planes of (K-) birnessite δ-MnO_2 (JCPDS 80-1098) structure was also obtained at the edge of the particles which very similar to the case of OER CA films of $MnGa_4$. As expected from PXRD, a spacing of 0.25 nm can either be assigned to either feitknechtite β-MnOOH (JCPDS 18-804) or hausmannite α-Mn_3O_4 (JCPDS 75-1560) phases. The SAED pattern (d) confirmed the presence of crystalline Mn phase at the core of the particles.

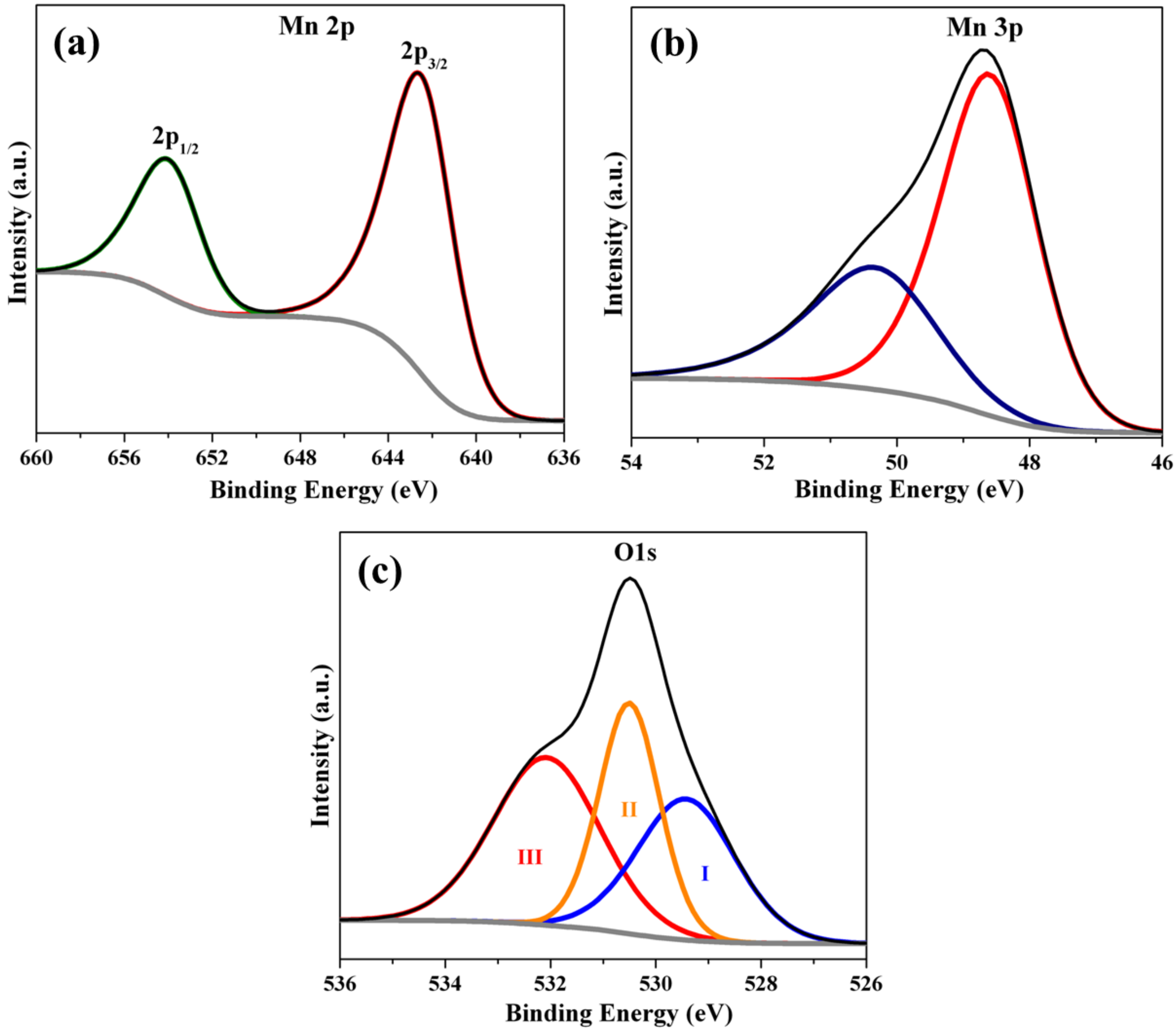

Figure S63. The high-resolution deconvoluted (a) Mn 2p, (b) Mn 3p, (c) O1s XPS spectra of Mn after OER CA (24 h). Similar to initial Mn, the Mn $2p_{3/2}$ and Mn $2p_{1/2}$ spectra of OER CA did not change and showed two at the binding energy of 642.3 eV and 654.2 eV (a) that is approximately close to the oxidation state of Mn^{IV}.[16] The Mn 3p spectra were deconvoluted into two peaks. The peak at the binding energy of ~48.5 eV corresponds to the oxidation state of Mn^{III} while the peak at 50 eV is due to the Mn^{IV}.[16c,17] The O1s XPS spectra could be deconvoluted into three peaks (I, II and III) where the small peak at 529.5 is due to the formation of manganese oxide (MnO_x) whereas the peaks at 530.8 eV and a broad peak at ~533 eV can be ascribed to hydroxylation (-OH/-OOH) and adsorbed water onto the surface.[5,7d,19b-e,20]

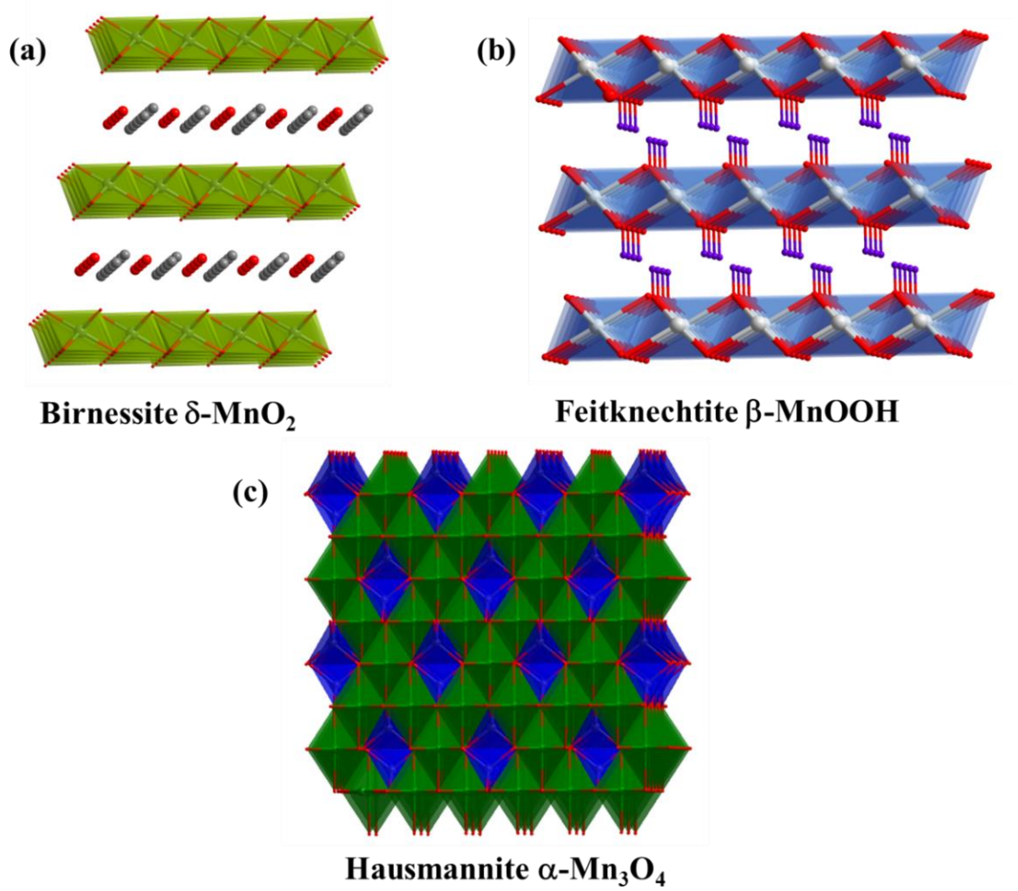

Figure S64. The crystal structure of (a) (K-) birnessite δ-MnO_2, (b) feitknechtite β-MnOOH and (c) hausmannite α-Mn_3O_4. The birnessite δ-MnO_2 (a) crystallizes in the monoclinic system (space group *A*2/*m*) and contain corner-sharing Mn octahedral layers (chartreuse octahedra) with a typical interlayer distance of ~7 Å occupied by interstitial disordered water/cationic sites (red and gray spheres).[10a,29] Some of the Mn cations within the MnO_2 layer are reduced from Mn^{IV} to Mn^{III}. The Mn^{III} ions are situated above or below the interlayer bonded through corner-sharing bridges.[29a,30] The feitknechtite β-MnOOH (b) belongs to the hexagonal system (space group *P*-3*m*) and has a layered structure where Mn is trivalent (blue octahedra), and one-half of the O atoms are replaced by hydroxyl anions (purple spheres).[31] The hausmannite α-Mn_3O_4 is a tetragonal (space group $I4_1/amd$) spinel with the general formula AB_2O_4 ($Mn^{II}Mn_2^{III}O_4$) structure in which the Mn atoms are placed among the tetrahedral (A) and octahedral (B) sites.[6] The octahedral sites (green octahedra) are close to each other sharing edges, but tetrahedral sites (blue octahedra) share only corners with the octahedral sites. It has been previously shown that β-MnOOH is a metastable phase and is an intermediate phase between α-Mn_3O_4 and δ-MnO_2.[32] Recent computational studies have also confirmed the formation of the nascent δ-MnO_2 layer produced in situ from spinel α-Mn_3O_4 under electrochemical conditions.[30c] Therefore, from our experimental findings, it is reasonable to expect that the $MnGa_4$ phase was electroconverted first to α-Mn_3O_4 and then to δ-MnO_2 through the intermediate β-MnOOH. Although it is expected δ-MnO_2 to be the active phase for durable water oxidation, however, the contribution of α-Mn_3O_4 and β-MnOOH for water oxidation during transformation cannot be ruled out completely.

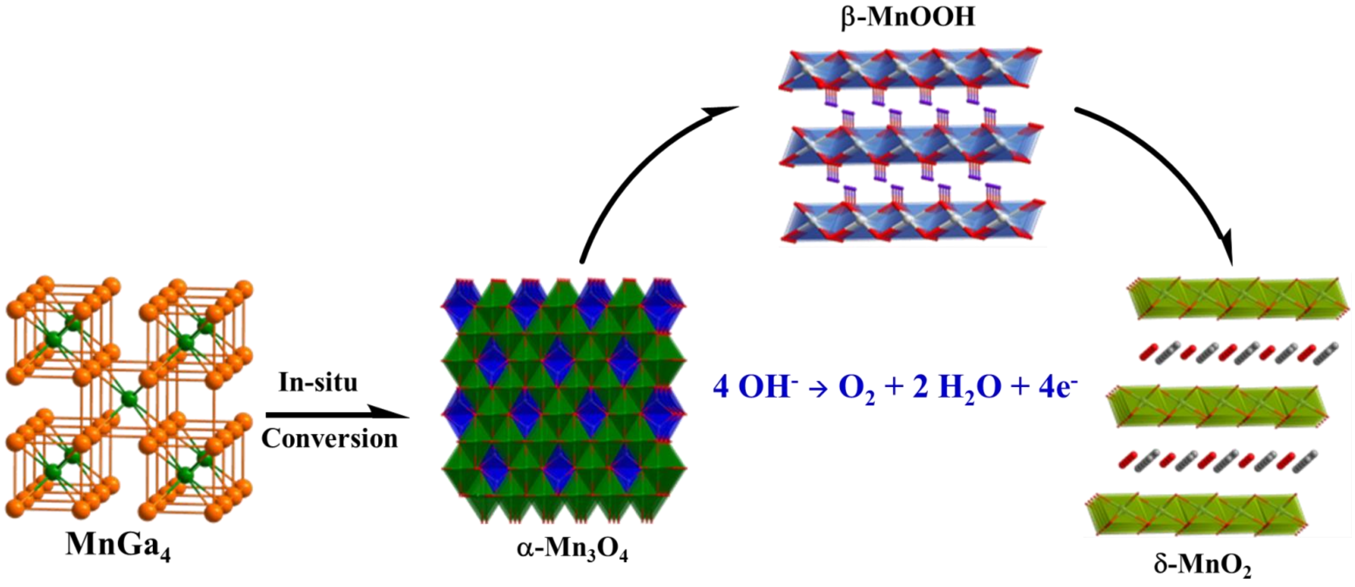

Scheme S1. Electroconversion of $MnGa_4$ to α-Mn_3O_4 which was finally transformed into the most thermodynamically stable and active phase of δ-MnO_2 via the intermediate β-MnOOH to drive the water oxidation reaction.

Table S6. The comparison of OER overpotentials of $MnGa_4$ with other superior selected non-noble transition metal-based catalysts in aqueous 1 M KOH.

Catalyst	Current density ($mAcm^{-2}$)	Overpotential (mV)	Reference
**$MnGa_4$/NF**	**10**	**291±2**	**This work**
	**100**	**402±9**	**This work**
**Mn/NF**	**10**	**425±5**	**This work**
	**100**	**560±12**	**This work**
**Bare NF**	**10**	**496±19**	**This work**
**RuO_2/NF**	**10**	**293**	**This work**
	**100**	**420**	**This work**
**IrO_2/NF**	**10**	**310**	**This work**
	**100**	**445**	**This work**
**Pt wire**	**10**	**-**	**This work**
$CoSn(OH)_6$/CC	10	274	[33]
p-SnNiFe/GC	10	350	[34]
Ni_2P/Ni_5P_4-NiP_2/NF	191	350	[35]
Ni_2P/FTO	10	400	[20]
Ni_2P/FTO	10	500	[20]
Ni_2P/Ni/NF	10	200	[36]
Ni_xP_y-325	10	320	[37]
Ni_2P/GC	10	290	[38]
Ni_5P_4 films	10	290	[39]
Ni-P film	10	344	[40]
Co-P/Cu	10	345	[41]
NiCo/NS	10	334	[42]
NiCo LDH	10	367	[43]
$Ni_xCo_{3-x}O_4$ NWs/Ti	10	370	[44]
Ni_3S_2/NF	10	250	[45]
NiFe/NF	10	215	[46]
$Ni_{1-x}Fe_x$ NC/GC	10	330	[31c]
Co_3O_4/ $NiCo_2O_4$ DSNCs	10	340	[47]
CoCo LDH	10	393	[42]
CoO_x/CN	10	260	[48]
FeOOH/CeO_2/NF	10	250	[49]
N-G/CoO	10	340	[50]
Co_3O_4/N-rmGO	10	310	[51]
$CoFeO_x$	10	360	[52]
$NiFeO_x$	10	350	[52]
FeCo-Co_4N/N-C	10	280	[53]
MoO_2/NF	10	250	[54]
MoO_2/NF compact	10	500	[54]
FeP/Au	10	290	[55]
FeP RGO/Au	10	260	[55]

Table S7. The comparison of OER overpotentials of $MnGa_4$ with other superior selected manganese oxide catalysts in aqueous 1 M KOH.

Catalyst	Current density ($mA \cdot cm^{-2}$)	Overpotential (mV)	Reference
**$MnGa_4$/NF**	**10**	**291±2**	**This work**
	**100**	**402±9**	**This work**
**Mn/NF**	**10**	**425±5**	**This work**
	**100**	**560±12**	**This work**
**Bare NF**	**10**	**496±19**	**This work**
**RuO_2/NF**	**10**	**293**	**This work**
	**100**	**420**	**This work**
**IrO_2/NF**	**10**	**310**	**This work**
	**100**	**445**	**This work**
**Pt wire**	**10**	**-**	**This work**
Mn_3N_2/FTO	10	390	[56,57]
Mn_3N_2/NF	10	270	[56,57]
$LiMn_{0.25}Co_{1.75}O_4$	10	430	[58]
Au(50%)-MnO_x	10	400	[59]
MnO_2	10	500	[60]
Mn_3O_4/NF	10	287	[61]
MnO_x	10	573	[62]
Mn_2O_3	10	350	[63]
Mn_3O_4 (115 m^2 g^{-1})	10	480	[63]
Mn_3O_4 (30 m^2 g^{-1})	10	510	[63]
Mn_3O_4@$Co_xMn_{3-x}O_4$ NF	10	284	[64]
Mn_5O_8	10	490	[63]
ALD MnO_2	10	619	[65]
ALD Mn_2O_3	10	801	[65]
Mn_2O_3 (at 450°C)	10	387	[66]
Mn_2O_3 (at 350°C)	10	426	[66]
Mn_2O_3 (at 550°C)	10	407	[66]
A-CoMnO	10	340	[67]
α-Mn_2O_3	10	340	[68]
porous β-MnO_2	10	450	[69]
Mn-380	10	427	[70]
Mn-450	10	470	[70]
Mn-575	10	570	[70]
$Fe_{1.8}Mn_{0.2}P$	10	620	[71]
$Fe_{1.3}Mn_{0.7}P$	10	480	[71]
Mn_2O_3	10	570	[72]
Birnessite	10	700	[73]
Ni^{2+}/Birnessite	10	400	[73]
$Mn_{25}Ru_{75}$ @ 450	10	259	[74]
$Mn_{90}Ru_{10}$ @ 350	10	312	[74]
Mn_{100} @ 350	10	428	[74]
MnO_x/NiFe-LDH NF	10	174	[75]

References

[1] C. Schlesiger, L. Anklamm, H. Stiel, W. Malzer, B. Kanngiesser, *J. Anal. At. Spectrom.* **2015**, *30*, 1080-1085.
[2] M. Dimitrakopoulou, X. Huang, J. Krohnert, D. Teschner, S. Praetz, C. Schlesiger, W. Malzer, C. Janke, E. Schwab, F. Rosowski, H. Kaiser, S. Schunk, R. Schlogl, A. Trunschke, *Faraday Discuss.* **2018**, *208*, 207-225.
[3] B. Ravel, M. Newville, *J. Synchrotron Radiat.* **2005**, *12*, 537-541.
[4] V. Y. Verchenko, A. A. Tsirlin, D. Kasinathan, S. V. Zhurenko, A. A. Gippius, A. V. Shevelkov, *Phys.l Rev. Mater.* **2018**, *2*, 125118.
[5] C. Walter, P. W. Menezes, S. Orthmann, J. Schuch, P. Connor, B. Kaiser, M. Lerch, M. Driess, *Angew. Chem. -Int. Ed.* **2018**, *57*, 698-702.
[6] P. W. Menezes, A. Indra, P. Littlewood, M. Schwarze, C. Gobel, R. Schomacker, M. Driess, *ChemSusChem* **2014**, *7*, 2202-2211.
[7] a) F. Bozza, R. Polini, E. Traversa, *Fuel Cells* **2008**, *8*, 344-350; b) L. Besra, M. Liu, *Prog. Mater. Sci.* **2007**, *52*, 1-61; c) J. Pfrommer, A. Azarpira, A. Steigert, K. Olech, P. W. Menezes, R. F. Duarte, X. X. Liao, R. G. Wilks, M. Bar, T. Schedel-Niedrig, M. Driess, *ChemCatChem* **2017**, *9*, 672-676; d) P. W. Menezes, C. Panda, S. Garai, C. Walter, A. Guiet, M. Driess, *Angew. Chem. -Int. Ed.* **2018**, *57*, 15237-15242.
[8] a) Y. Yang, J. X. Huang, J. Zeng, J. Xiong, J. B. Zhao, *ACS Appl. Mater. Interfaces* **2017**, *9*, 32801-32811; b) Y. Yang, J. Q. Li, D. Q. Chen, T. Fu, D. Sun, J. B. Zhao, *ChemElectroChem* **2016**, *3*, 757-763.
[9] S. Anantharaj, S. R. Ede, K. Karthick, S. S. Sankar, K. Sangeetha, P. E. Karthik, S. Kundu, *Energy Environ. Sci.* **2018**, *11*, 744-771.
[10] a) Q. Kang, L. Vernisse, R. C. Remsing, A. C. Thenuwara, S. L. Shumlas, I. G. McKendry, M. L. Klein, E. Borguet, M. J. Zdilla, D. R. Strongin, *J. Am. Chem. Soc.* **2017**, *139*, 1863-1870; b) C. C. L. McCrory, S. Jung, I. M. Ferrer, S. M. Chatman, J. C. Peters, T. F. Jaramillo, *J. Am. Chem. Soc.* **2015**, *137*, 4347-4357.
[11] H. F. Liang, A. N. Gandi, D. H. Anjum, X. B. Wang, U. Schwingenschlogl, H. N. Alshareef, *Nano Lett.* **2016**, *16*, 7718-7725.
[12] C. C. L. McCrory, S. H. Jung, J. C. Peters, T. F. Jaramillo, *J. Am. Chem. Soc.* **2013**, *135*, 16977-16987.
[13] a) M. Tillard, C. Belin, *Intermetallics* **2012**, *29*, 147-154; b) U. Haussermann, P. Viklund, M. Bostrom, R. Norrestam, S. I. Simak, *Phys. Rev. B* **2001**, *63*, 125118.
[14] a) L. L. Xu, W. P. Liu, X. F. Li, S. D. Rashid, C. S. Shen, Y. Z. Wen, *RSC Adv.* **2015**, *5*, 12248-12256; b) T. N. Ye, S. Li, X. Y. Wu, M. Xu, X. Wei, K. X. Wang, H. L. Bao, J. Q. Wang, J. S. Chen, *Journal of Materials Chemistry C* **2013**, *1*, 4327-4333; c) C. Zhan, J. Lu, A. J. Kropf, T. P. Wu, A. N. Jansen, Y. K. Sun, X. P. Qiu, K. Amine, *Nature Communications* **2013**, *4*; d) C. E. Frey, F. Kwok, D. Gonzales-Flores, J. Ohms, K. A. Cooley, H. Dau, I. Zaharieva, T. N. Walter, H. Simchi, S. E. Mohney, P. Kurz, *Sust. Energy Fuels* **2017**, *1*, 1162-1170.
[15] a) A. Johannes, D. Salomon, G. Martinez-Criado, M. Glaser, A. Lugstein, C. Ronning, *Sci. Adv.* **2017**, *3*; b) G. R. Hearne, S. Bhattacharjee, B. P. Doyle, M. A. M. Ahmed, P. Musyimi, E. Carleschi, *Phys. Rev. B* **2018**, *98*.
[16] a) M. Huang, Y. X. Zhang, F. Li, Z. C. Wang, Alamusi, N. Hu, Z. Y. Wen, Q. Liu, *Sci. Rep.* **2014**, *4*; b) M. Oku, K. Hirokawa, S. Ikeda, *J. Electron Spectrosc. Rel. Phenom.* **1975**, *7*, 465-473; c) E. S. Ilton, J. E. Post, P. J. Heaney, F. T. Ling, S. N. Kerisit, *Appl. Sur. Sci.* **2016**, *366*, 475-485; d) M. C. Biesinger, B. P. Payne, A. P. Grosvenor, L. W. M. Lau, A. R. Gerson, R. S. Smart, *App. Sur. Sci.* **2011**, *257*, 2717-2730; e) H. W. Nesbitt, D. Banerjee, *Am. Mineral.* **1998**, *83*, 305-315.
[17] a) B. Gillot, S. Buguet, E. Kester, C. Baubet, P. Tailhades, *Thin Solid Films* **1999**, *357*, 223-231; b) L. Baggetto, N. J. Dudney, G. M. Veith, *Electrochim. Acta* **2013**, *90*, 135-147.
[18] D. Mondal, S. Banik, C. Kamal, P. Rajput, A. Thamizhavel, A. Banerjee, A. Chakrabarti, T. Ganguli, *arXiv:1803.06605* **2018**.
[19] a) Y. J. Lin, C. T. Lee, *Appl. Phys. Lett.* **2000**, *77*, 3986-3988; b) C. Panda, P. W. Menezes, C. Walter, S. L. Yao, M. E. Miehlich, V. Gutkin, K. Meyer, M. Driess, *Angew. Chem. Int. Ed.* **2017**, *56*, 10506-10510; c) C. Panda, P. W. Menezes, M. Zheng, S. Orthmann, M. Driess, *ACS Energy Lett.* **2019**, *4*, 747-754; d) S. Yao, V. Forstner, P. W. Menezes, C. Panda, S. Mebs, E. M. Zolnhofer, M. E. Miehlich, T. Szilv´asi, N. A. Kumar, M. Haumann, K. Meyer, H. Gr¨utzmacher, M. Driess, *Chem. Sci.* **2018**, *9*, 8590–8597; e) P. W. Menezes, A. Indra, C. Das, C. Walter, C. Gobel, V. Gutkin, D. Schmeisser, M. Driess, *ACS Catal.* **2017**, *7*, 103-109.
[20] P. W. Menezes, A. Indra, N. R. Sahraie, A. Bergmann, P. Strasser, M. Driess, *ChemSusChem* **2015**, *8*, 164-171.
[21] P. Casey, J. Bogan, B. Brennan, G. Hughes, *Appl. Phys. Lett.* **2011**, *98*.
[22] a) M. M. Najafpour, F. Rahimi, M. Fathollahzadeh, B. Haghighi, M. Holynska, T. Tomo, S. I. Allakhverdiev, *Dalt. Trans.* **2014**, *43*, 10866-10876; b) M. Kolbach, S. Fiechter, R. V. de Krol, P. Bogdanoff, *Catal.Today* **2017**, *290*, 2-9; c) I. Zaharieva, P. Chernev, M. Risch, K. Klingan, M. Kohlhoff, A. Fischer, H. Dau, *Energy Environ. Sci.* **2012**, *5*, 7081-7089; d) M. M. Najafpour, N. J. Moghaddam, S. M. Hosseini, S. Madadkhani, M. Holynska, S. Mehrabani, R. Bagheri, Z. L. Song, *Catal. Sci. Technol.* **2017**, *7*, 3499-3510.
[23] a) M. B. Stevens, C. D. M. Trang, L. J. Enman, J. Deng, S. W. Boettcher, *J. Am. Chem. Soc.* **2017**, *139*, 11361-11364; b) L. Trotochaud, S. L. Young, J. K. Ranney, S. W. Boettcher, *J. Am. Chem. Soc.* **2014**, *136*, 6744-6753.
[24] P. W. Menezes, A. Indra, I. Zaharieva, C. Walter, S. Loos, S. Hoffmann, R. Schlo¨gl, H. Dau, M. Driess, *Energy Environ. Sci.* **2018**, *12*, 988-999
[25] P. W. Menezes, C. Panda, S. Loos, F. Bunschei-Bruns, C. Walter, M. Schwarze, X. H. Deng, H. Dau, M. Driess, *Energy Environ. Sci.* **2018**, *11*, 1287-1298.
[26] a) M. Huang, Y. X. Zhang, F. Li, L. L. Zhang, R. S. Ruoff, Z. Y. Wen, Q. Liu, *Sci. Rep.* **2014**, *4*; b) M. Prabu, K. Ramalingam, *RSC Adv.* **2015**, *5*, 18554-18564; c) H. J. Cui, G. H. Qiu, X. H. Feng, W. F. Tan, F. Liu, *Clays and Clay Minerals* **2009**, *57*, 715-724.
[27] M. Aghazadeh, M. G. Maragheh, M. R. Ganjali, P. Norouzi, *RSC Adv.* **2016**, *6*, 10442-10449.
[28] Z. X. Liu, Y. Xing, S. M. Fang, X. W. Qu, D. P. Wu, A. Q. Zhang, B. Xu, *RSC Adv.* **2015**, *5*, 54867-54872.
[29] a) P. F. Smith, B. J. Deibert, S. Kaushik, G. Gardner, S. J. Hwang, H. Wang, J. F. Al-Sharab, E. Garfunkel, L. Fabris, J. Li, G. C. Dismukes, *ACS Catal.* **2016**, *6*, 2089-2099; b) Z. M. Chan, D. A. Kitchaev, J. N. Weker, C. Schnedermann, K. Lim, G. Ceder, W. Tumas, M. F. Toney, D. G. Nocera, *Proc. Natl. Acad. Sci. U.S.A.* **2018**, *115*, E5261-E5268; c) T. Takashima, K. Hashimoto, R. Nakamura, *J. Am. Chem. Soc.* **2012**, *134*, 1519-1527.
[30] a) H. W. Peng, I. G. McKendry, R. Ding, A. C. Thenuwara, Q. Kang, S. L. Shumlas, D. R. Strongin, M. J. Zdilla, J. P. Perdew, *Proc. Natl. Acad. Sci. U.S.A.* **2017**, *114*, 9523-9528; b) Y. T. Meng, W. Q. Song, H. Huang, Z. Ren, S. Y. Chen, S. L. Suib, *J. Am. Chem. Soc.* **2014**, *136*, 11452-11464; c) Y. F. Li, Z. P. Liu, *J. Am. Chem. Soc.* **2018**, *140*, 1783-1792.
[31] a) J. E. Post, *Proc. Natl. Acad. Sci. U.S.A.* **1999**, *96*, 3447-3454; b) J. P. Lefkowitz, A. A. Rouff, E. J. Elzinga, *Environ. Sci. Technol.* **2013**, *47*, 10364-10371; c) Y. Wang, D. Zhang, Z. B. Xiang, *J.Taiwan Inst. Chem. Eng.* **2016**, *59*, 547-552.
[32] M. Huynh, C. Y. Shi, S. J. L. Billinge, D. G. Nocera, *J. Am. Chem. Soc.* **2015**, *137*, 14887-14904.
[33] F. Song, K. Schenk, X. Hu, *Energy Environ. Sci.* **2016**, *9*, 473-477.
[34] B.-Q. Li, Z.-J. Xia, B. Zhang, C. Tang, H.-F. Wang, Q. Zhang, *Nat. Commun.* **2017**, *8*, 934.
[35] X. G. Wang, W. Li, D. H. Xiong, L. F. Liu, *J. Mater. Chem. A* **2016**, *4*, 5639-5646.
[36] B. You, N. Jiang, M. L. Sheng, M. W. Bhushan, Y. J. Sun, *ACS Catal.* **2016**, *6*, 714-721.
[37] J. Y. Li, J. Li, X. M. Zhou, Z. M. Xia, W. Gao, Y. Y. Ma, Y. Q. Qu, *ACS Appl. Mater. Inter.* **2016**, *8*, 10826-10834.
[38] L. A. Stern, L. G. Feng, F. Song, X. L. Hu, *Energy Environ. Sci.* **2015**, *8*, 2347-2351.
[39] M. Ledendecker, S. K. Calderon, C. Papp, H. P. Steinruck, M. Antonietti, M. Shalom, *Angew. Chem. Int. Ed.* **2015**, *54*, 12361-12365.
[40] N. Jiang, B. You, M. L. Sheng, Y. J. Sun, *ChemCatChem* **2016**, *8*, 106-112.
[41] N. Jiang, B. You, M. L. Sheng, Y. J. Sun, *Angew. Chem. Int. Ed.* **2015**, *54*, 6251-6254.
[42] F. Song, X. L. Hu, *Nat. Chem.* **2014**, *5*, 4477.
[43] H. F. Liang, F. Meng, M. Caban-Acevedo, L. S. Li, A. Forticaux, L. C. Xiu, Z. C. Wang, S. Jin, *Nano Lett.* **2015**, *15*, 1421-1427.
[44] Y. G. Li, P. Hasin, Y. Y. Wu, *Adv. Mater.* **2010**, *22*, 1926-+.
[45] L. L. Feng, G. T. Yu, Y. Y. Wu, G. D. Li, H. Li, Y. H. Sun, T. Asefa, W. Chen, X. X. Zou, *J. Am. Chem. Soc.* **2015**, *137*, 14023-14026.
[46] X. Y. Lu, C. A. Zhao, *Nat. Commun.* **2015**, *6*.
[47] H. Hu, B. Y. Guan, B. Y. Xia, X. W. Lou, *J. Am. Chem. Soc.* **2015**, *137*, 5590-5595.
[48] H. Y. Jin, J. Wang, D. F. Su, Z. Z. Wei, Z. F. Pang, Y. Wang, *J. Am. Chem. Soc.* **2015**, *137*, 2688-2694.
[49] J. X. Feng, S. H. Ye, H. Xu, Y. X. Tong, G. R. Li, *Adv. Mater.* **2016**, *28*, 4698-4703.
[50] S. Mao, Z. H. Wen, T. Z. Huang, Y. Hou, J. H. Chen, *Energy Environ. Sci.* **2014**, *7*, 609-616.
[51] Y. Liang, Y. Li, H. Wang, J. Zhou, J. Wang, T. Regier, H. Dai, *Nat. Mater.* **2011**, *10*, 780-786.
[52] C. C. L. McCrory, S. Jung, J. C. Peters, T. F. Jaramillo, *Journal of the American Chemical Society* **2014**, *135*, 16977-16987.
[53] X. Zhu, T. Jin, C. Tian, C. Lu, X. Liu, M. Zeng, X. Zhuang, S. Yang, L. He, H. Liu, S. Dai, *Adv. Mater.* **2017**, *29*, 1704091-n/a.
[54] Y. S. Jin, H. T. Wang, J. J. Li, X. Yue, Y. J. Han, P. K. Shen, Y. Cui, *Adv. Mater.* **2016**, *28*, 3785-3790.
[55] J. Masud, S. Umapathi, N. Ashokaan, M. Nath, *J. Mater. Chem. A* **2016**, *4*, 9750-9754.
[56] C. Walter, P. W. Menezes, S. Orthmann, J. Schuch, P. Connor, B. Kaiser, M. Lerch, M. Driess, *Angew. Chem. Int. Ed.* **2018**, *57*, 698-702.

[57] C. Walter, P. W. Menezes, S. Orthmann, J. Schuch, P. Connor, B. Kaiser, M. Lerch, M. Driess, *Angew. Chem.* **2018**, *130*, 706-710.
[58] C. W. Cady, G. Gardner, Z. O. Maron, M. Retuerto, Y. B. Go, S. Segan, M. Greenblatt, G. C. Dismukes, *ACS Catal.* **2015**, *5*, 3403-3410.
[59] R. Frydendal, L. C. Seitz, D. Sokaras, T.-C. Weng, D. Nordlund, I. Chorkendorff, I. E.L. Stephens, T. F. Jaramillo, *Electrochim. Acta* **2017**, *230*, 22-28.
[60] M. Fekete, R. K. Hocking, S. L. Y. Chang, C. Italiano, A. F. Patti, F. Arena, L. Spiccia, *Energy Environ. Sci.* **2013**, *6*, 2222-2232.
[61] M. Q. Yu, Y. H. Li, S. Yang, P. F. Liu, L. F. Pan, Le Zhang, H. G. Yang, *J. Mater. Chem. A* **2015**, *3*, 14101-14104.
[62] L. Trotochaud, J. K. Ranney, K. N. Williams, S. W. Boettcher, *J. Am. Chem. Soc.* **2012**, *134*, 17253-17261.
[63] G. Liu, J. Hall, N. Nasiri, T. Gengenbach, L. Spiccia, M. H. Cheah, A. Tricoli, *ChemSusChem* **2015**, *8*, 4162-4171.
[64] C. Hu, L. Zhang, Z. Huang, W. Zhu, Z. Zhao, J. Gong, *J. Catal.* **2019**, 369, 105-110.
[65] F. Mattelaer, T. Bosserez, J. Rongé, J. A. Martens, J. Dendooven, C. Detavernier, *RSC Adv.* **2016**, *6*, 98337-98343.
[66] H. S. Jeon, S. J. Ahn, M. S. Jee, S. S. Yoon, Y. J. Hwang, B. K. Min, *J. Electrochem. Soc.* **2016**, *163*, F3113-F3118.
[67] C. Zhao, C. Yu, H. Huang, X. Han, Z. Liu, J. Qiu, *Energy Storage Mater.* **2018**, 291-296.
[68] M. Kölbach, S. Fiechter, R. van de Krol, P. Bogdanoff, *Catal. Today* **2017**, *290*, 2-9.
[69] J. Kim, J. S. Kim, H. Baik, K. Kang, K. Lee, *RSC Adv.* **2016**, *6*, 26535-26539.
[70] S. Lian, M. P. Browne, C. Domínguez, S. N. Stamatin, H. Nolan, G. S. Duesberg, M. E. G. Lyons, E. Fonda, P. E. Colavita, *Sustainable Energy Fuels* **2017**, *1*, 780-788.
[71] D. Li, H. Baydoun, B. Kulikowski, S. L. Brock, *Chem. Mater.* **2017**, *29*, 3048-3054.
[72] A. Ramírez, P. Hillebrand, D. Stellmach, M. M. May, P. Bogdanoff, S. Fiechter, *J. Phys. Chem. C* **2014**, *118*, 14073-14081.
[73] A. C. Thenuwara, E. B. Cerkez, S. L. Shumlas, N. H. Attanayake, I. G. McKendry, L. Frazer, E. Borguet, Q. Kang, R. C. Remsing, M. L. Klein, M. J. Zdilla, D. R. Strongin, *Angew. Chem. Int. Ed.* **2016**, *55*, 10381-10385.
[74] M. P. Browne, H. Nolan, G. S. Duesberg, P. E. Colavita, M. E. G. Lyons, *ACS Catal.* **2016**, *6*, 2408-2415.
[75] Y. Xue, Z. S. Fishman, J. A. Röhr, Z. Pan, Y. Wang, C. Zhang, S. Zheng, Y. Zhang, S. Hu, *J. Mater. Chem. A* **2018**, 6, 21918-21926.
